# The insulin and ecdysone pathways as regulators of diapause termination: transcriptional and protein insights from *Pieris napi*

**DOI:** 10.1186/s12864-026-12747-2

**Published:** 2026-03-14

**Authors:** Philip Süess, Sabine Ziesemer, Rachel A. Steward, Kevin T. Roberts, Christian Müller, Christopher W. Wheat, Philipp Lehmann

**Affiliations:** 1 Department of Zoology, Stockholm University, Stockholm, 11418 Sweden; 2 Zoological Institute and Museum, University of Greifswald, Greifswald, 17489 Germany; 3 Department of Biology, Lund University, Lund, 22362 Sweden

**Keywords:** Diapause, Transcriptome, Hormonal pathways, Insulin, Ecdysone, FoxO

## Abstract

Insect diapause is a pre-programmed alternative developmental pathway induced to survive adverse environmental conditions and is characterized by an absence of development, metabolic suppression and increased stress resistance. While the hormonal regulation of diapause induction has been studied extensively, clear molecular hypotheses on how low-temperature driven diapause termination is controlled are lacking. Here we propose a hypothesis for this low-temperature driven diapause termination, which was synthesized from literature research and an experimental study where we correlate gene expression levels and protein abundance of key regulators to transitions across diapause stages. This hypothesis states that the forkhead transcription factor FoxO silences the reception of ecdysone by binding to the ecdysone receptor. This binding is gradually degraded by the accumulation of insulin signalling, in response to low temperatures, which causes the phosphorylation of FoxO. The continuous increase of sensitivity to ecdysone leads to the termination of diapause. We find that, across diapause, components of the PTTH–ecdysone axis, including PTTH signalling and downstream ecdysone-responsive genes, are present in low transcript and protein abundance, suggesting that developmental hormone signalling remains downregulated throughout diapause maintenance. Second, multiple elements of the insulin signalling pathway, including insulin-like peptides and core downstream kinases, are found in higher transcript abundance under prolonged low-temperature conditions, consistent with a role for insulin signalling in environmental sensing during diapause. Finally, FoxO shows high transcript and protein abundance at diapause initiation, followed by a gradual decline toward diapause termination, and FoxO protein abundance is negatively associated with insulin pathway activity. The main connection between the insulin pathway and FoxO, the protein kinase B (AKT) does not display the expected mRNA and protein levels. However, there are multiple factors contributing to the connection between insulin and FoxO and there might be another one involved in diapause. Simultaneously, ecdysone receptor components display stage-specific transcript–protein mismatches, indicating post-transcriptional regulation of hormonal sensitivity. Even though these results are correlational and need to be further investigated by experimental work in future studies, they support the proposed hypothesis. Summarizing, we propose a model in which cold-induced insulin signalling promotes FoxO degradation, progressively releasing inhibition of the ecdysone receptor and restoring ecdysone sensitivity, thereby timing diapause termination.

**Supplementary Information:**

The online version contains supplementary material available at 10.1186/s12864-026-12747-2.

## Author summary

For insects in the temperate regions, winter represents a challenge for development and therefore most insects enter diapause. Diapause is of particular interest because individuals can halt or dramatically slow development, extending their lifespan to several times that of their directly developing cohort. In the setup of diapause, hormones like ecdysone, prothoracicotropic hormone and insulin take a central role. However, while the mechanism terminating diapause is not yet resolved, many insects terminate diapause in response to prolonged time under low temperature conditions. The interaction between the environment and the endocrine regulation can unravel new connections between major developmental hormones and environmental conditions. Here we propose a hypothesis that states that the major developmental pathway induced by ecdysone is blocked by an accumulation of the transcription factor FoxO that silences the ecdysone receptor. During diapause and low temperature conditions insulin like peptides are produced and slowly degrade this blockage leading to an increased sensitivity to the major developmental hormone, ecdysone, which then triggers the termination of diapause and the start of development. 

## Introduction

Insects in the temperate region must endure adverse conditions during winter, when temperatures generally are too low and resources too sparse to sustain development [1]. In order to do so, many insects enter diapause, a deep resting stage in which development is arrested and the physiology is acclimatized to winter cold conditions [2]. Diapause can be obligate, always induced at a pre-determined stage of the lifecycle, or facultative, meaning an individual either continues developing directly or enters diapause depending on environmental cues. Facultative diapause consists of several phases. First is *induction*, in which an external factor such as short day-lengths, guides the insect into diapause. Induction is followed by *initiation*, in which diapause physiology is established. After this endogenously controlled *diapause* ensues, which the insect cannot leave before it has experienced specific internal or external cues, often a prolonged time at low temperature [3, 4]. Then, diapause is terminated and *post-diapause development* commences, during which development progresses in a temperature-dependent manner similar to direct development [4, 5]. The exact timing of diapause termination is essential, as terminating too early can lead to development before the unfavorable environmental conditions have passed, while terminating too late can lead to phenological mismatches between the organism and its environment [6, 7].

Insect diapause manifests primarily as an arrest in development and is controlled by major developmental hormones like prothoracicotropic hormone (PTTH), 20-hydroxyecdysone (20E), insulins, and juvenile hormones (JH). Hormonal regulation of diapause is determined by the life-stage, in immature life-stages (i.e. egg, larvae, nymphs, pupae) diapause generally affects the whole organism and central developmental hormones like 20E and PTTH are key factors in its regulation [8, 9]. The pupal diapause of the cabbage moth *Mamestra brassicae*, for example, is brought about by reduced PTTH secretion in the last larval instar. Reduced PTTH levels in the larva lead to a reduction in ecdysone secretion and this constitutes a mechanism for leading the insect into diapause instead of metamorphosis [10]. Diapause in the mature life-stage generally affects reproductive development and hormones like JH and insulin-like factors (ILPs) take a more important role [11, 12]. However, ecdysone levels are also involved in the induction of adult diapause in the leaf beetle *Colaphellus bowringi* and the ecdysone pathway is therefore a common denominator of diapause in different insect life-stages [13].

**Fig. 1:**
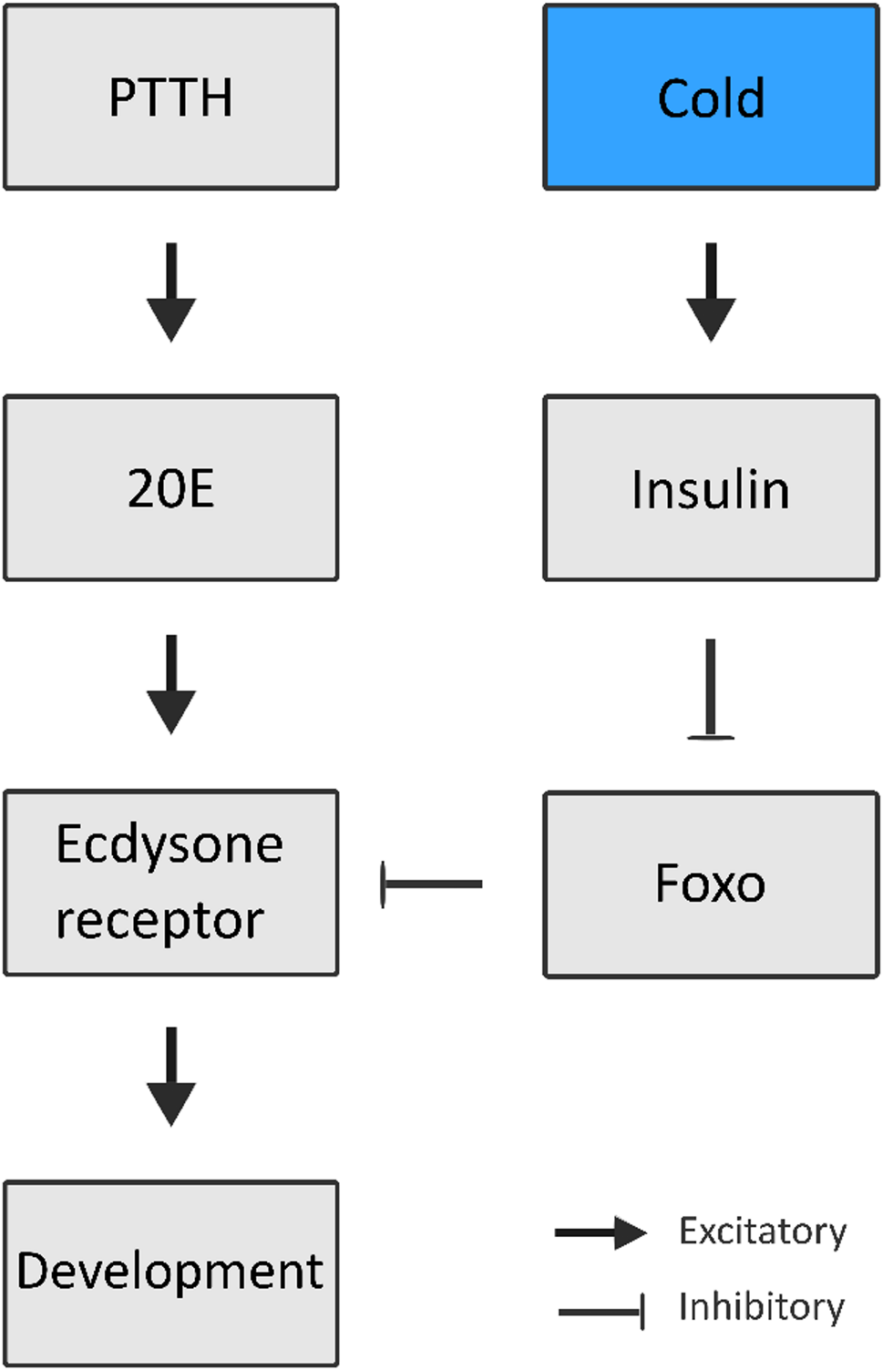
A hypothesis on the hormonal regulation of diapause termination. The hypothesis is developed from findings that the insulin pathway and the forkhead transcription factor (FoxO) are involved in the regulation of diapause in *C. pipiens* (12) and the finding that sensitivity to ecdysone returns in a time- and temperature- dependent manner in *P. napi* (15). According to the hypothesis, an abundance of FoxO early in diapause silences the ecdysteroid receptor by binding to ultraspiracle (16). This silencing is strongest at the beginning of diapause maintenance and dissipates during diapause termination. The reactivation of the ecdysteroid receptor is mediated by insulin driven phosphorylation and subsequent degradation of FoxO (20,21). The insulin pathway is activated through induction of insulin-like peptides production at low temperatures that in turn initiates phosphorylation and then degradation of FoxO. Decreasing FoxO levels ultimately release the inhibition of the ecdysteroid receptor, thereby leading to the gradual return of ecdysone sensitivity (18). Once the ecdysteroid receptors are sensitive enough for the minimal amounts of 20 Hydroxyecdysone (20E) still present in the overwintering pupae, the feedback loop between the Prothoracicotropic hormone (PTTH) and ecdysone is activated, and the pupae are potentiated for development

Current evidence suggests that diapause termination involves the same hormonal regulators, as diapause initiation. In the Chinese citrus fruit fly *Bactrocera minax* the injection of 20E can terminate diapause [8]. In the mosquito *Culex pipiens* and the flesh fly *Sarcophaga crassipalpis* the insulin pathway and FoxO are involved in diapause termination [12]. Finally in the Japanese pine sawyer *Monochamus alternatus* juvenile hormones and ecdysone are associated with diapause termination [14]. In pupae of the green-veined white butterfly, *Pieris napi*, ecdysone sensitivity returns in a time- and temperature-dependent manner, with low temperatures driving the return of ecdysone sensitivity, linking ecdysone reception to the regulation of diapause termination timing [15]. In *C. pipiens*, FoxO has been implicated, together with insulin signaling, to co-regulate diapause initiation and maintenance of adult diapause [12]. In direct development, FoxO can delay ecdysone signaling in *D. melanogaster* through the silencing of ultraspiracle (USP), one half of the ecdysteroid receptor heterodimer [12, 16]. Together these hormonal factors can be synthesized into a hypothesis on how diapause termination timing could be regulated in pupal diapause (Fig. 1)([17]. The hypothesis proposes that an increased abundance of FoxO silences the ecdysteroid receptor early in diapause and that this block gradually degrades through secretion of cold sensitive factors during diapause maintenance. One group of cold sensitive factors are the insulin-like peptides, where several members (ILP2, ILP3, ILP5) are responsive to low temperatures in *D. melanogaster* [18]. Furthermore, in *Bombyx mori* the *bombyxin-Z1* gene, an insulin, has an increased expression during low temperature exposure in diapause, while there is no increase in high temperature condition [19].

In the current study, we investigate the proposed hypothesis in *P. napi*, for which several key resources are available, such as a detailed description of the diapause trajectory as well as metabolomes and transcriptomes sampled at high frequency through diapause [22–25]. We examine the proposed hypothesis (Fig. 1) by reviewing expression of genes in the hormonal pathways relating to the biosynthesis, transport, and reception of PTTH, ecdysone and insulin signaling, as well as quantify abundances of key peptides in the hormonal pathways throughout diapause. We compare patterns in these pathways in *P. napi* to findings in well-established lepidopteran model organisms like *M. brassicae* and *B. mori*. Combining the transcriptomic profile of key hormonal pathways with protein abundance in *P. napi* uncovered the presence of mismatches between mRNA levels and the corresponding protein levels. This indicates that there are post-transcriptional events co-occurring with diapause stage transitions or environmental temperature. Nevertheless, the transcriptional profiles overall correlate well with the hormonal patterns found in other insects with pupal diapause and show a downregulation of the ecdysone pathway as well as in the PTTH signaling. Together these results support the hypothesis that the transcription factor FoxO, insulin and ecdysteroid signaling, regulating diapause termination in *P. napi*. These findings warrant further investigation into the temperature sensitivity of the expression of insulin-like peptides as well as the interaction between FoxO and the ecdysteroid receptor.

## Results

### Gene identification of factors in the prothoracicotropic - ecdysteroid axis and of insulin-like peptides in Pieris napi

**Fig. 2:**
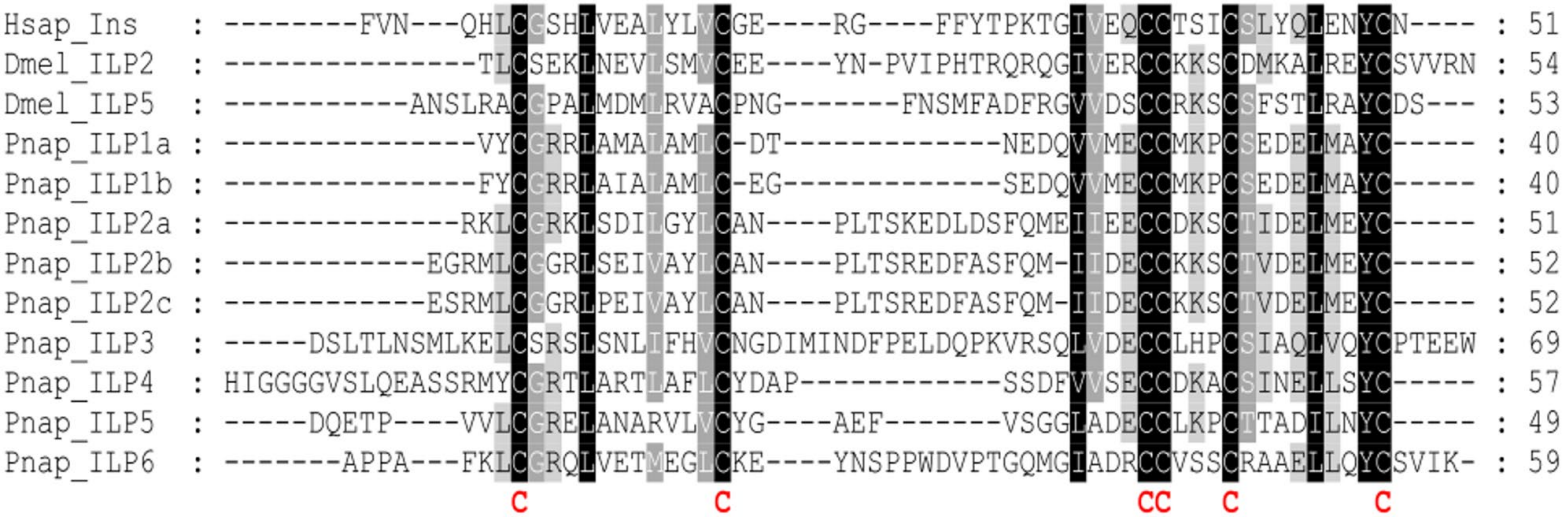
Multiple sequence alignment of insulin and insulin like peptide (ILP) sequences of *Homo sapiens* (Hsap), *Drosophila melanogaster* (Dmel_ILP2 and 5) and *Pieris napi* (Pnap_ILP1-6). A black background indicates fully conserved residues; a gray background indicates partially conserved residues. The six conserved cysteine residues giving rise to the three-dimensional structure of insulin are marked in bold and red. Abbreviations are used according to the IUPAC code

In order to identify genes that encode factors of the prothoracicotropic - ecdysteroid axis as well as putative insulin-like peptides in *P. napi* we performed BLAST searches using the reference genome data set of *P. napi* (GenBank entry GCA_905475465.2) as the target and respective sequences of different origins including both human insulin and ILPs of *D. melanogaster* sequences as queries. In total we were able to identify nine independent genes that encode putative ILPs in *P. napi*, namely Pnap_ILP1 to Pnap_ILP6. Pnap_ILP1 comprises two variants (Pnap_ILP1a and 1b) and Pnap_ILP2 comprises three variants (Pnap_ILP2a-c) whereas the remaining ILPs comprise only a single variant each. The differentiation between the Pnap_ILP variants reflects the degrees of sequence identity and similarity between and among the different ILPs. All putative ILPs of *P. napi* exhibit the typical structural features of insulins and insulin-like peptides including a signal peptide sequence. Presence and distribution pattern of six cysteine residues as well as basic amino acid residues that define putative cleavage sites for the proteolytic processing of the pro-peptides (Fig. 2). The overall degrees of sequence identities and similarities between the different putative Pnap_ILPs are in the range of 21–42% identity and 31–50% similarity. The only exceptions are the degrees between the two variants of Pnap_ILP1 (87% / 95%) and within the three Pnap_ILP2 variants (70% / 84%). It is noteworthy that almost the same degrees of sequence identity and similarity can be observed between the Pnap_ILPs and both the human insulin and ILP2 and ILP5 of *D. melanogaster*. A compilation of all pairwise degrees of sequence identities, similarities and gaps is provided in Supplementary Information Figure S2. In addition to the putative ILP sequences of *P. napi*, we were also able to identify genes that encode the *P. napi* proteins Torso and FoxO and the ecdysone receptor proteins EcR and USP. When compared to the corresponding sequences of *B. mori*, the degrees of sequence identity/similarity between the Torso protein sequences are 44% / 61% (S1, S3). The respective values for the protein sequences of the ecdysone receptor subunits EcR and USP are concordantly 79% / 88% (S1). For FoxO, the degrees are 79% / 86% (S1, S4-S6). The conserved domains for these genes were checked with the CD search on NCBI, and results show domains expected for the given genes (S2-S6).

### PTTH pathway

**Fig. 3:**
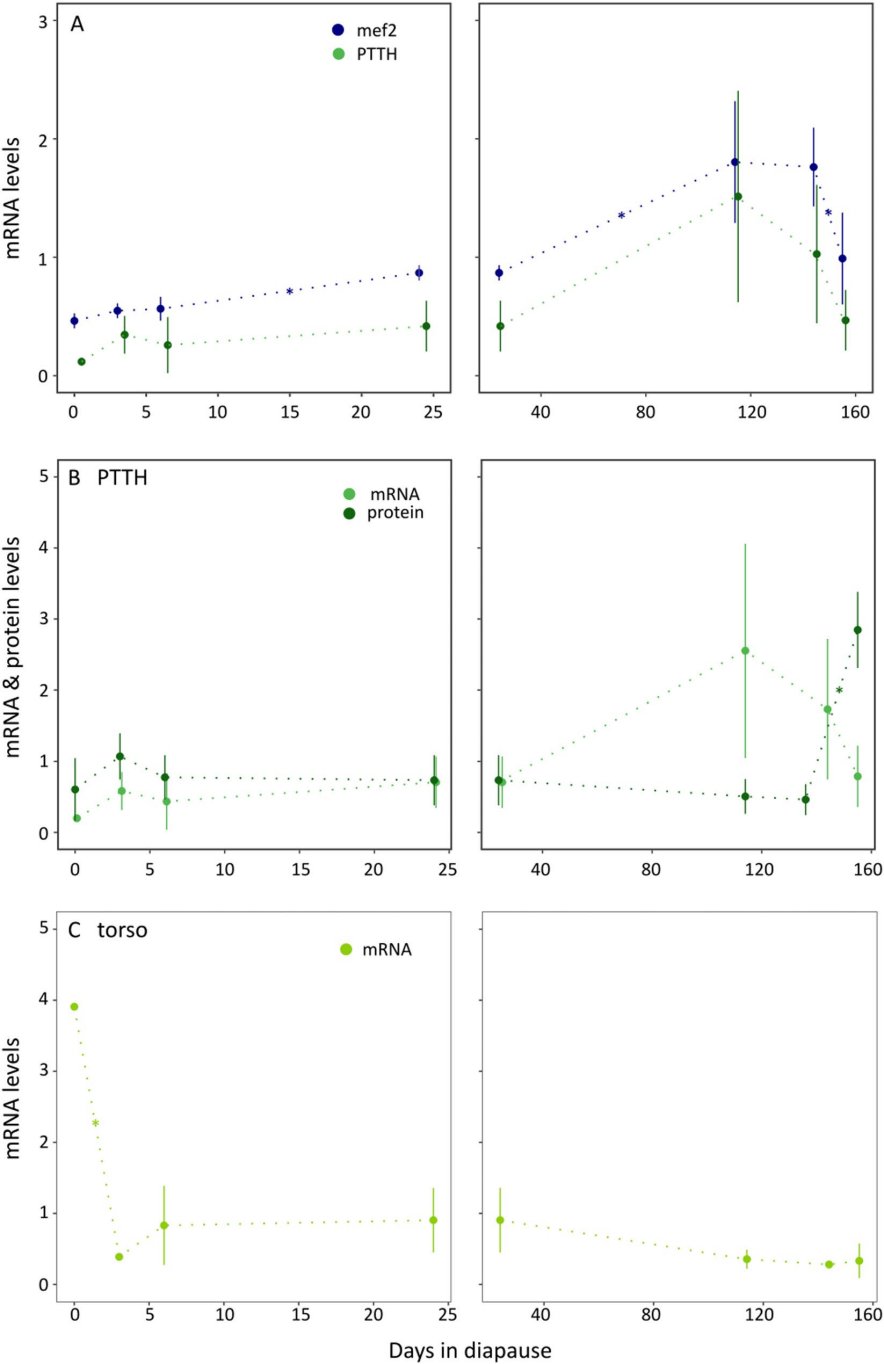
PTTH in diapause.** A**: mRNA levels of *mef2* (navy blue), a factor stimulating the transcription of the ptth gene, and ptth (light green) in the head tissue throughout diapause. The expression levels of mef2 and *ptth *show a positive correlation (cor = 1, df = 5, *p* < 0.01). Mef2 expression increases significantly from day 6 to day 24 (F = 11.41, logFC = -0.79, FDR = 0.01) and from day 24 to day 114 (F = 11.32, logFC = -0.79, FDR 0.03) and decreases from day 144 to day 155 (F = 16. 33, logFC = 0.95, *p* < 0.01). **B**: Comparison of *ptth* mRNA levels (light green), and protein levels (dark green). The PTTH protein is significantly upregulated from day 144 to day 155 when the pupae develop (diff = 133.93, *p* < 0.01). **C**: Relative read count levels of torso, the PTTH receptor. The torso gene expression is significantly downregulated from day 0 to day 3 (F = 22.84, logFC = 3.58, or*p* < 0.01). There is no correlation between the gene expressions of *ptth* and the PTTH protein levels (cor = -0.21, df = 5, *p* = 0.65). Stars indicate a significant change from the preceding time point. Normalized read count values were standardized by dividing by the average value for the corresponding read counts for the graphs. Difference in protein levels over timepoints was calculated with TukeyHSD

The expression levels of *ptth* are positively correlated with *mef2* (cor = 1, df = 5, *p* < 0.01), a factor upregulating *ptth* transcription [26]. Protein and expression levels of PTTH/*ptth* do not show any correlation during diapause (cor = -0.21, df = 5, *p* = 0.65). Expression of the PTTH receptor gene *torso* is highest at diapause initiation and then remains low until the end of diapause (Fig. 3C, detailed statistics in S7 in the supplementary materials).

**Fig. 4:**
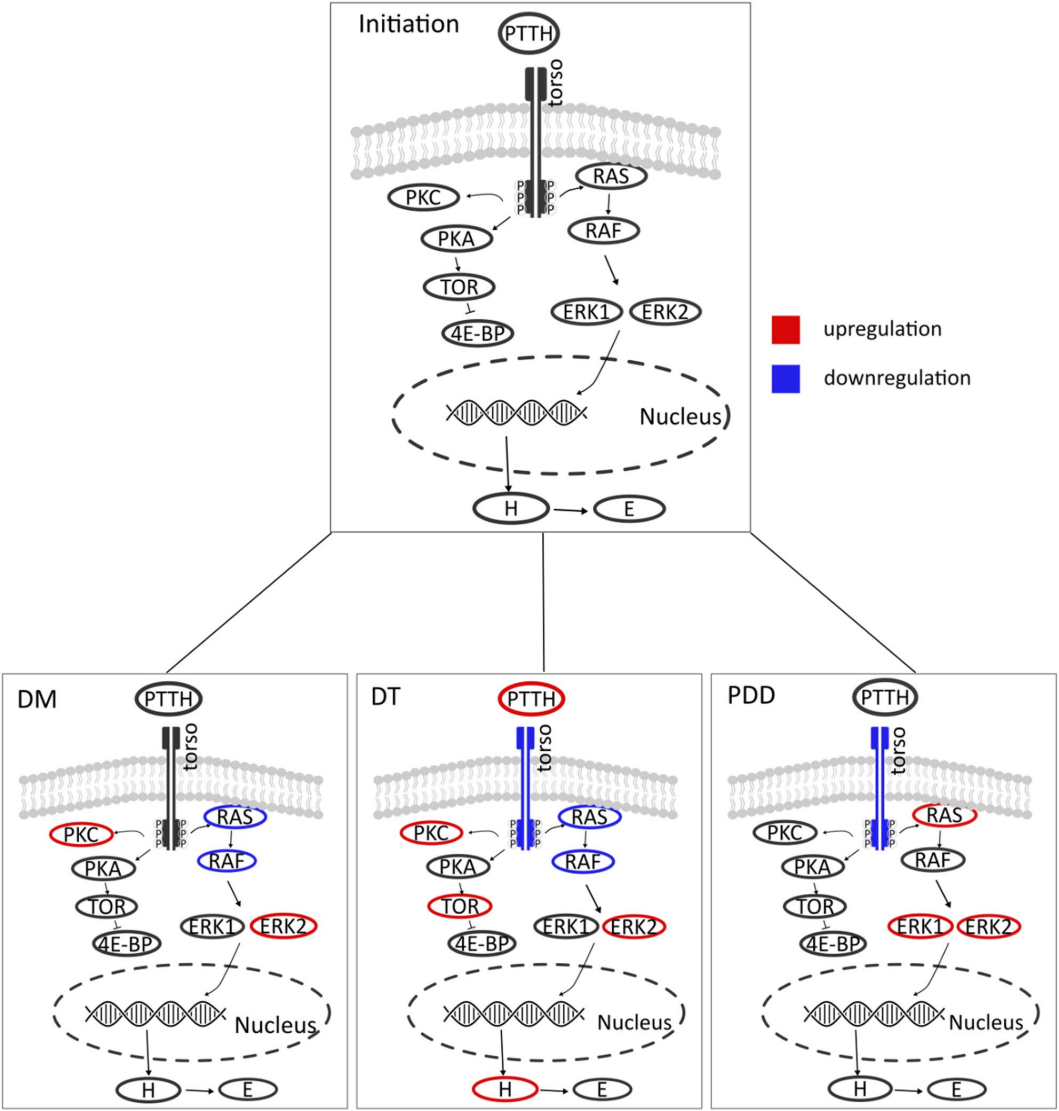
Regulation of mRNA levels in the Prothoracicotropic hormone (PTTH) pathway through diapause relative to diapause initiation. The PTTH hormonal signaling pathway: PTTH binds to Torso, a receptor tyrosine kinase and leads to the activation the ras/raf pathway. Ras/raf activates the mitogen-activated protein kinase extracellular signal-regulated kinases ERK1 and ERK2, which leads to transcription of the Halloween genes, the factors turning cholesterol into ecdysone. The developmental stages of diapause, maintenance (DM), termination (DT) and post-diapause development (PDD) were compared in a pairwise manner against the initiation stage. Red indicates a significant upregulation of expression and blue a significant downregulation of expression in the head tissue. H = Halloween genes, E = ecdysone. The ras/raf pathway is, as expected under our working hypothesis, downregulated (ras, F = 13.3, logFC = 0.44, FDR > 0.01) (raf, F = 6.74, logFC = 0.26, FDR = 0.03). The gene expression of *erk2* is upregulated during diapause (F = 17.8, logFC = -0.78, FDR < 0.01), which is counter to the expectations. The gene expression of *erk1* (F = 7.85, logFC = -0.51, FDR = 0.02) and erk2 (F = 17.6, logFC = -0.74, FDR < 0.01) are upregulated during post-diapause development

To explicitly test transcriptional differences between stages of diapause, we compared expression between diapause initiation and diapause maintenance, diapause termination and post-diapause development, focusing on downstream factors of PTTH signaling. The transcripts in the immediate pathway reacting to PTTH signaling, the *ras/raf*, are downregulated in diapause (ras, F = 13.3, logFC = 0.44, *p* < 0.01) and upregulated in post-diapause development (ras, F = 28.87, logFC = -0.58, *p* < 0.01) (Fig. 4, log_2_ fold changes and p-values Table S12-S14 in the supplements). The transcript of *erk2*, a subsequent protein kinase, is upregulated throughout diapause (F = 17.8, logFC = -0.78, *p* < 0.01) while *erk1* is only upregulated in post-diapause development (F = 7.85, logFC = -0.51, *p* = 0.02).

### Ecdysone production and reception

The factors of the Halloween group *neverland*, *shroud*, *spook*, *phantom*, *disembodied*, *shadow* and *shade* are involved in converting cholesterol into ketodiol, then ecdysone and finally into the active form 20-hydroxyecdysone [27]. The Halloween group factors show little change in expression between diapause initiation and diapause (Figure S9). After diapause has been terminated, *phantom* and *shadow*, two factors in the later part of the cascade, are significantly upregulated. After pupae have resumed development on day 155, early factors in the cascade that turn cholesterol into ketodiol are significantly downregulated, while *shadow*, the factor turning 2-deoxy-ecdysone into ecdysone, remains significantly upregulated.

**Fig. 5:**
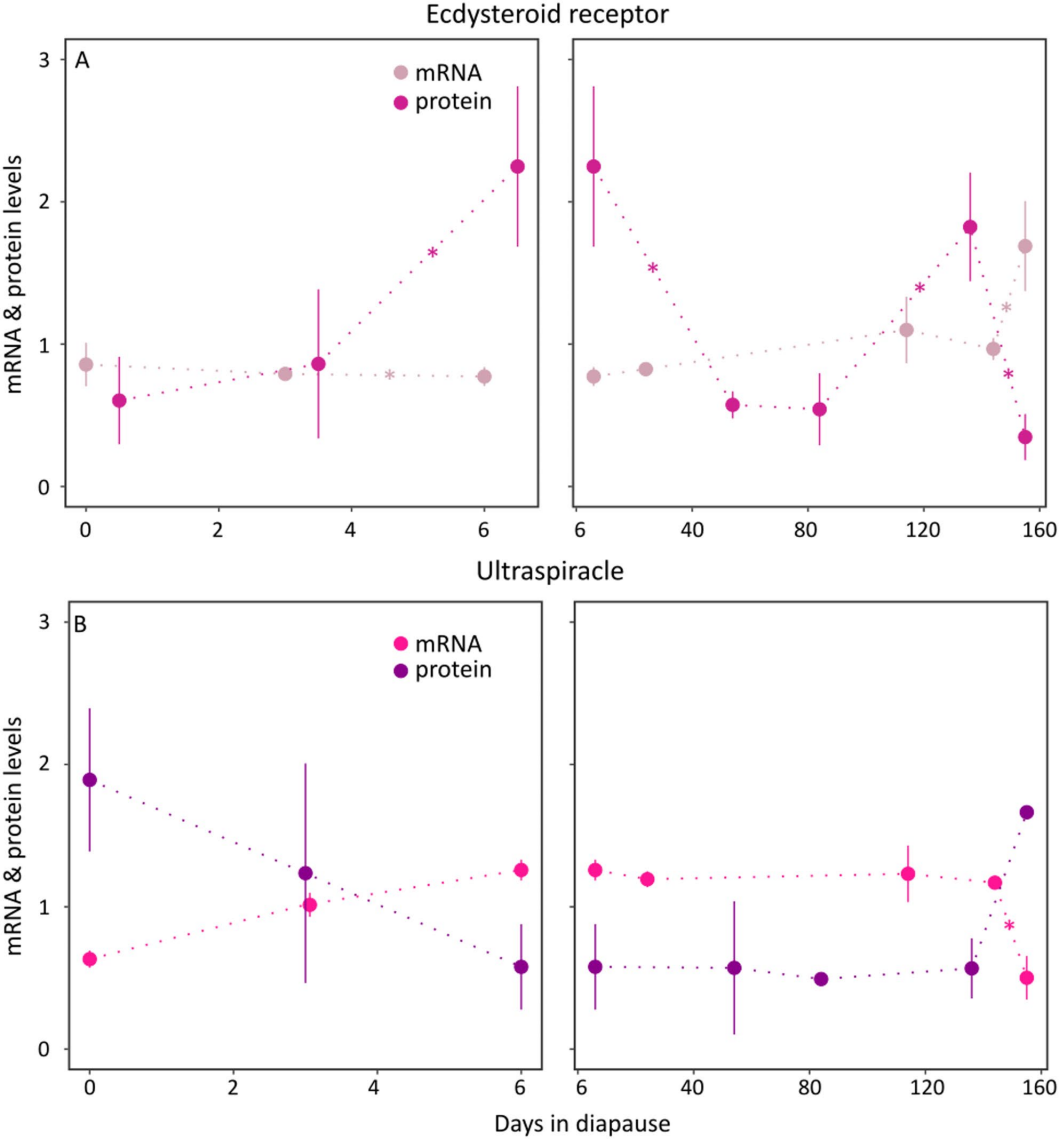
Relative protein levels of the ecdysone receptor (EcR) and ultraspiracle (USP). **A**: Relative read count levels of the *ecr* gene (magenta) and relative protein levels of EcR (rose) in the head. The expression levels of *ecr* increase significantly from day 144 to day 155 when pupa start developing (F = 6.94, logFC = -0.70, FDR = 0.03). The protein increases significantly from day 3 to day 6 (diff = 1.43, *p* < 0.01) and from day 136 to day 155 when they start development (diff = -1.53, *p* < 0.01). EcR is part of the ecdysone receptor for 20E. **B**: Relative read count levels of the *usp* gene (pink) and relative protein levels (purple) of USP in the head. Gene expression levels decreased significantly from day 144 to day 155 when the pupae started to develop (F = 51.67, logFC = 1.32, FDR < 0.01). There is a significant negative correlation between the *usp* gene expression and USP protein levels in diapause (cor = -0.95, df = 5, *p* < 0.01). USP is part of the ecdysone receptor for 20E. Stars indicate a significant change between the two timepoints

The two components of the ecdysteroid receptor dimer, *ecr* and *usp*, differ in their expression pattern throughout diapause (Fig. 5). While *usp* is upregulated early in diapause and only downregulated after development restarts, *ecr* has low expression throughout diapause and increases after development has restarted. The brain USP protein levels show a relatively high level on day 1 and day 150 of diapause, with low levels throughout diapause. For USP there is a significant negative correlation between mRNA and protein levels in the head (cor = -0.83, df = 5, *p* = 0.02). The EcR protein levels have a relative increase from day 3 to day 6 (diff = 1.44, *p* < 0.01) and from day 84 to day 136 (diff = 1.33, *p* = 0.01) and a relative decrease from day 136 to day 155 (diff = -1.53, *p* < 0.01). There is no correlation between mRNA and protein levels in either head or abdomen tissue.

### Insulin pathway and ecdysone signaling

**Fig. 6:**
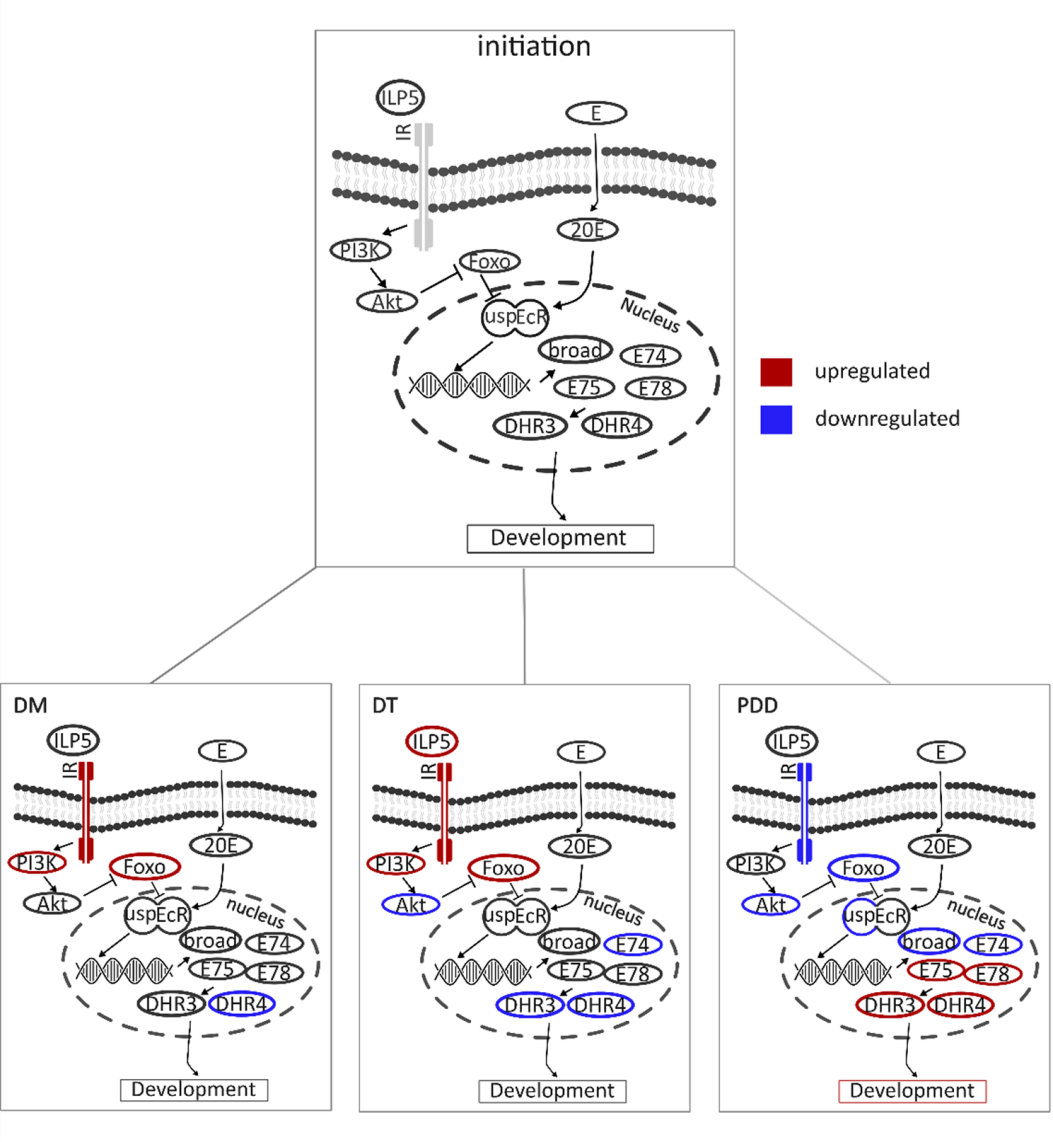
Downstream factors of the ecdysteroid signaling pathway and some of its regulators throughout diapause compared to diapause initiation. 20 hydroxyecdysone (20E) enters the nucleus and binds to the ecdysteroid receptor dimer ecdysone receptor (EcR) and ultraspiracle (USP), which then binds to the DNA and starts transcription of downstream factors. In Drosophila melanogaster, the binding of 20E to its receptor can be delayed by the binding of the forkhead transcription factor (FoxO) to USP. FoxO in turn is phosphorylated and degraded by the insulin signaling pathway. Once the ecdysone receptor dimer has bound to the DNA, transcription of the broad complex, and the ecdysone inducible genes E75, E74 and E78 commence, and they are the first factors to respond. After the early response factors, the late response factors drosophila hormone receptor 3 (dhr3) and drosophila hormone receptor 4 (dhr4) are also stimulated. These factors then initiate cell differentiation and development. Red indicates a significant upregulation and blue a significant downregulation of expression. The insulin receptor (IR) is upregulated during diapause (F = 29.7, logFC = -1.26, *p* < 0.01) and diapause termination (F = 15, logFC = -0.71, *p* < 0.01). Insulin like peptide 5 (ILP5) is upregulated in diapause termination (F = 10.61, logFC = -1.76, *p* < 0.01). And the foxo gene expression is upregulated in diapause maintenance (F = 28.2, logFC = -1.45, *p* < 0.01) and diapause termination (F = 6.89, logFC = -0.58, *p* = 0.03). The expression of the insulin receptor is upregulated in diapause (F = 29.7, logFC = -1.26, *p* < 0.01), which indicates sensitivity to insulins. E = ecdysone, 20E = 20 hydroxyecdysone, Akt = protein kinase B. DM = Diapause maintenance, DT = diapause termination, PDD = post-diapause development

Pairwise comparisons of the downstream factors of ecdysone signaling between diapause initiation and diapause maintenance, diapause termination and post-diapause development, paint a picture of the ecdysone signaling and development (Fig. 6). During diapause, few of the predicted genes are differentially expressed compared to diapause initiation. Some of the late factors like *dhr4 (F = 19.95*,* logFC = 4.2*, *p* < 0.01), *dhr3* (F = 8.64, logFC = 1.63, *p* = 0.01) or *e74* (F = 17.67, logFC = 1.4, *p* < 0.01) exhibit downregulation. After pupae have started development again, the early response factors to ecdysone signaling like the broad complex (F = 75.63, logFC = 6.44, *p* < 0.01) are downregulated but the late factors like *dhr3* (F = 24.74, logFC = -3.01, *p* < 0.01) and *dhr4* (F = 16.61, logFC = -3.35, *p* < 0.01) as well as *e75* (F = 6.56, logFC = -1.21, *p* = 0.04) and *e78* (F = 14.84, logFC = -2.68, *p* < 0.01) are upregulated. Furthermore, the expression of the insulin receptor is upregulated in diapause (F = 29.7, logFC = -1.26, *p* < 0.01), and the factor, *pI3k* (F = 8.46, logFC = -0.4, *p* = 0.02), in the insulin pathway is significantly upregulated in diapause as compared to diapause initiation. In diapause termination these factors remain upregulated and only *akt* is downregulated (F = 6.28, logFC = 0.55, *p* = 0.04). After development has restarted the insulin pathway is downregulated.

**Fig. 7:**
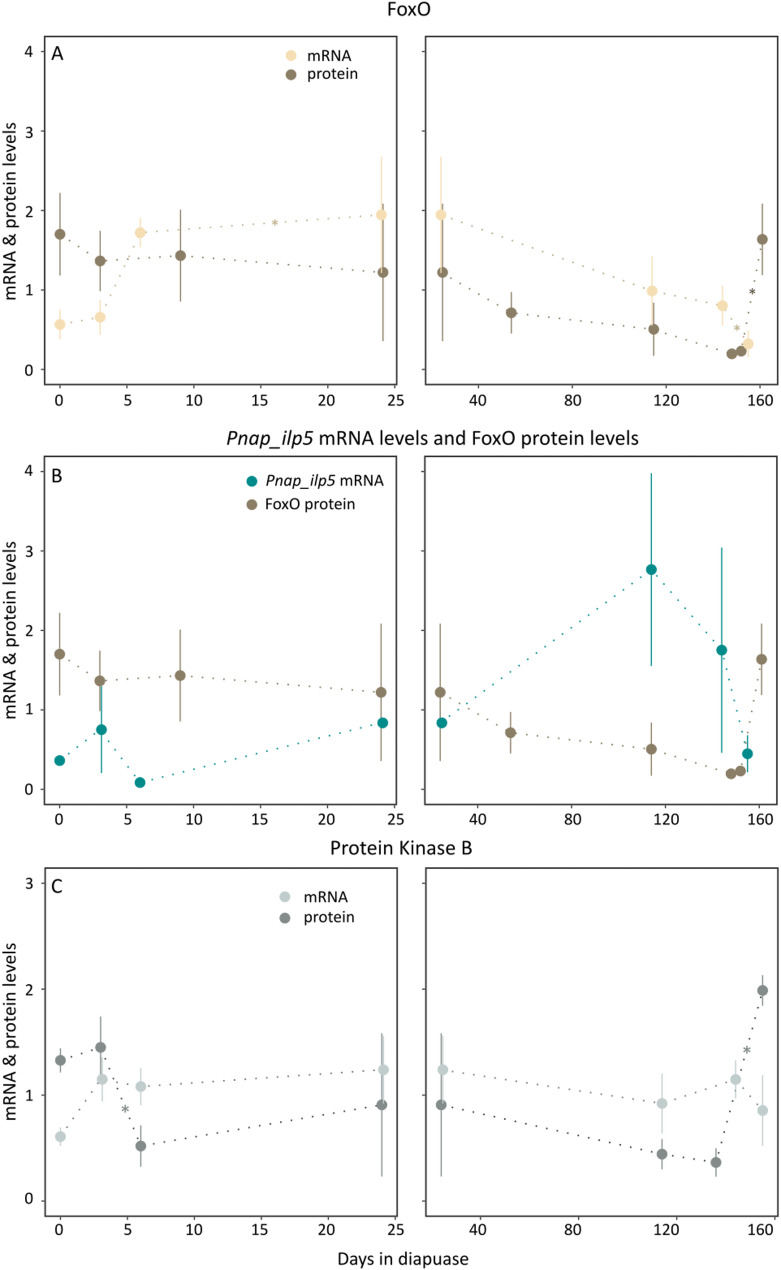
*Pieris napi* insulin-like peptide 5 and FoxO during diapause **A** Levels of foxo mRNA (beige) and FoxO protein (brown) throughout diapause in the head. The protein level increases significantly from day 152 to day 161 (diff = 210.78, *p* = 0.03). There is no correlation between the *foxo* gene expression and the FoxO protein levels (cor = 0.15, df = 5, p value = 0.75) **B** Levels of FoxO protein (brown) compared to *Pnap_ilp5* mRNA levels (cyan). Some ILPs have increased production under low temperature conditions in *Drosophila melanogaster* and are responsible for the phosphorylation and subsequent degradation of FoxO **C** Levels of protein kinase B *akt* mRNA (light grey) and Akt protein (dark grey) throughout diapause in the head. Stars indicate a significant change between the two timepoints. FoxO protein abundance is highest during diapause initiation and post-diapause development, whereas FoxO transcript levels are highest during diapause. The protein kinase B (AKT) protein levels are lower during diapause

With regards to the working hypothesis that the insulin pathway in coordination with FoxO acts as a timing mechanism on the return of ecdysone sensitivity, we find that *foxo* transcription is upregulated in diapause in the head as compared to diapause initiation (F = 18.2, logFC = -1.45, *p* < 0.01). FoxO protein levels are higher in diapause initiation and post-diapause development than in diapause (Fig. 7A). Protein levels of FoxO in the head do not correlate to mRNA levels (cor = 0.14, df = 5, *p* = 0.75). Levels of *akt* are upregulated in diapause initiation and stay elevated in diapause while Akt protein levels decrease in diapause initiation and stay low during diapause (Fig. 7C).

## Discussion

The influence of environmental conditions on hormonal pathways is crucial in many life history adaptations involving phenotypic plasticity in insects, such as the formation of gregarious versus solitary locusts [28] or long-winged versus short winged crickets [29]. Similarly, facultative diapause is a life-history adaptation involving phenotypic plasticity under hormonal control [17]. Here, we studied the influence of low temperature and time on the expression and protein levels of major hormonal regulators of diapause termination. We found that there are significant changes in the major developmental hormonal pathway’s expression guiding an insect into and out of diapause. The pattern of transcription in the hormonal pathways follows the overall pattern of transcription previously published [25], suggesting that transcription of the major developmental pathways is connected to an overall diapause gene expression program (Figure S10). The targeted analysis performed here shows that the PTTH pathway is downregulated during diapause, as also seen in other lepidoptera like *M. brassicae* or the European corn borer *Ostrinia nubilalis* [9, 30]. There is no correlation between *ptth* gene expression and PTTH protein levels in diapause, which could either be due to an enzymatic change in the diapause phenotype, enzymes are less abundant to save resources, or due to cold temperatures affecting protein translation [31]. This result raises the general question about the strength of transcript-protein correlations in vivo, as several studies show that the majority of protein levels do not correlate with their corresponding transcript levels even under standard conditions [32, 33].

The neuropeptide PTTH is, however, not the only hormone that affects the ecdysone pathway [27]. Since diapause regulation is temperature-dependent, we also studied the insulin pathway, which has shown low-temperature dependent activity in *D. melanogaster* [18]. The production of 20E, the active form of the ecdysone pathway, is the catalytic end product of seven cryptochrome p450 enzymes in the Halloween group [27]. While most of the enzymes are upregulated during diapause initiation, indicating active production of ecdysone from cholesterol, *disembodied*, which turns 2,22-dideoxy-ecdysone into 2-deoxy-ecdysone is downregulated, potentially acting as a bottleneck and the stop of ecdysone production at this stage. Importantly, injection of 20E in *P. napi* showed that there is a *time- and low-temperature-*dependent return of sensitivity to 20E, indicating that the mechanism underlying diapause termination more likely relates to the reception of ecdysone than its production [15]. While the ecdysone receptor EcR is upregulated at termination, it is also upregulated in diapause initiation and therefore it seems unlikely to drive the suppression of ecdysone signaling at the beginning of diapause. The second partner of the ecdysone receptor dimer is USP, which has an upregulated expression level, but a downregulated protein level in diapause. In *S. crassipalpis*, USP is only upregulated during diapause termination, and therefore hypothesized to be an important factor in diapause termination. This absence of USP protein in diapause, which we also see in *P. napi* would explaining the reduced sensitivity to 20E signaling, however the upregulation of USP protein only occurs after diapause has been terminated in P. napi and therefore is an unlikely candidate as the driver of the cold temperature driven mechanism to terminate diapause [34].

The ecdysone signaling pathway shows downregulation of key elements during diapause, supporting the notion that ecdysone signaling is silenced during diapause [13]. This is in agreement with Steward et al. [35] who found differently spliced variants of *e75* in *P. napi*, a response factor to ecdysone signaling, at the end of diapause that corresponds to the splice variant during the metamorphosis stage in direct development, indicating that splicing plays a role in diapause and the ecdysone pathway. Roberts et al. [36] found differently expressed miRNA associated with the ecdysone pathway, indicating that there is a wide range of factors affecting protein levels during diapause.

In *D. melanogaster* the transcription factor FoxO can bind to USP, one part of the ecdysone receptor and delay the ecdysone signal [16]. We hypothesized this to be true for *P. napi* and therefore studied the transcript and protein levels of FoxO during diapause, finding that FoxO protein levels are indeed high during diapause initiation and decrease throughout diapause. High levels of FoxO at the beginning of diapause might suppress the ecdysone signaling during a critical developmental window, thus leading the insects into diapause. Our hypothesis then proposes that after FoxO has bound to the receptor and suppressed ecdysone signaling it is removed in a time- and temperature- dependent mechanism during diapause termination. The major pathway that phosphorylates FoxO, leading to its degradation, is the insulin pathway [20]. Importantly, some members of the insulin pathway have been shown to react in a temperature-dependent manner in *D. melanogaster*, with insulin-like peptide 2, 3, and 5 (ILP2, ILP3, ILP5) produced in response to low temperature [18]. In *P. napi*, the transcription of the predicted genes *Pnap*_*ilp5*, as well as *Pnap_ilp2a* and *Pnap_ilp2b*, is upregulated during diapause, potentially in reaction to the low temperature conditions at which the pupae are maintained. However, there is a low sequence identity between the *P. napi* ILPs and the *D. melanogaster* ILPs, which might be due to the evolutionary distance between the two species. This is supported by the finding that the *B. mori* protein sequence shows a higher sequence identity to the *P. napi* sequences than the *D. melanogaster* sequences (Figure S2). The low sequence identity could indicate that a function is lost or changed, and caution is warranted in the drawing of the conclusions. Protein levels of FoxO show no correlation to the transcription levels of *Pnap_ilp5* (Fig. 7). This might be due to the fact that there are multiple factors in between. The immediate factor in the insulin pathway that phosphorylates FoxO is the protein kinase B (AKT [21]). In diapause the transcription of *akt* is upregulated, which is also concordant with our hypothesis. Although the protein level is low during diapause, little is known about the true activity of AKT, and low levels might be enough to phosphorylate large amounts of FoxO. In other species there are other factors involved in the degradation of FoxO and those factors might have a central role in diapause [20].

In this study we set out to evaluate the hormonal regulation of pupal diapause in *P. napi* against existing models of insect pupal diapause and to test a hypothesis proposed for diapause termination timing (Fig. 1). Like in other insects, diapause in *P. napi* is characterized by low levels of circulating PTTH, confirmed in this study again, and the absence of PTTH signaling throughout the endogenously controlled part of diapause [9, 15, 30, 37]. Low levels of circulating PTTH are potentially driven by post-transcriptional and post-translational factors that lead to the mismatch between protein and whole-gene expression levels during diapause, further reinforced by low temperatures interfering with enzymatic reactions [31]. Activity in the ecdysone pathway (Halloween group, *dhr4*, *dhr3*, *broad* etc.) remains low throughout diapause and major changes only take place after diapause has been terminated and development has restarted under high temperatures (20 °C), which supports the idea of the absence of ecdysone as a prerequisite for pupal diapause) [38–40]. In this study, the absence of USP during diapause can explain the low sensitivity to 20E signaling and could be one important part of the termination mechanism. Expression of *foxo* is highest at the onset of diapause maintenance and decreases towards diapause termination, and similarly the protein levels of FoxO are highest at the onset of diapause and progressively decline throughout diapause. Even though mRNA levels and the protein of FoxO do not correlate closely, both patterns show overall elevated levels at the start of diapause and a steady decline throughout diapause. This correlation fits with the hypothesis that FoxO silences the ecdysteroid receptor during diapause initiation and that the silencing is removed in a time- and low-temperature-dependent manner. The activity of the insulin pathway throughout pupal diapause shows an expected pattern but does not correlate with the decline in FoxO protein levels and *Pnap*_*ilp5* shows a temperature- or diapause-dependent increase in expression starting on day 24. While these are all correlational indicators that need further experimental tests, they support the hypothesis of co-regulation of diapause termination timing by FoxO, insulin and the ecdysteroid receptor. Future experiments need to, among others, directly establish whether FoxO interacts with the ecdysteroid receptor in *P. napi* and whether the release of insulin-like peptides is stimulated by low temperatures.

## Methods

### Sampling

**Fig. 8:**
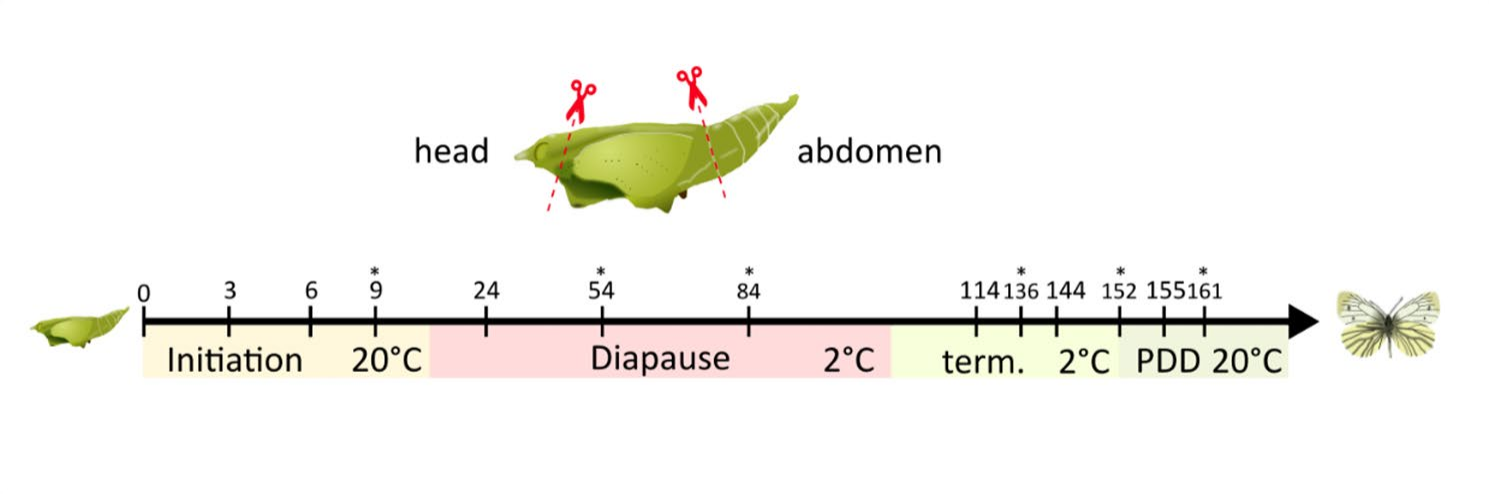
Sampling scheme for RNAseq. Head and abdomen tissue was sampled from four female green-veined white *Pieris napi* pupae going through direct (non-diapause) and diapause development on day 0, 3 and 6 after pupation. These timepoints reflect diapause initiation for diapause pupae. On day 14, diapause pupae were moved to 2°C, which are diapause-terminating conditions (23). Sample time point 24 represents the early endogenously controlled diapause maintenance phase, before diapause has been terminated. Pupae on days 114 and 144 after pupation have terminated diapause but do not show development due to the low ambient temperature (2°C). On day 144, the temperature was raised to 10°C for a week and then to 20°C on day 151. Pupae sampled on day 155 after pupation are in post-diapause development. For protein sampling certain time points were replaced with individuals from another correlating time point. In diapause initiation day 6 was replaced with day 9, day 24 with days 54 and 84 and day 144 with days 136, 148 and 152 and day 155 with day 161. Dev. = development, term. = diapause termination, PDD = post-diapause development. The * indicates replacement time points for the protein sampling

RNAseq reads for this analysis were assembled by Pruisscher et al. [24] and are archived on NCBI under Bioproject PRJNA684967. For that study, heads and abdomens of female *P. napi* pupae from a population in Skåne, southern Sweden (Kullaberg, 56°18`N, 12°27`E and Vejbystrand, 56°, 18`N, 12°46`E, Fig. 8), were used. In order to induce diapause, larvae were reared under short day length conditions (light: dark, 10 h:14 h, at 20 °C)([41] and sampled on day 0, 3, 6, 24, 114, 144 and 155 after pupation. The timepoints were chosen to reflect the different diapause stages, days 0, 3 and 6 for diapause initiation, day 24 for diapause, day 114 and 144 for diapause termination and day 155 for post-diapause development. The pupae were kept in the same short-day conditions (light: dark, 10 h:14 h, at 20 °C) for 14 days after pupation before being moved to 2 °C and complete darkness. After day 144, the pupae were moved from 2 °C to 10 °C and on day 151 the temperature was raised to 20 °C. All pupae were sampled between 10:00 and 13:00 to reduce circadian effects on the transcriptome [25]. Due to insufficient samples from this original rearing for the estimation of protein levels, a few time points were replaced with insects sampled from a Stockholm population reared in the same way as described above. The timing of the sampling in the Stockholm population was adapted to match the sampling in the Skåne population according to the slight shift in diapause termination timing found in Posledovich et al. [42] and based on results from additional experiments. For PTTH protein levels, day 136 from Stockholm replaced day 144 of Skåne. For USP and EcR protein levels, days 54, 84, and 136 from Stockholm replaced days 24 as diapause time point and 144 as diapause termination point respectively. For AKT protein levels, day 136 from Stockholm replaced day 144. Finally, for FoxO protein levels, day 9 from Stockholm replaced day 6 as diapause initiation from Skåne for diapause initiation, additionally to day 24, day 54 was added, and in diapause termination, post-diapause development, days 148, 152, and 161 replaced day 144 and day 155 respectively.

### Alignment

We leveraged an alignment of the RNAseq reads previously generated by Steward et al. [35]. In short, the mRNA libraries were mapped to the ilPieNapi1 genome from the Darwin Tree of Life project (DTOL) using hisat2 [43] specifying reverse strandedness, and otherwise default parameters. The featureCounts function (RSubread v. 3.16) [44, 45] was used to quantify expression at the gene level, focusing on coding genes, and did not allow reads to overlap multiple gene features, eliminating possible chimeric transcripts. We quantified gene expression using a protein-based annotation generated with BRAKER2 [46, 47] and previously used to compare gene expression, alternative splicing and miRNA expression through *Pieris napi* diapause. A detailed description of the annotation can be found in Steward et al. [35]. The annotation and code used to generate it are archived on GitHub (see Data Availability).

### Selection of target genes and pathways

The PTTH-ecdysone signaling pathway is involved in the regulation of diapause of many lepidopteran species, including *M. brassicae* [9, 48], the tobacco budworm *Heliothis virescens* [37], the beet moth *Scrobipalpa ocellatella* [38], the corn earworm *Helicoverpa zea* [49] and in *P. napi*, the study species used for this experiment [15]. Therefore, we focused on known factors in the PTTH pathway (for more information see Table S1 and protein sequence alignment in S3 in the supplements and Süess et al. [15].

The ecdysone pathway consists of the Halloween gene cascade, which turns cholesterol into ecdysone and finally 20E, the active form of the hormone [27]. 20E then binds to the ecdysteroid receptor consisting of an EcR and USP dimer [50]. The predicted protein sequences in *P. napi* show a 79% and 79% sequence identity to the respective protein sequences in *B. mori* (S1, S4 and S5). The binding of 20E to the ecdysteroid receptor causes a cascade of factors to be upregulated, which are all involved in the progression of development [51]. The PTTH-ecdysone signaling pathways interact with a positive feedback loop at low to intermediate levels of ecdysone, while high levels of ecdysone inhibit the transcription of the *ptth* gene in a negative feedback loop [52].

The insulin pathway is involved in the regulation of adult diapause in several insects, such *C. pipiens*, *Bombus terrestris* and *D. melanogaster* [12, 53, 54]. The insulin pathway transfers information about an insect’s nutritional state and respond to low temperatures in *D. melanogaster*, and both are essential factors in diapause regulation in several species [18, 55]. The transcription and secretion of several members of the insulin-like peptides (ILPs) show an increased transcription as well as an increased secretion in *D. melanogaster* under low temperature conditions and therefore could act as cold-sensors [18]. These ILPs binds to the insulin receptor (IR) and start a cascade of factors including the phosphoinositide 3-kinase (PI3K) and the protein kinase B (Akt) involved in the phosphorylation of, among many other targets, FoxO and its subsequent degradation [20, 56].

The forkhead transcription factor FoxO was chosen as it is involved in diapause regulation of *C. pipiens* [12] and interacts with both the PTTH pathway [57] as well the ecdysone pathway [16]. FoxO is involved in stress resistance, affects metabolism and cell cycle arrest, which are all hallmarks of diapause and constitutes a link between the environment and endogenous functions like gene expression [20, 58]. The predicted FoxO protein sequence in *P. napi* shows an 80% sequence identity with the FoxO protein sequence in *B. mori* (S1 and S2.4).

### Identification of target genes

In the first step to identify target genes (see table S1 for full list) in the count data, amino acid sequences were collected for each gene from well-described model organisms such as *D. melanogaster*,* B. mori* and *M. sexta*, respectively. We used the model organism sequences to get the sequence from the *P. napi* Darwin Tree of life genome. We generated a BLAST database from the *P. napi* genome (ilPieNapi4.1), with the target gene sequences, selecting the sequence with the highest Max Score as the putative ortholog. The *P. napi* nucleotide sequences were then blasted against a BLAST database of the amino acids predicted by the in-house protein-informed BRAKER2 gene annotation. The annotated gene locus with the highest score was identified as the putative target ortholog in our annotation and used in downstream analyses of gene expression. As a final confirmation of these loci, the nucleotide sequence was extracted from the identified locus of the *P. napi* genome and used in the standard NCBI BLAST (BLAST + 2.14.0: April 25, 2023) nucleotide database to confirm that the highest scoring hits were, in fact, the target gene. Additionally, we checked the conserved domains in the genes to see whether they have the functional domains with CD search on NCBI (S2-S6). In a second step the “proteins of interest” (FoxO, ultraspiracle, ecdysteroid receptor, insulin receptor and the insulin-like peptides) sequences were used to conduct tblastn searches with adjusted parameters (expect threshold: 1; word size: 2) against the genome of *P. napi* in order to identify putative paralogs with low degrees of overall sequence identity/similarity.

### Differential gene expression

All the raw read count data (not just our manually curated list) were normalized with edgeR (v. 3.36.0) [59, 60] calculating normalization factors with calcNormFactors using trimmed marginal means (TMM); dispersion was estimated with estimateDisp. We did not filter genes based on expression as we were working on specific genes and hormones that might have a low read count. In addition to investigating normalized expression across all time points, we quantified differential expression between stages. A quasi-likelihood negative binomial generalized log-linear model glmQLFit was fitted to the data, and expression was compared pairwise between diapause stages: diapause initiation (day 0, day 3 and day 6) against diapause maintenance (day 24), diapause initiation against diapause termination (day 114, day 144) and diapause initiation against post-diapause development (day 155).

### Extraction of proteins and western blot analyses

To quantify PTTH, EcR, USP and Akt levels at day 0, 3, 6, 24, 114, 144 and 155, pupae were taken from the same population as the pupae for the RNAseq data apart from some missing time points that were supplemented with pupae in the same diapause stage. Pupae from a Stockholm population (Stockholm University, 59°21`N, 18°03`E) sampled on day 9, 54, 84 and 136 of diapause were chosen as replacement for respective days from the Skane population. We cannot rule out that there are molecular differences between populations, however, the populations show physiological similarities in metabolic patterns as well as diapause [4, 24]. To quantify FoxO levels, samples were taken from the same experiment as the pupae for the RNAseq. Here we used the Stockholm population again to cover some missing developmental stages: day 9, 54, 148, 152 and 161. For EcR and USP we used day 54, 84 and 136 to replace the original timepoints of 24 and 114. For each time point, hormone and tissue, 3 biological replicates were used.

Heads and abdomens were cut from frozen pupae and homogenized for 30 s on ice using a T8-Ultraturrax homogenizer (IKA-Werke, Staufen, Germany) in 10 µl of lysis buffer [100 mmol/l KCl, 20 mmol/l NaCl, 2 mmol/l MgCl_2_, 0.96 mmol/l NaH_2_PO_4_, 0.84 mmol/l CaCl_2_, 1 mmol/l EGTA, 0.5% (v/v) Tween 20, 25 mmol/l HEPES (free acid), pH 7.2] containing protease inhibitors (0.31 mmol/l aprotinin, 4.21 µmol/l leupeptin, 2.92 µmol/l pepstatin, 1 mmol/l PMSF and 0.33 mmol/l ortho-vanadate) per mg of tissue fresh mass, respectively.

Homogenates were centrifuged 2x at 16,200 g and 4 °C for 10 min to remove debris and protein concentrations were measured [61] using BSA-solution as a standard. Aliquots were prepared for long term storage at − 80 °C in an equal volume of SDS sample buffer [50 mmol/l Tris (free base), 1% SDS, 0.2% bromophenol blue, 4% β-mercaptoethanol, 40% glycerol, pH 6.8]. After thawing the samples and heating to 95 °C for 3 min, 15 µg of total protein were loaded and separated in each lane of a 10% SDS-polyacrylamide gel in a mini-gel apparatus (Bio-Rad, Munich, Germany). ROTI^®^Mark STANDARD (Roth, Karlsruhe, Germany) was used as a molecular mass marker to determine the apparent molecular masses of proteins of interest. Proteins were transferred to nitrocellulose membrane (NC 2, SERVA, Heidelberg, Germany) by semi dry blotting [62] (S14). Membranes were stained with Ponceau S for total protein normalization [63].

Western blotting was performed using specific primary antibodies against FoxO (1:1000, Cell Signaling Technology, Frankfurt/M., Germany), Akt (1:1000, Cell Signaling Technology, Frankfurt/M., Germany) and PTTH (1:1000, by Dr. J. Pineda Antikörper-Service, Berlin, Germany, previously described Süess et al., 2022), EcR (2.5:1000, 10F1-s, deposited to the DSHB by Riddiford, L.M.), USP (OriGene, Herfurt/Germany) HRP-linked secondary antibodies (1:6,000, Cell Signaling Technology, Frankfurt/M., Germany) and enhanced luminescence reagents (Biozym, Oldendorf, Germany). Signals were recorded using an IntasChemostar ECL imager (Intas, Göttingen, Germany).

The evaluation of Western blot membranes was performed using Phoretix 1D (totallab, Newcastle upon Tyne, UK) software. First, the protein bands of interest on each membrane were identified, and their band intensities (optical density/volume) were quantified after background subtraction. For each blot, the intensities of all detected bands of the protein of interest (e.g. PTTH) were used to calculate a mean band intensity, which was set to 100%. The intensity of each individual band was then expressed as a percentage of this mean, resulting in the relative band intensity of the respective protein. In parallel, the total protein content was analyzed using the Ponceau S–stained membranes [63]. For each lane, the intensities of all Ponceau-stained bands were quantified with Phoretix 1D and summed to obtain the total protein signal per lane. For each blot, the mean total Ponceau intensity of all lanes was calculated and set to 100%. The intensity of each individual lane was then expressed as a percentage of this mean, yielding the relative total protein amount per lane (which theoretically should be close to 100%, as 15 µg protein were loaded per lane). To normalize for loading differences, the relative band intensity of the specific protein (e.g. PTTH) was divided by the relative total protein intensity of the corresponding lane derived from the Ponceau staining. This normalization step relates the abundance of the protein of interest to the total protein content of the sample and serves as loading control. The final values are expressed as:

$$relative\,band\,intensity\,/\,Ponceau\,\left[\%\right]$$

Where applicable, the average of the technical replicates was calculated to obtain one value per biological replicate, and subsequently, the average of the biological replicates was determined and represented graphically.

## Supplementary Information

Supplementary Material 1.

## Data Availability

Gene expression was estimated using archived *Pieris napi* RNA-seq data from Bioproject PRJNA684967 (https://www.ncbi.nlm.nih.gov/bioproject/?term=PRJNA684967). Reads were mapped to the ilPieNapi1 *P. napi* genome assembly from the Darwin Tree of Life Project (GCA_905231885.1; https://www.ncbi.nlm.nih.gov/datasets/genome/GCA_905231885.1/). The gene annotation and code used to quantify gene expression can be found on GitHub (https://doi-org.ezp.sub.su.se/10.5281/zenodo.10277672). Gene location data, conserved domains and original western blot files are accessible on Biostudies (S-BSST2695).

## **Abbreviations**

FoxO: Forkhead transcription factor
PTTH: Prothoracicotropic hormone
AKT: Protein kinase B
20E: 20-hydroxyecdysone
JH: Juvenile hormone
ILP: Insulin like peptide
USP: Ultraspiracle
EcR: Ecdysone receptor
Mef2: Myocyte enhancer factor 2
E: Ecdysone
PKC: Protein kinase C
PKA: Protein kinase A
DM: Diapause maintenance
DT: Diapause termination
PDD: Post-diapause development
ERK: Extracellular signal-regulated kinase
Pnap: *Pieris napi*
DHR: Drosophila hormone receptor
PI3K: Phosphoinositide 3 kinase
IR: Insulin receptor
