# Supplementary material for: The insulin and ecdysone pathways as regulators of diapause termination: transcriptional and protein insights from Pieris napi
Source: BMC Genomics. 2026 Mar 14;27:363. doi: 10.1186/s12864-026-12747-2 (PMC13063973; doi:10.1186/s12864-026-12747-2)
Supplement: Supplementary file 1 — Supplementary Material 1. [file 12864_2026_12747_MOESM1_ESM.docx]

**Supplementary information: The Insulin and Ecdysone Pathways as Regulators of Diapause Termination: Transcriptional and Proteomic Insights from *Pieris napi***

Philip Süess^1^, Sabine Ziesmer^2^, Rachel A. Steward^3^, Kevin T. Roberts^1,2^, Christian Müller^2^, Christopher W. Wheat^1^, Philipp Lehmann^1,2^

^1^ Department of Zoology, Stockholm University, 11418 Stockholm, Sweden

^2^ Zoological Institute and Museum, University of Greifswald, 17489 Greifswald, Germany

^3^ Department of biology, Lund University, 223 62 Lund, Sweden

**Corresponding author:** Philipp Lehmann, email: philipp.lehmann@zoologi.su.se

Philip süess, email: philip.suess@zoologi.su.se

**Supplementary methods**

*Ordination and hierarchical clustering of expression across samples and genes*

Reads were normalized and a principal component analysis (PCA) as well as a clustering analysis was performed. For these the raw read counts were normalized using edgeR cpm() and standardized using the standardize function in R [1]. The PCA was produced with the prcomp() function from the stats package on head read counts and abdomen read counts separately. The number of clusters for the fuzzy clustering was calculated with the minimum centroid distance to optimize number of clusters [2]. The fuzzy clustering was created with the Mfuzz package in R [2, 3]. The logFC heat maps were produced with the ggplot2 package [4].

**Supplementary Results**

The expression patterns of the target genes throughout diapause are described by five clusters in the head and by six clusters in the abdomen (Figure S2, Table S3). In the head, cluster H1 shows a steady increase in expression throughout diapause with highest levels at day 144 (Figure S2C). This cluster notably contains *ptth* and *ilp2*. The pattern of *ptth* expression aligns with the findings of more PTTH in post-diapause pupae than in diapausing pupae and corresponds to findings in both *M. brassicae* and *P. napi* [5, 6]. In cluster H2, genes show a higher expression at the beginning and the end of diapause. This cluster contains the *ecdysone* *receptor* and several indicators of development, like *wnt10b* and *smad3*. The ecdysone receptor is one part of the ecdysteroid receptor heterodimer and its absence during diapause could explain the reduced sensitivity to 20E in *P. napi* during the early part of diapause and the increased sensitivity to 20E injections in the latter parts of diapause in *B. minax* [6, 7]. Cluster H3 contains some indicators of development, *wnt5*, *frizzled* and *plk1*, and shows the highest gene expression at the beginning of diapause with a reduced level of expression in diapause maintenance. While contradictory to the notion of diapause as an overall shut-down of development, it is important to note that diapause initiation seems to require some development before the full diapause phenotype is manifested [8]. However, when pupae transition to diapause maintenance, expression levels of these indicators decrease, and only return after diapause has been terminated [9]. In cluster H4, elevated gene expression during the first 6 days of diapause are followed by a steady decline until day 144. This cluster contains *torso* and factors downstream of ecdysone signaling, such as *e74*, *e78* and *dhr4*. This is potentially a remnant of ecdysone signaling from early pupation as the pupae have most likely subsided the ecdysone signaling, for ecdysone injections have been shown to terminate diapause precociously [6, 10, 11]. Cluster H5 shows a steady increase in gene expression until day 24 of diapause after which it steadily decreases again. In this cluster are the *insulin* *receptor*, *FoxO* and some downstream factors of ecdysone signaling, *broad* and *e75*. Upregulation of the *insulin* *receptor* early in diapause might reflect a need for increased receptor abundance as *ilp2* expression is increasing throughout the progression of diapause. The upregulation of *FoxO* expression could explain the silencing of the ecdysteroid receptor, which is at its least sensitive at the beginning of diapause maintenance [12].

In the abdomen, cluster A1 shows increased expression in the beginning of diapause with the lowest point during diapause maintenance and an increase towards diapause termination. This cluster contains the *ras*/*raf* factors and *wnt10b*, an indicator of development [13]. Together, these suggest low levels of activity in the PTTH pathway and little to no development during diapause [14]. Cluster A2 shows a slight increase of gene expression at day 24 of diapause, *FoxO* follows this pattern, and the highest expression coincides with the lowest sensitivity to 20E in diapause [6]. The transcriptional patterns in cluster A3 show high levels on day 3 of diapause, followed by low levels at day 6 and 24 and then an increase with termination. Representatives of this cluster are indicators of development like *smad3* and *plk1*, which are involved in the differentiation of cells [15]. A steady decline in expression throughout diapause forms the pattern in cluster A4, which includes *torso*, indicating that the potential to receive PTTH signaling is present throughout diapause [14]. Even though the PTTH receptor is transcribed, there could be further factors inhibiting the reception of PTTH in the prothoracic gland (PG), like myosuppressin, which has been shown to inactivate the PG during diapause induction in *Mamestra brassicae* [16]. Cluster A5 consists of an increase in gene expression during early diapause maintenance and then a decrease towards diapause termination. This cluster contains *broad* and *e75,* downstream factors of ecdysone signaling, as well as *ultraspiracle*. This could indicate that low level ecdysone signaling takes place throughout diapause. Cluster A6 shows a steady increase in expression of genes towards the end of diapause. It contains the *insulin* *receptor*, *smad1* and *axin*, indicators for development, as well as the *ecdysone* *receptor*. This pattern in *ecdysone receptor* expression indicates that sensitivity to 20E is lowest at the beginning of diapause and potentially increases in a time dependent manner towards diapause termination, correlating with the return of ecdysone sensitivity described in Süess et al. (2022).

**Supplementary Tables**

**Table S1: Table with the curated predicted gene list for the ecdysteroid pathway, PTTH pathway, Insulin pathway, Juvenile Hormones and correlated factors.** Gene name with the abbreviation followed by which hormonal pathway it was associated for this study, the function of the gene and the references.

| **gene name** | **gene** | **Identity^a^** | **function** | **source** |
| --- | --- | --- | --- | --- |
| prothoracicotropic hormone | PTTH | 49%° | stimulation of ecdysone production | [14] |
| torso | torso | 46%* | PTTH receptor | [14] |
| ultraspiracle | usp | 78%* | 20E receptor | [17] |
| ecdysone receptor | EcR | 81%* | 20E receptor | [17] |
| shade | shd | 48% | p450 enzyme involved in 20E production | [18] |
| shadow | sad | 34% | p450 enzyme involved in the E production | [19] |
| neverland | nvd | 54%* | p450 enzyme turning cholesterol into 7-dehydro-cholesterol | [20] |
| phantom | phm | 38% | p450 enzyme turning ketodiol into 2,22-dideoxyecdysone | [21] |
| disembodied | dib | 43% | p450 enzyme turning 2,22-dideoxyecdysone into 2-deoxyecdysone | [22] |
| spook | spook | 52% | p450 enzyme turning 7-dehydro-cholesterol into ketodiol | [23] |
| shroud | shroud | 38% | p450 enzyme turning 7-dehydro-cholesterol into ketodiol | [20] |
| broad | broad | 65%* | first downstream factor of 20E signaling, differentiation of adult structures | [24, 25] |
| protein kinase A | PKA | 95% | downstream factor of PTTH signaling, cryoprotection | [14] |
| forkhead transcription factor | FOXO | 79%* | stress response, silencing of ecdysone receptor | [12, 26] |
| insulin receptor | IR | 23% | receptor of insulin and ILPs | [27] |
| krüppel | Krüppel | 81%* | downstream factor of JH signaling, repressor of metamorphosis | [28] |
| wnt 1 | wnt1 | 90%* | indicator of development, involved in hox gene regulation | [29] |
| protein kinase B | AKT | 66% | downstream of insulin pathway, phosphorylation of Foxo | [30, 31], |
| extracellular signal-regulated kinase 1 | ERK1 | 64%* | downstream of PTTH, cell adhesion cell cycle progression,differentiation | [14], [32] [33] |
| protein kinase isoform 2 | PKA2 | 95% | downstream of PTTH, cryoprotection | [34], [14] |
| protein kinase isoform 3 | PKA3 | 95% | downstream of PTTH, cryoprotection | [34], [14] |
| raf | Raf | 59% | downstream of PTTH, PTTH signal transduction | [14] |
| extracellular signal-regulated kinase 2 | ERK2 | 85%* | downstream of PTTH, cell adhesion cell cycle progression,differentiation | [14], [33] |
| target of rapamycin | TOR | 64%* | downstream of PTTH signaling, cell growth coordination | [14], [35] |
| phosphoenolpyruvate carboxykinase 1 | PEPCK1 | 66% | regulator of the rate of gluconeogenesis | [36] |
| polo like kinase 1 | plk1 | 87%* | promotion of cell division, centromer maturation, regulation of cytokinesis | [37] |
| protein kinase C 1 | PKC_x1 |  | downstream of PTTH signaling, involved in signal transduction cascades | [14], [38] |
| insulin like peptide 2b | ILP2b | 23% | insulin signaling | [27] |
| prothoracicostatic peptide | PTSP |  | inhibition of ecdysteroid genesis | [39] |
| phosphatase and tensin homolog | PTEN | 46%* | regulation of p-AKT levels | [40] |
| wnt 5 | wnt5 | 80%* | indicator of development, axon guidance | [41] |
| wnt 6 | wnt6 | 88%* | indicator of development, maxillary palp formation | [42] |
| wnt 10b | wnt10b | 80%* | indicator of development, cell fate decisions | [43] |
| wnt 4 | wnt4 | 65%* | indicator of development, synaptic specificity | [44] |
| frizzled | frizzled | 81%* | indicator of development, wnt receptor | [45] |
| adenomatous polyposis coli | apc | 83%* | indicator of development, involved in optic lobe development | [46] |
| axin | axin | 73%* | indicator of development, wnt signalling inhibition | [47] |
| RAR related orphan receptors | ror | 66% | indicator of development, wnt correceptor | [48] |
| cyclin dependent kinase 2 | CDK2 | 81%* | phosphorylation of Foxo | [49] |
| serotonin transporter | sert | 91%* | serotonin is involved in the regulation of PTTH release/snythesis | [50] |
| dopamine receptor isoform 1 | D1r | 92%* | dopamine can induce diapause | [51] |
| melatonin receptor | mtnr | 43%* | triggers PTTH release | [52] |
| dopamine receptor isoform 2 | D2r | 90%* | dopamine can induce diapause | [51] |
| sodium dependent dopamine transporter | Sddt | 90%* | dopamine can induce diapause | [51] |
| ras | ras | 91%* | downstream of PTTH signaling | [14] |
| ecdysone inducible gene E74 | E74° | 95% | induced by ecdysone signalling, puparium formation, involved in metamorphosis | [24, 53] |
| ecdysone inducible gene E75 | E75 | 77% | involved in adult head eversion and leg morphogenesis | [24, 54] |
| ecdysone inducible gene E75B | E75B | 94% | involved in adult head eversion and leg morphogenesis | [24, 54] |
| drosophila hormone receptor 3 | DHR3 | 68% | differentiation of adult structures during metamorphosis | [24, 55] |
| drosophila hormone receptor 4 | DHR4 | 64% | controls the timing of steroid hormone pulses during development | [24, 56] |
| ecdysone inducible gene E78 | E78 | 96% | control of early embryogenesis | [24, 57] |
| ftz transcription factor 1 | FTZ-F1 | 76% | regulator of insect metamorphosis | [24, 58] |
| npc intracellular cholesterol transporter | npc1 | 55% | spermatogenesis, trafficking of sterols | [59, 60] |
| ecdysone inducible gene E93 | E93 | 34% | specification of adult stage, involved in adult differentiation of pupa | [61] |
| protein kinase C | PKC | 76% | downstream of PTTH signaling, involved in signal transduction cascades | [14, 38] |
| eukaryotic translation initiation factor 4E-binding protein | 4E-BP | 49% | regulator of overall translation levels | [62] |
| Protein tyrosine phosphatase 61F | PTP61F | 48% | negative regulation of MAP kinase signaling | [63] |
| phosphoinositide 3 kinase | Pi3K | 75%* | insulin signaling pathway, phosphorylation of akt | [27] |
| insulin like peptide 2a | ILP2a | 41%* | insulin signaling pathway | [27] |
| insulin like peptide 5 | ILP5 | 25% | insulin signaling pathway | [27] |
| mothers against decapentaplegic homolog 1 | smad1 | 93%* | requierd in wnt signaling | [64] |
| glycogen synthase kinase 3 beta | gsk3b | 89%* | promotes synaptogensis in neurons | [65] |
| juvenile hormone epoxide hydrolase | JHEH | 62%* | degradation of juvenile hormone | [66] |
| juvenile hormone diol kinase | JHDK | 53%* | inactivation of juvenile hormone | [67] |
| myocyte enhancer factor -2 | mef2 | 88%* | regulation of PTTH expression | [68] |
| start | start | 50% | cholesterol traffic and steroid synthesis | [69] |
|  |  |  |  |  |

^a^ Percent identity with the protein sequence in Drosophila melanogaster, ° Manduca sexta, and

* Bombyx mori.

**S2.1: Pair wise degrees** of Insulin like peptide sequence identity (upper number), similarity (middle number) and gaps (lower number) based on a multiple sequence alignment in Figure 2.

|  | Hsap_Ins | Dmel_ILP2 | Dmel_ILP5 | Pnap_ILP1a | Pnap_ILP1b | Pnap_ILP2a | Pnap_ILP2b | Pnap_ILP2c | Pnap_ILP3 | Pnap_ILP4 | Pnap_ILP5 | Pnap_ILP6 |
| --- | --- | --- | --- | --- | --- | --- | --- | --- | --- | --- | --- | --- |
|  |  |  |  |  |  |  |  |  |  |  |  |  |
| Hsap_Ins | 51 | 31% | 24% | 26% | 28% | 25% | 24% | 24% | 21% | 20% | 27% | 30% |
|  | 0 | 39% | 37% | 40% | 40% | 45% | 43% | 43% | 36% | 35% | 36% | 44% |
|  | 0 | 18% | 20% | 25% | 25% | 14% | 19% | 19% | 26% | 31% | 18% | 13% |
|  |  |  |  |  |  |  |  |  |  |  |  |  |
| Dmel_ILP2 | 18 | 54 | 25% | 23% | 25% | 30% | 27% | 27% | 21% | 16% | 19% | 35% |
|  | 23 | 0 | 37% | 32% | 32% | 42% | 44% | 43% | 37% | 25% | 27% | 50% |
|  | 11 | 0 | 18% | 29% | 29% | 12% | 17% | 17% | 21% | 43% | 31% | 11% |
|  |  |  |  |  |  |  |  |  |  |  |  |  |
| Dmel_ILP5 | 14 | 15 | 53 | 30% | 32% | 26% | 24% | 26% | 17% | 26% | 19% | 20% |
|  | 22 | 22 | 0 | 35% | 33% | 40% | 38% | 40% | 28% | 33% | 31% | 33% |
|  | 12 | 11 | 0 | 24% | 24% | 17% | 15% | 15% | 23% | 30% | 32% | 22% |
|  |  |  |  |  |  |  |  |  |  |  |  |  |
| Pnap_ILP1a | 14 | 13 | 16 | 40 | 87% | 34% | 33% | 33% | 21% | 42% | 32% | 21% |
|  | 21 | 18 | 19 | 0 | 95% | 50% | 48% | 46% | 31% | 50% | 48% | 33% |
|  | 13 | 16 | 13 | 0 | 0% | 25% | 29% | 29% | 42% | 29% | 22% | 35% |
|  |  |  |  |  |  |  |  |  |  |  |  |  |
| Pnap_ILP1b | 15 | 14 | 17 | 35 | 40 | 34% | 33% | 33% | 21% | 42% | 30% | 23% |
|  | 21 | 18 | 18 | 38 | 0 | 48% | 44% | 42% | 31% | 49% | 46% | 33% |
|  | 13 | 16 | 13 | 0 | 0 | 25% | 29% | 29% | 42% | 29% | 22% | 35% |
|  |  |  |  |  |  |  |  |  |  |  |  |  |
| Pnap_ILP2a | 14 | 17 | 15 | 18 | 18 | 51 | 71% | 69% | 21% | 28% | 25% | 33% |
|  | 25 | 24 | 23 | 26 | 25 | 0 | 84% | 83% | 46% | 41% | 41% | 49% |
|  | 8 | 7 | 10 | 13 | 13 | 0 | 5% | 5% | 26% | 38% | 21% | 13% |
|  |  |  |  |  |  |  |  |  |  |  |  |  |
| Pnap_ILP2b | 14 | 16 | 14 | 18 | 18 | 38 | 52 | 96% | 20% | 25% | 24% | 32% |
|  | 25 | 26 | 22 | 26 | 24 | 45 | 0 | 96% | 39% | 41% | 36% | 42% |
|  | 11 | 10 | 9 | 16 | 16 | 3 | 0 | 0% | 24% | 37% | 25% | 18% |
|  |  |  |  |  |  |  |  |  |  |  |  |  |
| Pnap_ILP2c | 14 | 16 | 15 | 18 | 18 | 37 | 50 | 52 | 18% | 26% | 24% | 32% |
|  | 25 | 25 | 23 | 25 | 23 | 44 | 50 | 0 | 37% | 41% | 34% | 42% |
|  | 11 | 10 | 9 | 16 | 16 | 3 | 0 | 0 | 24% | 37% | 25% | 18% |
|  |  |  |  |  |  |  |  |  |  |  |  |  |
| Pnap_ILP3 | 15 | 15 | 12 | 15 | 15 | 15 | 14 | 13 | 69 | 18% | 33% | 24% |
|  | 25 | 26 | 20 | 22 | 22 | 32 | 27 | 26 | 0 | 31% | 40% | 39% |
|  | 18 | 15 | 16 | 29 | 29 | 18 | 17 | 17 | 0 | 29% | 28% | 14% |
|  |  |  |  |  |  |  |  |  |  |  |  |  |
| Pnap_ILP4 | 13 | 12 | 17 | 24 | 24 | 19 | 17 | 18 | 14 | 57 | 26% | 21% |
|  | 23 | 18 | 22 | 29 | 28 | 28 | 28 | 28 | 23 | 0 | 40% | 28% |
|  | 20 | 31 | 20 | 17 | 17 | 26 | 25 | 25 | 22 | 0 | 23% | 36% |
|  |  |  |  |  |  |  |  |  |  |  |  |  |
| Pnap_ILP5 | 15 | 12 | 12 | 16 | 15 | 14 | 14 | 14 | 23 | 16 | 49 | 27% |
|  | 20 | 17 | 19 | 24 | 23 | 23 | 21 | 20 | 28 | 24 | 0 | 37% |
|  | 10 | 19 | 20 | 11 | 11 | 12 | 15 | 15 | 20 | 14 | 0 | 22% |
|  |  |  |  |  |  |  |  |  |  |  |  |  |
| Pnap_ILP6 | 18 | 21 | 13 | 13 | 14 | 20 | 20 | 20 | 17 | 15 | 17 | 59 |
|  | 26 | 30 | 21 | 20 | 20 | 29 | 26 | 26 | 27 | 20 | 23 | 0 |
|  | 8 | 7 | 14 | 21 | 21 | 8 | 11 | 11 | 10 | 26 | 14 | 0 |

**S2.2: Complete insulin amino acid sequences** with A-chain (yellow), B-chain (green), C-chain (cyan), proteolytic cleavage site (red) and conserved cysteine residues (bold) indicated.

Hsap_Ins

MALWMRLLPLLALLALWGPDPAAAFVNQHL**C**GSHLVEALYLV**C**GERGFFYTPKTRREAEDLQVGQVELGGGPGAGSLQPLALEGSLQKRGIVEQ**CC**TSI**C**SLYQLENY**C**N

Dmel_ILP2

MSKPLSFISMVAVILLASSTVKLAQGTL**C**SEKLNEVLSMV**C**EEYNPVIPHKRAMPGADSDLDALNPLQFVQEFEEEDNSISEPLRSALFPGSYLGGVLNSLAEVRRRTRQRQGIVER**CC**KKS**C**DMKALREY**C**SVVRN

Dmel_ILP5

MMFRSVIPVLLFLIPLLLSAQAANSLRA**C**GPALMDMLRVA**C**PNGFNSMFAKRGTLGLFDYEDHLADLDSSESHHMNSLSSIRRDFRGVVDS**CC**RKS**C**SFSTLRAY**C**DS

Pnap_ILP1a

MKVLVLLVAISMASAAEAQGVY**C**GRRLAMALAML**C**DTNEDKRGDWSMYGPLAHERTEHQDNWSVWPSLPQPWLQLSRARSLGRPKRQVVME**CC**MKP**C**SEDELMAY**C**-

Pnap_ILP1b

MKVLVLLVVLSVAIAAEAQGFY**C**GRRLAIALAML**C**EGSEDKRADLSVYGSMGHERAGHQGDWSAWPSVSQHWLHANRAHSLGRPKRQVVME**CC**MKP**C**SEDELMAY**C**-

Pnap_ILP2a

MKYQAVLIFALVLCSSQGQGRKL**C**GRKLSDILGYL**C**ANPLTSKEDLDSFQMKRSENSYNSITNAVDWPWIPHHKAKGIRNKREIIEE**CC**DKS**C**TIDELMEY**C**-

Pnap_ILP2b

MKYQAVLIFALVLCSSQGEGRML**C**GGRLSEIVAYL**C**ANPLTSREDFASFQMKRAGNSYNSITNAVDWPWIPQNEAKGIRNKRRIIDE**CC**KKS**C**TVDELMEY**C**-

Pnap_ILP2c

MKYQAVLIFTLVLCISQGESRML**C**GGRLPEIVAYL**C**ANPLTSREDFASFQMKRAETSYNSITNAVDWPWIPHHEAKGIRNKRRIIDE**CC**KKS**C**TVDELMEY**C**-

Pnap_ILP3

MSLIAKNFHWLCIVFLYGNIISGDSLTLNSMLKEL**C**SRSLSNLIFHV**C**NGDIMINDFPELDQPKVRSRRAALIYASMRIKRQLVDE**CC**LHP**C**SIAQLVQY**C**PTEEW-

Pnap_ILP4

MHPSSVVFIAALISECYGHIGGGGVSLQEASSRMY**C**GRTLARTLAFL**C**YDAPSSHKRSESGSMYNAILSPYYKDQATQVDWPWMTTQTAKALGLSARGKRDFVVSE**CC**DKA**C**SINELLSY**C**-

Pnap_ILP5

MKLAVIVLCVMTMSRADQETPVVL**C**GRELANARVLV**C**YGAEFVSKRASPQSIVAALASGDKWIEELMWGGRRAAISADWNRYKRGGLADE**CC**LKP**C**TTADILNY**C**-

Pnap_ILP6

MSVVKSAHLAVITVFLLVDTCYSAPPAFKL**C**GRQLVETMEGL**C**KEYNSPPWDVPTGKKFLIEQPTGAVRRKRQMGIADR**CC**VSS**C**RAAELLQY**C**SVIK-

underlined: signal peptide sequence

green: B-chain

yellow: A-chain

cyan: C-peptide

red: proteolytic cleavage sites

**bold**: conserved cysteine residues

**S2.3 Localization of insulin like peptide genes in *Pieris napi*.**

***Pieris napi* chromosome 16**

**Pnap_ILP1a gene:** Position 481576 - 481896 rev+comp

**atg**aaagtactagttttgttggtggcaatcagcatggcgagcgcagctgaagcacaaggtgtttactgtgggaggaggttggctatggcgctggcaatgctgtgtgatacaaacgaggacaaacgaggggactggtctatgtatggaccattggcgcacgagagaactgagcaccaagataactggtccgtctggccgtcgttacctcaaccctggctgcagctaagccgggctcgcagcctgggacgccccaaaagacaggtcgtgatggagtgctgcatgaaaccctgctcggaagacgaacttatggcctactgc**taa**

cDNA-sequence:

**atg**aaagtactagttttgttggtggcaatcagcatggcgagcgcagctgaagcacaaggtgtttactgtgggaggaggttggctatggcgctggcaatgctgtgtgatacaaacgaggacaaacgaggggactggtctatgtatggaccattggcgcacgagagaactgagcaccaagataactggtccgtctggccgtcgttacctcaaccctggctgcagctaagccgggctcgcagcctgggacgccccaaaagacaggtcgtgatggagtgctgcatgaaaccctgctcggaagacgaacttatggcctactgc**taa**

MKVLVLLVAISMASAAEAQGVY**C**GRRLAMALAML**C**DTNEDKRGDWSMYGPLAHERTEHQDNWSVWPSLPQPWLQLSRARSLGRPKRQVVME**CC**MKP**C**SEDELMAY**C**-

**Pnap_ILP1b gene:** Position 451738 - 452058

**atg**aaggtactagttctgttggttgtactgagcgtagcaatcgcggcagaggcccaaggtttctactgcgggaggcggttggctatagctctggctatgctgtgtgagggaagcgaagacaagcgtgccgacttgtcagtctatggatctatggggcacgagagagctggccatcaaggggactggtccgcctggccttctgtgtctcaacactggctgcacgcaaatcgggcgcacagccttggacgccccaaaagacaggtcgtgatggagtgctgcatgaaaccctgctcggaagacgaacttatggcctactgc**taa**

cDNA-sequence:

**atg**aaggtactagttctgttggttgtactgagcgtagcaatcgcggcagaggcccaaggtttctactgcgggaggcggttggctatagctctggctatgctgtgtgagggaagcgaagacaagcgtgccgacttgtcagtctatggatctatggggcacgagagagctggccatcaaggggactggtccgcctggccttctgtgtctcaacactggctgcacgcaaatcgggcgcacagccttggacgccccaaaagacaggtcgtgatggagtgctgcatgaaaccctgctcggaagacgaacttatggcctactgc**taa**

MKVLVLLVVLSVAIAAEAQGFY**C**GRRLAIALAML**C**EGSEDKRADLSVYGSMGHERAGHQGDWSAWPSVSQHWLHANRAHSLGRPKRQVVME**CC**MKP**C**SEDELMAY**C**-

***Pieris napi* chromosome 4**

**Pnap_ILP2a gene:** Position 7367793 - 7368222

**atg**aagtaccaggccgtcctcatctttgctctcgtcttatgtagcagtcaagggcaaggccgaaagttgtgcggacgaaaactgtccgacatcttagggtatttatgcgcaaacccgttgacaagtaaagaagatttggaca*gt*aagttttattcttaaagtttctttggtgaaccgataaaacttttaatgataacttatgcctcaactttaatcttccagtttttaattgctagaaacaaacttaaatatattgttttc*ag*gttttcaaatgaaacgatccgagaactcttacaactcaataaccaatgctgtcgactggccctggatcccacatcataaagccaaggggattaggaacaagcgtgaaatcatcgaggagtgttgcgataagtcctgcaccattgacgaactcatggaatattgt**taa**

cDNA-sequence:

**atg**aagtaccaggccgtcctcatctttgctctcgtcttatgtagcagtcaagggcaaggccgaaagttgtgcggacgaaaactgtccgacatcttagggtatttatgcgcaaacccgttgacaagtaaagaagatttggacagttttcaaatgaaacgatccgagaactcttacaactcaataaccaatgctgtcgactggccctggatcccacatcataaagccaaggggattaggaacaagcgtgaaatcatcgaggagtgttgcgataagtcctgcaccattgacgaactcatggaatattgt**taa**

MKYQAVLIFALVLCSSQGQGRKL**C**GRKLSDILGYL**C**ANPLTSKEDLDSFQMKRSENSYNSITNAVDWPWIPHHKAKGIRNKREIIEE**CC**DKS**C**TIDELMEY**C**-

**Pnap_ILP2b gene:** Position 7359672 - 7360085

**atg**aagtaccaggccgtcctcatctttgctctcgtcttatgcagcagtcaaggggaagggcgaatgttatgcggaggaagactgtccgaaatcgtagcgtatttatgcgcaaacccgttgacgagtagagaagatttcgcca*gt*aagttcatactcgttaagagttttattcttaatctttctttggtgagccgatgataactttaatttgataatgtgcgtgaatagtaaattttattgttttc*ag*gttttcaaatgaaacgagcggggaactcctacaactcaataaccaatgctgtcgactggccctggataccacaaaatgaagctaaggggattaggaacaagcgtagaatcatcgacgagtgttgcaaaaagtcctgtactgttgacgaactcatggaatattgt**taa**

cDNA-sequence:

**atg**aagtaccaggccgtcctcatctttgctctcgtcttatgcagcagtcaaggggaagggcgaatgttatgcggaggaagactgtccgaaatcgtagcgtatttatgcgcaaacccgttgacgagtagagaagatttcgccagttttcaaatgaaacgagcggggaactcctacaactcaataaccaatgctgtcgactggccctggataccacaaaatgaagctaaggggattaggaacaagcgtagaatcatcgacgagtgttgcaaaaagtcctgtactgttgacgaactcatggaatattgt**taa**

MKYQAVLIFALVLCSSQGEGRML**C**GGRLSEIVAYL**C**ANPLTSREDFASFQMKRAGNSYNSITNAVDWPWIPQNEAKGIRNKRRIIDE**CC**KKS**C**TVDELMEY**C**-

**Pnap_ILP2c gene:** Position 7364063 - 7364453

**atg**aagtaccaggccgtcctcatctttactctcgtcttatgcatcagtcaaggggaaagccgaatgttatgcggtggtagactgcccgaaatcgtagcgtatttatgcgcaaatccgttgacgagtagagaagatttcgcca*gt*aagttcttactcgctaagagttttattcttaaactttctttggtgagacgataaaacatatttaattatattgttttc*ag*gttttcaaatgaaacgagccgagacctcctacaactcaataaccaatgctgtcgactggccctggatcccacatcatgaagctaaggggattaggaacaagcgtagaatcatcgacgagtgttgcaaaaagtcctgtacagttgacgaactcatggaatattgt**taa**

cDNA-Sequenz:

**atg**aagtaccaggccgtcctcatctttactctcgtcttatgcatcagtcaaggggaaagccgaatgttatgcggtggtagactgcccgaaatcgtagcgtatttatgcgcaaatccgttgacgagtagagaagatttcgccagttttcaaatgaaacgagccgagacctcctacaactcaataaccaatgctgtcgactggccctggatcccacatcatgaagctaaggggattaggaacaagcgtagaatcatcgacgagtgttgcaaaaagtcctgtacagttgacgaactcatggaatattgt**taa**

MKYQAVLIFTLVLCISQGESRML**C**GGRLPEIVAYL**C**ANPLTSREDFASFQMKRAETSYNSITNAVDWPWIPHHEAKGIRNKRRIIDE**CC**KKS**C**TVDELMEY**C**-

**Pnap_ILP3 gene:** Position 4824910 - 4825230 rev+comp

**atg**tccctaatagcgaagaattttcattggttgtgcatcgtattcttgtatgggaacataataagtggagattccttgacattaaactcgatgttgaaagaattgtgcagtcgatcgctgtcgaacctcatattccacgtttgtaacggtgatataatgatcaacgatttcccggaattggatcagccaaaagttagatcgcggcgtgctgcactaatttacgcgtcgatgagaatcaagcgacaactcgtagatgagtgctgccttcatccatgctctatagcccagttggttcaatactgccctactgaagaatgg**tga**

cDNA-sequence:

**atg**tccctaatagcgaagaattttcattggttgtgcatcgtattcttgtatgggaacataataagtggagattccttgacattaaactcgatgttgaaagaattgtgcagtcgatcgctgtcgaacctcatattccacgtttgtaacggtgatataatgatcaacgatttcccggaattggatcagccaaaagttagatcgcggcgtgctgcactaatttacgcgtcgatgagaatcaagcgacaactcgtagatgagtgctgccttcatccatgctctatagcccagttggttcaatactgccctactgaagaatgg**tga**

MSLIAKNFHWLCIVFLYGNIISGDSLTLNSMLKEL**C**SRSLSNLIFHV**C**NGDIMINDFPELDQPKVRSRRAALIYASMRIKRQLVDE**CC**LHP**C**SIAQLVQY**C**PTEEW-

***Pieris napi* chromosome 19**

**Pnap_ILP4 gene:** Position 3908268 - 3909357 rev+comp

**atg**cacccttcaagcgtggttttcattgcggcactaatctcagagtgctatggtcatattggtggaggaggcgttagtcttcaagaggccagttctcggatgtactgcggacgtactctagcaagaacactagctttcctctgctacgatgcaccatccagccataaaagatcggaaagcggatctatgtata*gt*aagtggccgtaatgtacaaagatcgcttaacttcatgtactaaaatcccaatggatacccaaaaaacgcagacatcgcagcccatagatactcaattttagtgggttagttgcctttaagggaggagtaaacaattggaggtcgtatctctccgggaagattttatggcctggtaacgagacctttaagccaagtcaaattttcgggagaagtaaatgatatccgccgcccatagacacctgcagactcactactacttcttgctgttttttatgttcgtaatgtttcataattgcagccaaatttagtaaataacttttcattattcggttagttatttattgctaaatcatatggtaggctgatttgaggagtcttagggcgccttcaaatttgccgcaacgtgccgctcggcggcagcgctgcagacccactgctgtcgctaagtttcagaatcgtagtgtagtatatattttcacgatggactctaacgaagaaatactattcttagtattactattaaaactaaaaaggattcattttgttagcccagaaggagaattgtcatggcaagtaatgaaaatataaaatagttgtgttaaaaatgtacttattaggatagactctaagtcgggactagactagtagactcccatcaaaatcctcaacgtttttggcccttagtgtatagatgaaagtgaaattttgaatactgttttc*ag*atgcaatcttatctccatactacaaggaccaggctacacaagtcgattggccctggatgactacgcagacagccaaggctcttggcctatctgctcgtggcaaaagggatttcgtcgttagtgaatgttgtgataaagcttgcagtatcaatgaattgttgtcctactgc**taa**

cDNA-sequence:

**atg**cacccttcaagcgtggttttcattgcggcactaatctcagagtgctatggtcatattggtggaggaggcgttagtcttcaagaggccagttctcggatgtactgcggacgtactctagcaagaacactagctttcctctgctacgatgcaccatccagccataaaagatcggaaagcggatctatgtataatgcaatcttatctccatactacaaggaccaggctacacaagtcgattggccctggatgactacgcagacagccaaggctcttggcctatctgctcgtggcaaaagggatttcgtcgttagtgaatgttgtgataaagcttgcagtatcaatgaattgttgtcctactgc**taa**

MHPSSVVFIAALISECYGHIGGGGVSLQEASSRMY**C**GRTLARTLAFL**C**YDAPSSHKRSESGSMYNAILSPYYKDQATQVDWPWMTTQTAKALGLSARGKRDFVVSE**CC**DKA**C**SINELLSY**C**-

***Pieris napi* chromosome 3**

**Pnap_ILP5 gene:** Position 5945346 - 5945846

**atg**aagcttgcagtgatagttttatgtgtgatgacgatgtcacgagccgatcaggaaacacctgtggttctatgtggaagggaacttgccaatgccagagtgttagtttgctatggcgctgagtttgtttctaaacgagcgtcaccgcaatctatagtcg*gt*tagtacattttattatataccttcatgtttattttttatttctttaaaaggctgtttagaaaataaatggaatcatgttaactttaaactctggctaaattaacaaatctcgccattttaatctaccttcgatatacttgcatctatcaaatatattgtaattaatcaactgattttac*ag*cggcattagcaagtggcgataaatggattgaagaactcatgtggggagggcgtcgtgccgctataagtgctgattggaaccgatacaaaaggggcgggttggctgatgaatgctgtcttaaaccctgcaccaccgctgatatacttaactactgc**taa**

cDNA-sequence:

**atg**aagcttgcagtgatagttttatgtgtgatgacgatgtcacgagccgatcaggaaacacctgtggttctatgtggaagggaacttgccaatgccagagtgttagtttgctatggcgctgagtttgtttctaaacgagcgtcaccgcaatctatagtcgcggcattagcaagtggcgataaatggattgaagaactcatgtggggagggcgtcgtgccgctataagtgctgattggaaccgatacaaaaggggcgggttggctgatgaatgctgtcttaaaccctgcaccaccgctgatatacttaactactgc**taa**

MKLAVIVLCVMTMSRADQETPVVL**C**GRELANARVLV**C**YGAEFVSKRASPQSIVAALASGDKWIEELMWGGRRAAISADWNRYKRGGLADE**CC**LKP**C**TTADILNY**C**-

***Pieris napi* chromosome 23**

**Pnap_ILP6 gene:** Position 7088012 - 7091162 rev+comp

**atg**tccgtggtaaagtcggcacatttggccgtgatcacagtattcttgctggtggatacatgttattcagcgccacccgctttcaaattg**tgc**ggcagacaacttgtggagaccatggaaggtcta**tgc**aaagagtacaatagccctccttgggatgtacctacaggtaagaaatttt*gt*caatatatcttttatactgtgtgtcgatgtgctaatttttatgacgagaggtaacagaaaaagagtaataagaattacgttatttaatgtgttctaaggtgatcttatgagtaagagaatgagattcagtcgaaactaccacaaaattaaacatatacaaaatacaaataattaatattggttcaaaatttggttcgcaataaattttactttgtcaactacagatttttcctaaataaaaattacatctaattttattatttaaaaaaaggtctgccggggattactattactaaatctagaaacaaacacaatatgctttgtacttcataactataaataacaaaatactattaacaaaaaacaatattgtaaaatttcaacagtgaaaactgtatttgaaagttacgtgatgtatctcgtagcaggctctttgggtaaactaaaacttagctaagtatgacttcaatatgtcaaaaactatcagattttgtcatacgggagttacagtttgcaagactctgaagggtcaccactttgaaccaatccaataacaatcgtagcaactttttttaagaggggaaatttttggtagtgtgggactgagccggaagacggatacctgaactaatattcctttaccccatctatttatttatttattacagaaatacattatccttacagactatgccatacgataatgtcgataaaaataaaatataataatgtacgtaataatattaaatacataatatacacaatatcgctactgtgaattctgaattctgagttcactctgcgaacatataaatatgttgatagtgtcggagacagcattcagactggttcttgcccttggtaaatcgcatacatccagtatagtagccacaagcaaagccttatgccttgcgatcgaggtcgaagcccctgagggtgcacctctctctaaccgcaaaccggtagtcggtaaaactatggcaaatcgcttaaaaaaattacggtatatcaagcagaacaaattgaatactgttatttcaaatgaacaagtactttaatgttttcgacaaatgcttttagcatttttacaaatactggtgatattcaaaatctagaaacagtgctaagtttactttcatatgtcggaaacgtattgaattttgacagacagggtatttagtcaagattagtagtatatatgagtaggagtcgaaaactaaatttgtatgaaactttatcgacacaaactgtcatttacgcttgtctgtcatgttctgtcactatgttcgcgctaagtctcgttacttaatgtcaccctaaaatttaccctttttggttatattttattcgtcctattcgggaaaactagtacccttttttttatggctctggcacggtttgtgcattagccagcgtcaagtataagatttttataattcgtgctttttgccttagaaattcgaccatgtcctccatgtacggtttatccattcgcccggtaccgcacaaccctcccaaaggccgaaaacaaatttaaattaaattaaaacttgccctcgaaccgggaatcgaacccggtacccctcacctagctgccacttaataagaccgctaggctatgaggcccctctagtactctttaaagagagcaaaattacgaagagaatgttagcaagagccaattatccgcttggtaacagtatctcttatcattacgcaaaagataaggttatgagttcaataagggcagggctttctagacatgagaatagatgtaaacattaacacattgcattgtctgcgactttgacgtgagactgattggtataaaaataaacgatatcaataaagtattttaattacctaataatctatcttatatatagtctgtactgcagttataaatagcattgataactgttattatttgtttattgacactatttttgattaaggaaataaaaaaaacatggcaactcatttaaagttccagagtcaaccgaacttcttttgtttattaccaaattattttcgcttgttgatctgtgaactggaccacaaatatttatatttagatccgtattggtaaagaattaattacttaacatgaaggcaacgtgtgtttaataaaaattatataattatttatattagtttttatctcttaatcgcgacctcttgattgtttacaacttaatataggaaaaacaagcttaacgcctccctattaatattattatcaatcacgaaacagtttcgtgctatgttatataaaccaaggacatcttgggagcgtaacatcgtgggaatttccattcagcataaaacaataaaaaacacttgtgtctactgtattcgcacacaatggccattcgaaattcaaaattgaccatagctattagaatgggaaaagtcataatttaaattgtgcaattaagtatggcaagtctgaaaggcgccgatttaatgtgaatcataatatcagaatcagagattcaatgggtgttgaagtcaacagttctaatataaaaaataatttaatgtgtataataatgaataaaactgtaggtaccctaattttatataaaaagggacttcctttaagtaagataatagtctgaaataagaccatccgataaaaattaattcctcatatatccaaagttatccgttactaggcgaggcgatgattcgaagccaacaaaaacgataataaaataatcggtttacatatcacttttcaagcgtcatcttaccacaggctcaaaacgtattgtaccaagaagaaacgtcaaaatcgagtgttaaatacagacaacaaagacgttaacttattaaaaaaatattttc*ag*taattgaacaacctacaggcgcagtcagacggaaaaggcaaatgggtatagcggatcga**tgttgc**gtctcgtct**tgt**cgggcagctgagcttctacaatac**tgc**tccgtaataaag**taa**

cDNA-sequence:

**atg**tccgtggtaaagtcggcacatttggccgtgatcacagtattcttgctggtggatacatgttattcagcgccacccgctttcaaattgtgcggcagacaacttgtggagaccatggaaggtctatgcaaagagtacaatagccctccttgggatgtacctacaggtaagaaatttttaattgaacaacctacaggcgcagtcagacggaaaaggcaaatgggtatagcggatcgatgttgcgtctcgtcttgtcgggcagctgagcttctacaatactgctccgtaataaag**taa**

MSVVKSAHLAVITVFLLVDTCYSAPPAFKL**C**GRQLVETMEGL**C**KEYNSPPWDVPTGKKFLIEQPTGAVRRKRQMGIADR**CC**VSS**C**RAAELLQY**C**SVIK-

**S3.1:** **Multiple Sequence Alignment of Torso.** *A black background indicates fully conserved residues; a gray background indicates partially conserved residues.*

Dros : MLIFYAKYAFIFWFFVGSNQGEMLLMDKISHDKTLLNVTACTQNCLEKGQMDFRS---CLKDCRINGTFPGALRKVQENYQMNMICRTES : 87
Bomb : -MYSEGKLLKVFLIFAGFIIFS-LCGEVVSQRYPPAPGLLKYLEQDVCYSLYYYLNWTSLADCKTN-FEETG--ISDVPSTVKVRCQSKN : 85
Pnap : ---MHLKINEWLLIIKTFAFLPGLFADTSFTSLPLTEEQLKGLANDVCHDIFYEP---KAAACTNK-FFGTRYETKTEPPSVQVKCRAEQ : 83
Prap : ---MHLKINEWLLIIKTCAFLPGLFANTLFNNLPLTEEQIEELANDVCHDIFYEP---KATACTNK-FFGNRYETNTEPPSVQVKCRAEQ : 83
Pbra : -----MHLYAWLLIMKTCAFLPGLFADTLLTDLPLTEEQLKRLACDVCSDMFESE---GVDKCVNI-FLGTHYEKDTEPPSVQVKCRSEQ : 81


Dros : EIVFQIDWVQHSRGTEPAPNATYIIRVDAVKDDNKETALYLSDDNFLILPGLESNSTHNITALAMHGDGSYSLIAKDQTFATLIRGYQPS : 177
Bomb : SIRFETEPSEHWQLFILM--EHDNFDPIPFTLIEPNNVFGE-----LITTANKEYQIWSTYLDEYGTLQDWMEGPIVLKFDQRNQQPDDI : 168
Pnap : KLTFAVPQSKDWQLVVLIPEEVDMMEYFIFNAMEPAREIGS-----IYTPP-GAFTLWSALLNDTGSPSIWKKSPILEAWNDTVADVEER : 167
Prap : KLTFAVPQSKDWQLVVLIPEEVDMMEYFIFNAMEPAREIGS-----IYTPP-GPFTLWSALVNDLGSPSVWKKSSILEAWNETVADNQER : 167
Pbra : KLTFAVPRSNDWQLVVLIPEEVDMMDYLIFNVMEPQTEIGS-----IYTPP-GAFTLWSALVNDSGSPSNWKNS-SIEDWNDIAADIEER : 164


Dros : KMGAVNLLRFVPQPDDLHHIAAEIEWKPSAESNCYFDMVSYSTNSVNMDEPLEVQFRDRKKLYRHTVDNLEFDKQYHVGVRTVNIMNRLE : 267
Bomb : KYNVTQEFKYIILGNDSYTINGKFVWNTTGDRDLCFDIANICQN---TNMKHAKIWPTAHPSFD--VENLVLNDECEIHVKGIHGTTKHK : 253
Pnap : NYVVNYTFLFDKFDNATADLDVKLTWNATDPSDSCFEVYNRCNSKTLGNVLHRSIGPDKNKSI---IATVPLDDKCTILVKGKYGTTKFQ : 254
Prap : NYEVNYTFLFDNFDNTTADLDVKLTWNATDPSDSCFEVYNRCNSKTLGNVLHRSIGPDKSKSL---IATVPLDDKCTILVKGKYGTTKFE : 254
Pbra : NYVVNYTFLFDNFDNTTADIDVKLTWNTTDPSDVCFEVYNRCNSNTLGNVLHSSIGADKNNSL---IARVPLDDKCSILVKGKYGTTKFQ : 251
 y v f 5 1 6 k Wn 3 s1 cF 6 n c 1 h i d s 6 6 l1d c 6 V4g gtt4

 280 * 300 * 320 * 340 * 360
Dros : SDLQWLPIAVPSCLDWYPYNYTLCPPHKPENLTVTQKQYLPNILALNITWARPRYLPDNYTLHIFDLFKGGTELNYTLDQNRSHFYVPKI : 357
Bomb : -------YKTPSCFELPECFLNNMEPEIPQDVAIAADQDLRGWWNINVAWAKPHFQPEIYNVTVRANMIRSIILPGNATETTFRNIPNTF : 336
Pnap : -------YQTPSCYDLPGCKY---LPEKPENVKLTAKENGD-SWLVSVKWRQPKHPPSYYNVTLRADKVFTIKASNLSSEAKFTNVT--- : 330
Prap : -------YQTPSCYDLPGCKY---LPEKPENVTLTAKRNGD-SWLVSVKWKPPKFPPAYYNVTLRADKVYTINASYLSSEANFMNVT--- : 330
Pbra : -------YQTPSCYDLPGCKY---LPEKPENVTLTAKRNGD-SWLVSVKWRPPKFPPSYYNVTLRADKVYTIKASYLSSVANFTNVT--- : 327


Dros : TVLGSHFEVHLVAQSAGGKNVSGLTLDKVHRGVLLSEGNMVKLVLFIIVPICCILMLCSLTFCRRNRSEVQALQMDAKDAKASEFHLSLM : 447
Bomb : LSAGKIYNVSVYAIIGQK---ASHTSRRAFTPGMLRWVWAGATAGAG---CAAGGLLAATLLCCGHRRATSRVSQEDP--DEKTPKEDDV : 418
Pnap : --GRGYYNVTVDAINDIG---HAVTFQRSIFPLVEESASVSLLIGAWAEVIVISTIIATFFVWWKRQRDIKKRNMYFPGVREKVLKDGEL : 415
Prap : --GRGYYNVTVDAINKIG---HALTFRRSIFPPVEENASLSLLIGAWAELIVISTIIATFSVWWKRRRDTKKRNMYFPGVREKGLKDGEL : 415
Pbra : --GRGYYNVTVDAINKIG---HALTFRRNIFPPVEESASVSLLIGAWAEVIVISAIIATFFVWWKRQRDIKKRNMYFPGVREKVLRDAEL : 412


Dros : DSSGLLVTLSANESLEVMDELEVEPHSVLLQDVLGEGAFGLVRRGVYK--KRQVAVKLLKDEPNDEDVYAFKCEIQMLKAVGKHPNIVGI : 535
Bomb : EIIGIESGS-------ADDHWEVRSDRVLLHEVIGEGAFGVVRRGTLAPGGKSVAVKMLKEFPSQEEVRSFRSEMELMKSVGAHPHVVSL : 501
Pnap : EICCVESG--------SEEQWEVKPERLLLHEVIGEGAFGVVRRATLSPDDKIVAVKMLKDFPSVEEIRSFRAEMELMKSVGSHPHVVSL : 497
Prap : EICCVESG--------SEEQWEVKPERLLLHEVIGEGAFGVVRRGTLSPEDKIVAVKMLKDFPSVEEIRSFRAEMELMKSVGSHPHVVSL : 497
Pbra : EICCVESG--------SEEQWEVKPERLLLHEVIGEGAFGVVRRGTLSPDDKIVAVKMLKDFPSVEEIRSFRAEMELMKSVGSHPHVVSL : 494

 * 560 * 580 * 600 * 620 *
Dros : VGYSTRFSNQMMLLIEYCSLGSLQNFLREEWKFRQEQNAIGLKKNLEQNVDNRRFNRLPRNSIHDRIEDINNSMLSTVEEESESDQTHSS : 625
Bomb : VGCCS--GRKPLIVAEYCSRGDLLSYLRSSWDIIVSKHTAKYYNNNMDSMDTSKLKVHKE------------------------------ : 559
Pnap : VGCFR--GRKPFIIAEYCSRGDLLTFLRCSWDLMVTRRNANYNNN--EEQDYRDIKTK-------------------------------- : 551
Prap : VGCFR--GRKPFIIAEYCSRGDLLTFLRCSWDLMVTRRNANYNNN--EEQDYRDIKTK-------------------------------- : 551
Pbra : VGCFR--GRKPFIIAEYCSRGDLLTFLRCSWDLMVTRRNGNYNNN--EEQDYRDIKTK-------------------------------- : 548


Dros : RCETYTLTRITNAADNKGYGLEDIENIGGSYIPKTAEAPKDRPKRKLKPQPKKDSKQDFKSDNKKRIFENKEYFDCLDSSDTKPRIPLKY : 715
Bomb : ---------HTKLVVNKLYELQGPCET-----------------------------------------------------------ELTP : 581
Pnap : ---------DSQLVINRLYDLQGICDT-----------------------------------------------------------ELTV : 573
Prap : ---------DSQLVINRLYDLQGICDT-----------------------------------------------------------ELTV : 573
Pbra : ---------DSQLVINRLYDLQGICDT-----------------------------------------------------------ELTV : 570


Dros : ADLLDIAQQVAVGMEFLAQNKVVHRDLAARNVLISVDRSIKIADFGLSRDVYHENVYRKSGGSGKLPIKWLALESLTHQVYTSQSDVWSF : 805
Bomb : LDLLSFCRQIAMGMEFLASNRIVHRDLAARNVLVTEDKTLKIADFGLSRDIYEENQYKQKG-NGKMPVKWMALESLTRRVYTTQSDVWSF : 670
Pnap : LDLLSFCRQIAMGMEFLASNRVVHRDLAARNILVTADRTLKIADFGLSRDVYQENQYKQKG-NGKMPVKWMALESLTHRIYTTLSDVWSF : 662
Prap : LDLLSFCRQIAMGMEFLASNRVVHRDLAARNILVTADRTLKIADFGLSRDVYQENQYKQKG-NGKMPVKWMALESLTHRIYTTLSDVWSF : 662
Pbra : LDLLSFCRQIAMGMEFLASNRVVHRDLAARNILVTADRTLKIADFGLSRDVYQENQYKQKG-NGKMPVKWMALESLTHRIYTTLSDVWSF : 659


Dros : GVLLYEITTLGGMPYPSVSPSDLLQLLRQGHRMKRPEGCTQEMFSLMESCWSSVPSHRPTFSALKHRLGGMILATNDVPERLKQLQAATE : 895
Bomb : GVVIWEIVTVGGSPYPEVPAARLVRSLRSGYRMPKPVNCSKPLYDIMRACWNASPRDRPTFPELHQKLDDLLHSACANEYITLEVDVDEA : 760
Pnap : GVVMWEIVTVGGAPYPSVGAARLPRLLRAGYRMPKPSNCSAQLYEVMLSCWNERPRSRPTFTELHRALDGLLC-ASAHHYLDLQLPPEPA : 751
Prap : GVVMWEIVTVGGAPYPSVGAARLPRLLRAGYRMPRPSNCSAQLYELMLSCWNERPRSRPTFTELHRALDGLLC-ASAHHYLDLQLPPEPA : 751
Pbra : GVVMWEIVTVGGAPYPSVGAARLPRLLRAGYRMPRPSNCSGQLYELMLSCWNERPRSRPTFTELHRALDGLLC-ASAHHYLDLQLPPEPA : 748


Dros : SKLKSCDGLNSKVEQ--VPCEEELYLEPLN----------------- : 923
Bomb : PSTPKPQRYIKMLIRGKLPWSRESYERPVNPTSNLYSSPPVIQTKTA : 807
Pnap : YPRPTTQRYVRMIMRGKWPWTN-RYHRSLTTKY-----APAANN--- : 789
Prap : YPRPTTQRYVRMIMRGKWPWTN-RYQRSLTTKY-----ASAVNN--- : 789
Pbra : YPRPTTQRYVRMIMRGKWPWTN-RYQRSLTTKY-----APAVNN--- : 786

**S3.2: Pair wise degrees** of Torso sequence identity (upper number), similarity (middle number) and gaps (lower number) based on a multiple sequence alignment in S3.1

Dros Bomb Pnap Prap Pbra

Dros 923 20% 20% 21% 21%

0 37% 37% 38% 38%

0 17% 17% 17% 17%

Bomb 198 807 44% 44% 44%

354 0 61% 61% 61%

164 0 5% 5% 5%

Pnap 195 368 789 93% 89%

355 503 0 96% 92%

160 44 0 0% 0%

Prap 203 364 735 789 89%

358 502 759 0 93%

160 44 0 0 0%

Pbra 204 364 706 704 786

360 501 731 735 0

163 47 3 3 0

Dros = *Drosphila melanogaster*

Bomb = *Bombyx mori*

Pnap = *Pieris napi*

Prap = *Pieris rapae*

Pbra = *Pieris brassicae*

**S3.3: Complete Torso amino acid sequence with the catalytic domain of the protein tyrosine kinases**

MHLKINEWLLIIKTFAFLPGLFADTSFTSLPLTEEQLKGLANDVCHDIFYEPKAAACTNKFFGTRYETKTEPPSVQVKCRAEQKLTFAVPQSKDWQLVVLIPEEVDMMEYFIFNAMEPAREIGSIYTPPGAFTLWSALLNDTGSPSIWKKSPILEAWNDTVADVEERNYVVNYTFLFDKFDNATADLDVKLTWNATDPSDSCFEVYNRCNSKTLGNVLHRSIGPDKNKSIIATVPLDDKCTILVKGKYGTTKFQYQTPSCYDLPGCKYLPEKPENVKLTAKENGDSWLVSVKWRQPKHPPSYYNVTLRADKVFTIKASNLSSEAKFTNVTGRGYYNVTVDAINDIGHAVTFQRSIFPLVEESASVSLLIGAWAEVIVISTIIATFFVWWKRQRDIKKRNMYFPGVREKVLKDGELEICCVESGSEEQWEVKPERLLLHEVIGEGAFGVVRRATLSPDDKIVAVKMLKDFPSVEEIRSFRAEMELMKSVGSHPHVVSLVGCFRGRKPFIIAEYCSRGDLLTFLRCSWDLMVTRRNANYNNNEEQDYRDIKTKDSQLVINRLYDLQGICDTELTVLDLLSFCRQIAMGMEFLASNRVVHRDLAARNILVTADRTLKIADFGLSRDVYQENQYKQKGNGKMPVKWMALESLTHRIYTTLSDVWSFGVVMWEIVTVGGAPYPSVGAARLPRLLRAGYRMPKPSNCSAQLYEVMLSCWNERPRSRPTFTELHRALDGLLCASAHHYLDLQLPPEPAYPRPTTQRYVRMIMRGKWPWTNRYHRSLTTKYAPAANN

Yellow: Catalytic domain of protein tyrosine kinases

**S4.1 Multiple sequence alignment of the Ultraspiracle protein sequence** between Pieris napi (Pnap), Pieris rapae (Prap), Danaus plexippus (Danaus), Bombyx mori (Bomb), Manduca sexta (Mand) Drosophila melanogaster (Drosi), and the retinoid x receptor in Homo sapiens (Hsap).

Pnap : --MSSVAKKDKPTMSVTALMQWARPGAPGPP-----ATPTP-APMLQSPCTPT---NVDFSIDMQWLNLEPGFMSPMSPPEMKPDTAMLD : 79
Prap : --MSSVAKKDKPTMSVTALMQWARPGPPGPP-----ATPTP-APILQSPGTPT---NVDFSIDMQWLNLEPGFMSPMSPPEMKPDTAMLD : 79
Danaus : --MSSVAKKDKPTMSVTALINWARPAPPGPQQQLAQAVPVSSTALLQSLGTSSNIPNVDCSIDMQWLNIESGFMSPMSPPEMKPDTAMLD : 88
Bomb : --MSSVAKKDKPTMSVTALINRAWPMTPSPQQQQQMVPSTQHSNFLQPMATPS--TTPNVELDIQWLNIESGFMSPMSPPEMKPDTAMLD : 86
Mand : --MSSVAKKDKPTMSVTALINRAWPLTPAPHQQQ-SMPSSQPSNFLQPLATPS--TTPSVELDIQWLNIEPGFMSPMSPPEMKPDTAMLD : 85
Drosi : --MDNCDQD--ASFRLSHIKEEVKPDISQLN--------DSNNSSFSPKAESP----VPFMQAMSMVHVLPGSNSASSNNNSAGDAQMAQ : 74
Hsap : MAAPSLHPSLGPGIGSPGQLHSPISTLSSPIN----GMGPPFSVISSPMGPHS--MSVPTTPTLGFSTGSPQLSSPMNPVSSSEDIKPPL : 84


Pnap : GG-MRDDATSPPALRSYPPNHPLSGSKHLCSICGDRASGKHYGVYSCEGCKGFFKRTVRKDLTYACREERNCIIDKRQRNRCQFCRYQKC : 168
Prap : GG-MRDDATSPPALRNYPPNHPLSGSKHLCSICGDRASGKHYGVYSCEGCKGFFKRTVRKDLTYACREERNCIIDKRQRNRCQFCRYQKC : 168
Danaus : G--MREDATSPSAMRNYPPNHPLSGSKHLCSICGDRASGKHYGVYSCEGCKGFFKRTVRKDLTYACREERNCIIDKRQRNRCQYCRYQKC : 176
Bomb : G--FRDDSTPPPPFKNYPPNHPLSGSKHLCSICGDRASGKHYGVYSCEGCKGFFKRTVRKDLTYACREDKNCIIDKRQRNRCQYCRYQKC : 174
Mand : G--LRDDSTPPPAFKNYPPNHPLSGSKHLCSICGDRASGKHYGVYSCEGCKGFFKRTVRKDLTYACREDRNCIIDKRQRNRCQYCRYQKC : 173
Drosi : APNSAGGSAAAAVQQQYPPNHPLSGSKHLCSICGDRASGKHYGVYSCEGCKGFFKRTVRKDLTYACRENRNCIIDKRQRNRCQYCRYQKC : 164
Hsap : G------LNGVLKVPAHPSGNMASFTKHICAICGDRSSGKHYGVYSCEGCKGFFKRTVRKDLTYTCRDNKDCLIDKRQRNRCQYCRYQKC : 168


Pnap : LACGMKREAVQEERQRAAR-----------------------------------------GAEDAHPSSSVQELSIERLLEMESLVADPS : 217
Prap : LACGMKREAVQEERQRAAR-----------------------------------------GAEDAHPSSSVQELSIERLLEMESLVADPS : 217
Danaus : LACGMKREAVQEERQRAAR-----------------------------------------GAEDVHPSSSVQELSIERLLEMESLVADPN : 225
Bomb : LACGMKREAVQEERQRAAR-----------------------------------------GTEDAHPSSSVQELSIERLLELEALVADSA : 223
Mand : LACGMKREAVQEERQRAAR-----------------------------------------GTEDAHPSSSVQELSIERLLEIESLVADPP : 222
Drosi : LTCGMKREAVQEERQRGARNAAGRLSASGGGSSGPGSVGGSSSQGGGGGGGVSGGMGSGNGSDDFMTNSVSRDFSIERIIEAEQRAETQC : 254
Hsap : LAMGMKREAVQEERQRGKDR---------------------------------------NENEVESTSSANEDMPVERILEAELAVEPKT : 219


Pnap : EE--FQFLRVGPDTNVPPRYRAPVSSLCQIGNKQIAALVVWARDIPHFSQLEMEDQVLLIKSSWNELLLFAIAWRSIEYLEDERE----- : 300
Prap : EE--FQFLRVGPETNVPPRYRAPVSSLCQIGNKQIAALVVWARDIPHFSQLEMEDQVLLIKSSWNELLLFAIAWRSIEYLEDERE----- : 300
Danaus : EE--FQFLRVGPDSNVPPRYRAPVSSLCQIGNKQIAALVVWARDIPHFSQLELEDQVILIKASWNELMLFAIAWRSMEYLEDERE----- : 308
Bomb : EE--LQILRVGPESGVPAKYRAPVSSLCQIGNKQIAALIVWARDIPHFGQLEIDDQILLIKGSWNELLLFAIAWRSMEFLNDERE----- : 306
Mand : EE--FQFLRVGPESGVPAKYRAPVSSLCQIGNKQIAALVVWARDIPHFGQLELEDQILLIKNSWNELLLFAIAWRSMEYLTDERE----- : 305
Drosi : GDRALTFLRVGPYSTVQPDYKGAVSALCQVVNKQLFQMVEYARMMPHFAQVPLDDQVILLKAAWIELLIANVAWCSIVSLDDGGAGGGGG : 344
Hsap : ET--YVEANMGLNPSSP---NDPVTNICQAADKQLFTLVEWAKRIPHFSELPLDDQVILLRAGWNELLIASFSHRSIAVKD--------- : 295


Pnap : ------NMDGTRTASPPQLMCLMPGMTLHRNSALQAGVGQIFDRVLSELSLKMRALRMDQAEYVALKAIVLLNPDVKGLKNRQEVDLLRE : 384
Prap : ------NMDGTRTASPPQLMCLMPGMTLHRNSALQAGVGQIFDRVLSELSLKMRALRMDQAEYVALKAIVLLNPDVKGLKNRQEVDVLRE : 384
Danaus : ------NLDGTRTAPPPQLMCLMPGMTLHRNSALQAGVGQIFDRVLSELSLKMRALRMDQAEYVALKAIVLLNPDIKGLKNRQDVDVLRE : 392
Bomb : ------NVD-SRNTAPPQLICLMPGMTLHRNSALQAGVGQIFDRVLSELSLKMRSLRMDQAEYVALKAIILLNPDVKGLKNKQEVDVLRE : 389
Mand : ------NVD-SRSTAPPQLMCLMPGMTLHRNSALQAGVGQIFDRVLSELSLKMRTLRMDQAEYVALKAIILLNPDVKGLKNKPEVVVLRE : 388
Drosi : GLGHDGSFERRSPGLQPQQLFLNQSFSYHRNSAIKAGVSAIFDRILSELSVKMKRLNLDRRELSCLKAIILYNPDIRGIKSRAEIEMCRE : 434
Hsap : ------------------GILLATGLHVHRNSAHSAGVGAIFDRVLTELVSKMRDMQMDKTELGCLRAIVLFNPDSKGLSNPAEVEALRE : 367


Pnap : KIFSCLDEYCRRSHSTEEGRFASLILRLPALRSISLKSFEHLFFFHLVAEGSISSFIREALRMHAPPIDANSMM : 458
Prap : KMFSCLDEYCRRSHSTEEGRFASLLLRLPALRSISLKSFEHLFFFHLVAEGSISSFIREALRMHAPPIDANSMM : 458
Danaus : KMFSCLDEYCRRAHSSEEGRFASLLLRLPALRSISLKSFEHLFFFHLIAEGTIGTYIRDALRSHAPTIDTNSIM : 466
Bomb : KMFLCLDEYCRRSRGGEEGRFAALLLRLPALRSISLKSFEHLYLFHLVAEGSVSSYIRDALCNHAPPIDTNIM- : 462
Mand : KMFSCLDEYVRRSRCAEEGRFAALLLRLPALRSISLKCFEHLYFFHLVADTSIASYIHDALRNHAPSIDTSIL- : 461
Drosi : KVYACLDEHCRLEHPGDDGRFAQLLLRLPALRSISLKCQDHLFLFRITSDRPLEELFLEQLEAPPPPGLAMKLE : 508
Hsap : KVYASLEAYCKHKYPEQPGRFAKLLLRLPALRSIGLKCLEHLFFFKLIGDTPIDTFLMEMLEAPHQMT------ : 435

Pnap: *Pieris napi*

Prap: *Pieris rapae*

Danaus: *Danaus plexippus*

Bomb: *Bombyx mori*

Mand: *Manduca sexta*

Drosi: *Drosophila melanogaster*

Hsap: *Homo sapiens* retinoid X receptor alpha

**S4.2 Pair wise degrees** of USP sequence identity (upper number), similarity (middle number) and gaps (lower number) based on a multiple sequence alignment in S4.1

Pnap Prap Danaus Bomb Mand Drosi Hsap

Pnap 458 98% 87% 79% 80% 42% 42%

0 99% 93% 88% 88% 58% 58%

0 0% 2% 2% 1% 12% 7%

Prap 450 458 88% 80% 81% 43% 42%

456 0 93% 88% 88% 58% 58%

0 0 2% 2% 1% 12% 7%

Danaus 407 412 466 79% 81% 42% 42%

436 438 0 88% 88% 57% 58%

10 10 0 0% 1% 13% 8%

Bomb 370 375 372 462 90% 41% 41%

413 413 413 0 95% 56% 56%

10 10 4 0 0% 13% 7%

Mand 373 378 378 416 461 42% 41%

412 412 413 441 0 56% 56%

9 9 5 1 0 13% 7%

Drosi 221 222 222 217 219 508 38%

300 300 301 293 292 0 52%

62 62 70 70 69 0 17%

Hsap 196 199 198 193 195 198 435

273 272 273 262 264 270 0

37 37 39 35 34 93 0

**S4.3:** **Complete amino acid sequence of Ultraspiracle** with DNA-binding domain in yellow and the ligand binding domain in green

MDSRDPGLNLEPGFMSPMSPPEMKPDTAMLDGGMRDDATSPPALRSYPPNHPLSGSKHLCSICGDRASGKHYGVYSCEGCKGFFKRTVRKDLTYACREERNCIIDKRQRNRCQFCRYQKCLACGMKREAVQEERQRAARGAEDAHPSSSVQELSIERLLEMESLVADPSEEFQFLRVGPDTNVPPRYRAPVSSLCQIGNKQIAALVVWARDIPHFSQLEMEDQVLLIKSSWNELLLFAIAWRSIEYLEDERENMDGTRTASPPQLMCLMPGMTLHRNSALQAGVGQIFDRVLSELSLKMRALRMDQAEYVALKAIVLLNPDVKGLKNRQEVDLLREKIFSCLDEYCRRSHSTEEGRFASLILRLPALRSISLKSFEHLFFFHLVAEGSISSFIREALRMHAPPIDANSMM

Yellow: DNA-binding domain of retinoid X receptor (RXR) is composed of two C4-type zinc fingers

Green: The ligand binding domain of the retinoid X receptor and Ultraspiracle, members of nuclear receptor superfamily

**S5.1 Multiple sequence alignment of the ecdysteroid receptor protein sequence** between Pieris napi (Pnapi), Pieris rapae (Prapa), Danaus plexippus (Danaus), Bombyx mori (Bomb), Manduca sexta (Mand).

Manduca : MRRRWSNNGCFPLRMFEESSSEVTSSSAFGMPAAMVMSPESLASPEYGGLELWSYDETMTNYPAQSLLGACNAPQQQQQQQQQQPSAQPL : 90
Bombyx : MRRRWSDNGGFPLRMLEESSTEVTSSSALGLPPAMVMSPESLASPEYGALELWSYDDGITYNTAQSLLGACNMQQQQLQPQQPHPAPPTL : 90
Danaus : MRRRWSNNGGFPLRMLEESSSEVTSSSALGLPPAMVMSPESLASPEYGGLELWGYDDGITYNTAQSLLGNTCT------LQQQQPPTQPL : 84
Pnapi : MRRRWSNNGGFPLRMLEESSSEVTSSSALGLSAAMVMSPESLASPEYG-LELWGYDDGISYNTTQSLLGTHCT------MQQQQPQTQPL : 83
Prapa : MRRRWSNNGGFPLRMLEESSSEVTSSSALGLSAAMVMSPESLASPEYG-LELWGYDDGISYNATQSLLGTHCT------MQQQQPQTQPL : 83


Manduca : PSMPLPMPPTTPKSENESMSSGREELSPASSINGCSTDGEPRRQKKGPAPRQQEELCLVCGDRASGYHYNALTCEGCKGFFRRSVTKNAV : 180
Bombyx : PTMPLPMPPTTPKSENESMSSGREELSPASSINGCSADADARRQKKGPAPRQQEELCLVCGDRASGYHYNALTCEGCKGFFRRSVTKNAV : 180
Danaus : PSMPLPMPPTTPKSENESISSGREELSPASSVNGCSTDGEARRQKKGPVPRQQEELCLVCGDRASGYHYNALTCEGCKGFFRRSVTKNAV : 174
Pnapi : PSMPLPMPPTTPKSENESISSGREELSPASSINGCSTDGDARRQKKGPAPRQQEELCLVCGDRASGYHYNALTCEGCKGFFRRSVTKNAV : 173
Prapa : PSMPLPMPPTTPKSENESISSGREELSPASSINGCSTDGDARRQKKGPAPRQQEELCLVCGDRASGYHYNALTCEGCKGFFRRSVTKNAV : 173


Manduca : YICKFGHACEMDMYMRRKCQECRLKKCLAVGMRPECVVPESTCKNKRREKEAQREKDKLPVSTTTVDDHMPAIMQCDPPPPEAARILECL : 270
Bombyx : YICKFGHACEMDMYMRRKCQECRLKKCLAVGMRPECVIQEP-SKNKDRQRQKKDKGILLPVSTTTVEDHMPPIMQCDPPPPEAARI---- : 265
Danaus : YICKFGHACEMDMYMRRKCQECRLKKCLAVGMRPECVVPENQCAIKRKEKKAQREKDKLPVSTTTVDDHMPPIMQCDPPPPEAARILECL : 264
Pnapi : YICKFGHACEMDMYMRRKCQECRLKKCLAVGMRPECVVPEPQCALKRKEKKAQREKDKLPVSTTTVDDHMPPIMQCDPPPPDAARILECL : 263
Prapa : YICKFGHACEMDMYMRRKCQECRLKKCLAVGMRPECVVPEPQCALKRKEKKAQREKDKLPVSTTTVDDHMPPIMQCDPPPPDAARILECL : 263


Manduca : QHEVVPRFLTEKLMEQNRLKNVTPLSANQKSLIARLVWYQEGYEQPSEEDLKRVTQTWQLEEEEEEETDMPFRQITEMTILTVQLIVEFA : 360
Bombyx : -HEVVPRYLSEKLMEQNRQKNIPPLSANQKSLIARLVWYQEGYEQPSDEDLKRVTQ----SDEEDEESDLPFRQITEMTILTVQLIVEFA : 350
Danaus : QHEVVPRFLSEKLLEQNRQKNIPALTSNQQFLIARLVWYQDGYEQPSEEDLKRVTQTWQQTDQDDEDSDMPFRQITEMTILTVQLIVEFA : 354
Pnapi : QHEVVPRFLSEKLLEQNRLKNIPPLSPNQQFLIARLVWYQDGYEQPSEEDLKRVTQTWQHGEEDDGDTDLAFRQITEMTILTVQLIVEFA : 353
Prapa : QHEVVPRFLSEKLLEQNRLKNIPPLSPNQQFLIARLVWYQDGYEQPSEEDLKRVTQTWQHGEEDDGDTDLAFRQITEMTILTVQLIVEFA : 353


Manduca : KGLPGFSKISQSDQITLLKASSSEVMMLRVARRYDAATDSVLFANNQAYTRDNYRKAGMSYVIEDLLHFCRCMYSMSMDNVHYALLTAIV : 450
Bombyx : KGLPGFSKISQSDQITLLKASSSEVMMLRVARRYDAASDSVLFANNKAYTRDNYRKAGMAYVIEDLLHFCRCMFAMGMDNVHFALLTAIV : 440
Danaus : KGLPGFAKISQPDQITLLKACSSEVMMLRVARRYDAATDSVLFANNQAYTRDNYRKAGMAYVIEDLLHFCRCMYAMSMDNVHYALLTAIV : 444
Pnapi : KGLPGFGKISQPDQITLLKACSSEVMMLRVARRYDASTDSILFANNEAYTRDNYRKAGMSYVIEDLLHFCRCMYALSLDNVHYALLTAVI : 443
Prapa : KGLPGFGKISQPDQITLLKACSSEVMMLRVARRYDASTDSILFANNEAYTRDNYRKAGMSYVIEDLLHFCRCMYALSLDNVHYALLTAVI : 443


Manduca : IFSDRPGLEQPLLVEEIQRYYLKTLRVYILNQHSASPRCAVLFGKILGVLTELRTLGTQNSNMCISLKLKNRKLPPFLEEIWDVAEVSTT : 540
Bombyx : IFSDRPGLEQPSLVEEIQRYYLNTLRIYIINQNSASSRCAVIYGRILSVLTELRTLGTQNSNMCISLKLKNRKLPPFLEEIWDVAEVPTT : 530
Danaus : IFSDRPGLEQPQLVEEIQRYYLNTLRVYIMNQLSASSRCPVVYGKILSILSELRTLGMQNSNMCISLKLKNRKLPPFLEEIWDVADVSTA : 534
Pnapi : IFSDRPGLEQPNLVEEIQRYYLTTLRMYIVNQLSASSRCSVLFGKILSILSEVRTLGMQNSNMCISLKLKNRKLPPFLEEIWDVADV-TS : 532
Prapa : IFSDRPGLEQPNLVEEIQRYYLTTLRMYILNQLSASPRCSVLFGKILSILSEVRTLGMQNSNMCISLKLKNRKLPPFLEEIWDVADV-TS : 532


Manduca : QPTPGVAAQVTPIVVDNPAAL : 561
Bombyx : HPTVLP--------PTNPVVL : 543
Danaus : QPPP---------IVDNPVDL : 546
Pnapi : QPPA---------LLDNATEL : 544
Prapa : QPPA---------LLDHATEL : 544

Manduca: *Manduca sexta*

Bombyx: *Bombyx mori*

Danaus: *Danaus plexippus*

Pnapi: *Pieris napi*

Prapa: *Pieris rapae*

**S5.2 Pair wise degrees** of ecdysteroid receptor sequence identity (upper number), similarity (middle number) and gaps (lower number) based on a multiple sequence alignment in S5.1

Manduca Bombyx Danaus Pnapi Prapa

Manduca 561 81% 84% 82% 82%

0 88% 90% 89% 89%

0 3% 2% 3% 3%

Bombyx 460 543 81% 79% 78%

499 0 88% 87% 87%

18 0 3% 3% 3%

Danaus 474 453 546 90% 89%

507 493 0 95% 95%

15 19 0 0% 0%

Pnapi 464 439 494 544 99%

502 487 523 0 99%

17 21 2 0 0%

Prapa 465 436 491 540 544

503 485 521 542 0

17 21 2 0 0

**S5.3: Complete amino acid sequence of the ecdysteroid receptor** with the ligand binding domain in yellow and the DNA-binding domain in green.

MRRRWSNNGGFPLRMLEESSSEVTSSSALGLSAAMVMSPESLASPEYGLELWGYDDGISYNTTQSLLGTHCTMQQQQPQTQPLPSMPLPMPPTTPKSENESISSGREELSPASSINGCSTDGDARRQKKGPAPRQQEELCLVCGDRASGYHYNALTCEGCKGFFRRSVTKNAVYICKFGHACEMDMYMRRKCQECRLKKCLAVGMRPECVVPEPQCALKRKEKKAQREKDKLPVSTTTVDDHMPPIMQCDPPPPDAARILECLQHEVVPRFLSEKLLEQNRLKNIPPLSPNQQFLIARLVWYQDGYEQPSEEDLKRVTQTWQHGEEDDGDTDLAFRQITEMTILTVQLIVEFAKGLPGFGKISQPDQITLLKACSSEVMMLRVARRYDASTDSILFANNEAYTRDNYRKAGMSYVIEDLLHFCRCMYALSLDNVHYALLTAVIIFSDRPGLEQPNLVEEIQRYYLTTLRMYIVNQLSASSRCSVLFGKILSILSEVRTLGMQNSNMCISLKLKNRKLPPFLEEIWDVADVTSQPPALLDNATEL

Yellow: The Ligand Binding Domain (LBD) of the Ecdysone Receptor, a member of the nuclear receptors super family.

Green: DNA- Binding Domain of the Ecdysone Receptor (EcR) family is composed of two C4-type Zinc Fingers.

**S6.1 Multiple sequence alignment of the FoxO protein sequence** between Pieris napi (Pnapi), Pieris rapae (Prapa), Danaus plexippus (Danaus), Bombyx mori (Bomb), Manduca sexta (Mand), Drosophila melanogaster (Dros), Rhodnius prolixus (Rhod), Cimex lectularius (Cimex), and Homo sapiens (Hsap).

Pnap : ------------------------------MSLGGGS-YHSPWSSQTALSELEGAMGELEPLGELAEVGFEPQTRARSNTWPLPRPENYV : 59
Prap : ------------------------------MSLGGGS-YHSPWSSQTALSELEGAMGELEPLGELAEVGFEPQTRARSNTWPLPRPENYV : 59
Bmor : ------------------------------MSIQGGGGYQSPWSSQTGLSELEGTMAELEPLGELAEVGFEPQTRARSNTWPLPRPDNYV : 60
Dplex : ------------------------------MSLQRGI-YQSPWSSQTALSELEG-MGELEPLGELGEVGFEPQTRARSNTWPLPRPENYI : 58
Mand : ------------------------------MSIQGGGGYQSPWSSQTGLSELEGTMGELEPLGELTEVGFEPQTRARSNTWPLPRPDNYV : 60
Dros : --------------------------------MMDGYAQEWPRLTHTDNGLAMDQLGGDLPL----DVGFEPQTRARSNTWPCPRPENFV : 54
Rhod : ---------------------------------------MEPPSAMTGS-----------------EMGLEPQIRARSNTWPLPRPDNFV : 34
Cimex : ---------------------------------------MDP--SVTEV-----------------EMGMEPQTRARSNTWPLPRPENFS : 32
Hsap : MAEAPQVVEIDPDFEPLPRPRSCTWPLPRPEFSQSNSATSSPAPSGSAAANPDAAAGLPSASAAAVSADFMSNLSLLEESEDFPQAPGSV : 90


Pnap : DPAEDGGSKKNSNQNLTGA-----------------------------PPLP-----IATKKNSSRRNAWGNLSYADLITQAITSSPDKR : 115
Prap : DPVEDGGSKKNSNQNLTGA-----------------------------PPLP-----IATKKNSSRRNAWGNLSYADLITQAITSSQDKR : 115
Bmor : EQVDEAGSKKNSNQNLSGA-----------------------------PPIP-------AKKNSSRRNAWGNLSYADLITQAITSAQDNR : 114
Dplex : EP-DDGGSKKNSNQNLTGA-----------------------------PPLPSTS--GTTKKNSSRRNAWGNLSYADLITQAITSSQDNR : 116
Mand : DPVDDTGSKKNSNQNLAGA-----------------------------PPLPS----VGGKKNSSRRNAWGNLSYADLITQAITSAQDNR : 117
Dros : EPTDELDSTKASNQQLAPG-----------------------------DSQQAIQNANAAKKNSSRRNAWGNLSYADLITHAIGSATDKR : 115
Rhod : ENDDNVVEMSEQKCSASGAGVIVGA---------------GLTGAAAATGITGAPPSTHLKKNSSRRNAWGNLSYADLITQAIGSAPDKR : 109
Cimex : EPDSSSVEMAEQKCGVVAA----------------------------------LPGASHLKKNSSRRNAWGNLSYADLITQAIGSAPDKR : 88
Hsap : AAAVAAAAAAAATGGLCGDFQGPEAGCLHPAPPQPPPPGPLSQHPPVPPAAAGPLAGQPRKSSSSRRNAWGNLSYADLITKAIESSAEKR : 180


Pnap : LTLSQIYEWMVQNVPYFKDKGDSNSSAGWKNSIRHNLSLHNRFMRVQNEGTGKSSWWMINP-DAKPGKSVRRRALSMET-SKAEKRRGRV : 203
Prap : LTLSQIYEWMVQNVPYFKDKGDSNSSAGWKNSIRHNLSLHNRFMRVQNEGTGKSSWWMINP-DAKPGKSVRRRALSMET-SKAEKRRGRV : 203
Bmor : LTLSQIYEWMVQNVPYFKDKGDNNSSAGWKNSIRHNLSLHNRFMRVQNEGTGKSSWWMINP-DAKPGKSVRRRAASMET-SKFEKRRGRV : 202
Dplex : LTLSQIYEWMVQNVPYFKDKGDSNSSAGWKNSIRHNLSLHNRFMRVQNEGTGKSSWWMINP-DAKPGKSVRRRALSMET-SKSEKRRGRV : 204
Mand : LTLSQIYEWMVQNVPYFKDKGDSNSSAGWKNSIRHNLSLHNRFMRVQNEGTGKSSWWMINP-DAKPGKSVRRRAASMET-SKFEKRRGRL : 205
Dros : LTLSQIYEWMVQNVPYFKDKGDSNSSAGWKNSIRHNLSLHNRFMRVQNEGTGKSSWWMLNP-EAKPGKSVRRRAASMET-SRYEKRRGRA : 203
Rhod : LTLSQIYEWMVQNVAYFKDKGDSNSSAGWKNSIRHNLSLHNRFMRVQNEGTGKSSWWMINP-DAKPGKSARRRAASMET-SKFEKRRGRI : 197
Cimex : LTLSQIYEWMVHNVAYFKDKGDSNSSAGWKNSIRHNLSLHNRFMRVQNEGTGKSSWWMINP-EAKPGKSARRRATSMET-SKFEKRRGRV : 176
Hsap : LTLSQIYEWMVKSVPYFKDKGDSNSSAGWKNSIRHNLSLHSKFIRVQNEGTGKSSWWMLNPEGGKSGKSPRRRAASMDNNSKFAKSRSRA : 270


Pnap : KKKPESLRNG---MTADTTPSPSSSVSESHDIFPDSPVPSST-SFQLSPDFRQRASSNASSCG-RLSPIPSLLHTEPDW--QTDYGSTEF : 286
Prap : KKKPESLRNG---MTADTTPSPSSSISESHDIFPDSPVPSST-SFQLSPDFRQRASSNASSCG-RLSPIPSLLHTEPDW--QTDYGSTEF : 286
Bmor : KKKTEALRNG---ATADATPSPSSSVSESIDIFTDSPMHSS--SFQLSPDFRQRAPSNASSCG-RLSPIPSMIPSEPDW--KQEYAN--- : 281
Dplex : KKKPEALRNG---VAADATPSPSSSISESVDLFPDSPIHSG--SFQLSPDFRQRAPSNASSCG-RLSPIPSLIPSEPDW--ATDYTP--- : 283
Mand : KKKTEALRHG---ATADATPSPSSSISESIDTFPDSPMHSS--SFQLSPDFRQRASSNASSCG-RLSPIPSLIPSEPDW--ASEYTPS-- : 285
Dros : KKRVEALRQAGVVGLNDATPSPSSSVSEGLDHFPESPLHSGG-GFQLSPDFRQRASSNASSCG-RLSPIR-AQDLEPDWGFPVDYQNTTM : 290
Rhod : KKKVDAIRSG-----LEATPSPSSSVSESLDLFPESPLH----GFQLSPDFRPRTSSNASSCGGRLSPIP---SVETDWG---------- : 265
Cimex : KKKVEALRSG-----LDATPSPSSSVSESLDLFPESPLH----GFQLSPDFRPRASSNASSCG-RLSPIP---AADTDWG---------- : 243
Hsap : AKKKASLQSG--------QEGAGDSPGSQFSKWPASPGSHSNDDFDNWSTFRPRTSSNASTISGRLSPIMTEQDDLGEGDVHSMVYPP-- : 350


Pnap : GSADFGSADFTSNTDY--QDFTQDE-LAGTLADSMKLHG-DPFLN--TYVPTTSSSSSGGSYRF-----AY-TCPRHP----HGGCACAS : 360
Prap : GSADFGSADFTSNTDY--QDFTQDE-LAGTLADSMKLHG-DPFLN--TYVPTTSSSSSGGSYRF-----AY-TCPRHP----HGGCACAS : 360
Bmor : --------DYATNTDFSQADYTQEEQLAGSLADSMKLQGADPFLN--PYVPTTSSSTSGGSFRYG----SYGTCPRHP----HGGCTCAS : 353
Dplex : ------AGDFTTTSDFTQADYAQDE-LAGTLADSMKLHGADPYLN--TYVPTTSSS---GNYRF-----PY-TCLRHP----HGGCTCTS : 351
Mand : -------GDYATNSDFTQTDFAQEEQLAGSLAVSMKLHGADPFLN--TYVPTTSSSTSGGSFRYG----SYGACPRHP----HGGCACAS : 358
Dros : TQAHAQALEELTGTMADELTLCNQQQQGFSAASGLPSQPPPPPYQPPQHQQAQQQQQQQSPYALNGPASGYNTLQPQSQCLLHRSLNCSC : 380
Rhod : --------------------YYGPEQLAGSLEQTMRLGQQQQQQQ--QPAQQQQSAQQQQSQQQQ---------SQQA-----RTRPTGG : 319
Cimex : --------------------YYSPEQLAGNLEQTMRLGQQQQQQ----------------------------------------AAGNGA : 273
Hsap : -------SAAKMASTLPSLSEISNPENMENLLDNLNLLSSPTSLTVSTQSSPGTMMQQTPCYSFAPPNTSLNSPSPNYQKYTYGQSSMSP : 433


Pnap : LF-----PAHPAHPHQHALDHFVRPPPPADPADIMQTEN-STQMVTTSDAALMNGGMMVQTG-AMGPTTVMGQIMGALN--TGLAED--- : 438
Prap : LF-----PAHPAHPHQHALDHFVRPPPPADPADIMQTEN-STQMVTTSDAALMNGGMMVQTG-AMGPTTVMGQIMGALN--TGLAED--- : 438
Bmor : MYSHPAHPAHPTHPHQHALDHFVRPPPSADQADIMQTGNRQTQMVTTSDPALMNGGMMVQAG-GMGPTTVMGQIMGALN--SGLVED--- : 437
Dplex : LY-----PAHPAHTH----DHFVRPPPPADSADIMQTEN-SQPQMVTTDAALMNGGIMVQPG-AMGPTTVMGQIMGALN--TGLAED--- : 425
Mand : MYPHPAHPAHPTHPHQHALDHFVRPPAPADPADIMQTENGQTQMVTTSDPALMNGGMMLQTG-ALGPTTVMGQIMGALN--SGLGED--- : 442
Dros : MHN----ARDGLSPNSVTTTMSPAYPNSEPSSDSLNTYSNVVLDGPADTAALMVQQQQQQQQQQQLSASLEGQCLEVLNNEAQPIDE--- : 463
Rhod : -------PGTPQDLPPPGGFAYPPPPPYRPAPPYMAPCPVHRLPQPCHTCAVAPKQKLFGGE-GESAATMMGQLMGALNPVSMLDEVN-- : 399
Cimex : -------TPTPQDIPSSG-FAYPPPPPYRPAPPYMAPCPVHRQHLPCPSCAVAPKQRMGYGE-GESATTMMGQLMGALNPT-MLDDLN-- : 351
Hsap : LPQMPIQTLQDNKSSYGGMSQYNCAPGLLKELLTSDSPPHNDIMTPVDPGVAQPNSRVLGQNVMMGPNSVMSTYGSQASHNKMMNPSSHT : 523


Pnap : -----------------INIESLEHS--FDCNVDEVIKHELSMDGSLDFN---------------------------------------- : 469
Prap : -----------------INIESLEHS--FDCNVDEVIKHELSMDGSLDFN---------------------------------------- : 469
Bmor : -----------------LNFEALEHG--FDCNVDEVIKHELSMDGSLDFN---------------------------------------- : 468
Dplex : -----------------LNIETLEHS--FDCNVEEVINHELRMDGTLDFN---------------------------------------- : 456
Mand : -----------------LNFETLEHG--FDCNVDEVIKHELSMDGTLDFN---------------------------------------- : 473
Dros : -----------------FNLENFPVGN-LECNVEELLQQEMSYGGLLDINIPLATVNTNLVNSSSGPLSISNISNLSNISSNSGSSLSLN : 535
Rhod : -----------------INIETLPLHGGFDCNVDEVIKHELSLDGTLDFN---------------------------------------- : 432
Cimex : -----------------LNIETVPIHGGFDCNVDEVIKHELSLDGTLDFN---------------------------------------- : 384
Hsap : HPGHAQQTSAVNGRPLPHTVSTMPHTSGMNRLTQVKTPVQVPLPHPMQMSALGG---------------------YSSVSSCNGYGRMGL : 592

Pnap : -----FPQQHTAMAAEAESQFAAP--------------------------APPVPTTLSGGGQ-RTPYTVA--PSWVH- : 513
Prap : -----FPQQHTVMAAEAESQFVAP--------------------------APPVPTTLSGGGQ-RTPYTVA--PSWVH- : 513
Bmor : -----FPQQQTAMAAEAESQFVAP--------------------------APPVPTTLSGGGTPRTAYSVT--PSWVH- : 513
Dplex : -----FQQG---MAAEAESQFVAP--------------------------APPVPTTLSGGG---PPYSVA--PSWV-- : 494
Mand : -----FPQQHGAMAAEAESQFAAP--------------------------APPVPSTLSGGGAPRTPYSAT--PSWVH- : 518
Dros : QLQAQLQQQQQQQQAQQQQQAQQQQQQHQQHQQQLLLNNNNNSSSSLELATQTATTNLNARVQYSQPSVVTSPPSWVH- : 613
Rhod : -----FTGGGGGGGSEA---------------------------------ASVLSSAGQTQETAAQPFSGH---SWVH- : 469
Cimex : -----FSG------------------------------------------------QQAPPNPDQPPFSGH---SWVH- : 406
Hsap : LHQEKLPSDLDGMFIERLDCDMESIIRNDLMDG----------------DTLDFNFDNVLPNQSFPHSVKTTTHSWVSG : 655

Pnap: *Pieris napi*

Prap_ *Pieris rapae*

Bmor: *Bombyx mori*

Dplex: *Danaus plexippus*

Mand: *Manduca sexta*

Dros: *Drosophila melanogaster*

Rhod: *Rhodnius* *prolixus*

Cimex: *Cimex lectularius*

Hsap: *Homo sapiens* FoxOX1

**S6.2 Pair wise degrees** of FoxO sequence identity (upper number), similarity (middle number) and gaps (lower number) based on a multiple sequence alignment in S6.1

Pnap Prap Bmor Dplex Mand Dros Rhod Cimex Hsap

Pnap 513 99% 79% 81% 80% 39% 44% 45% 23%

0 99% 86% 86% 87% 48% 52% 51% 32%

0 0% 5% 5% 4% 18% 19% 25% 25%

Prap 508 513 80% 81% 80% 39% 44% 45% 23%

509 0 87% 86% 87% 48% 52% 51% 32%

0 0 5% 5% 4% 18% 19% 25% 25%

Bmor 421 422 513 78% 89% 39% 46% 46% 22%

457 459 0 84% 92% 48% 53% 52% 31%

28 28 0 6% 0% 19% 18% 24% 23%

Dplex 421 424 406 494 79% 39% 45% 47% 22%

449 451 437 0 85% 47% 53% 53% 31%

31 31 33 0 5% 21% 20% 24% 26%

Mand 426 427 462 415 518 38% 46% 46% 23%

462 462 480 443 0 49% 53% 52% 32%

25 25 5 30 0 18% 18% 25% 22%

Dros 244 243 248 242 243 613 36% 35% 22%

301 301 305 297 307 0 44% 42% 31%

114 114 122 133 117 0 28% 34% 21%

Rhod 243 241 254 243 254 227 469 71% 22%

286 285 288 286 290 280 0 77% 30%

106 106 100 107 99 178 0 13% 29%

Cimex 240 238 242 242 243 218 334 406 22%

273 272 275 273 276 261 362 0 28%

135 135 131 124 132 211 63 0 38%

Hsap 156 155 147 150 154 156 148 146 655

216 216 211 211 213 225 201 188 0

170 170 152 173 147 150 192 255 0

**S6.3: Complete amino acid sequence for the Forkhead transcription factor** with Forkhead domain in yellow and transactivation domain in green.

MSLGGGSYHSPWSSQTALSELEGAMGELEPLGELAEVGFEPQTRARSNTWPLPRPENYVDPAEDGGSKKNSNQNLTGAPPLPIATKKNSSRRNAWGNLSYADLITQAITSSPDKRLTLSQIYEWMVQNVPYFKDKGDSNSSAGWKNSIRHNLSLHNRFMRVQNEGTGKSSWWMINPDAKPGKSVRRRALSMETSKAEKRRGRVKKKPESLRNGMTADTTPSPSSSVSESHDIFPDSPVPSSTSFQLSPDFRQRASSNASSCGRLSPIPSLLHTEPDWQTDYGSTEFGSADFGSADFTSNTDYQDFTQDELAGTLADSMKLHGDPFLNTYVPTTSSSSSGGSYRFAYTCPRHPHGGCACASLFPAHPAHPHQHALDHFVRPPPPADPADIMQTENSTQMVTTSDAALMNGGMMVQTGAMGPTTVMGQIMGALNTGLAEDINIESLEHSFDCNVDEVIKHELSMDGSLDFNFPQQHTAMAAEAESQFAAPAPPVPTTLSGGGQRTPYTVAPSWVH

Yellow: Forkhead (FH) domain found in the Forkhead box protein O (FOXO) subfamily

Green: Transactivation domain of FOXO protein family

**S7.1: Pairwise comparison of gene expression between day 0 and day 3**

| gene | contrast | F | logCPM | logFC | PValue | FDR |
| --- | --- | --- | --- | --- | --- | --- |
| PKA_3 | Dia000_v_Dia003 | 0 | -0.18485 | 0 | 1 | 1 |
| neverland | Dia000_v_Dia003 | 11.50029 | 2.085243 | -2.79166 | 0.001149 | 0.017237 |
| spook | Dia000_v_Dia003 | 0.208273 | 3.264799 | -0.41347 | 0.649571 | 0.941317 |
| FOXO3_2 | Dia000_v_Dia003 | 0 | -2.17379 | 0 | 1 | 1 |
| FOXO | Dia000_v_Dia003 | 0.010881 | -0.36561 | 0.07002 | 0.917202 | 1 |
| FOXO_x2 | Dia000_v_Dia003 | 6.780308 | 2.988 | -0.81807 | 0.011109 | 0.086115 |
| plk1 | Dia000_v_Dia003 | 42.70964 | 4.393491 | 3.009324 | 6.85E-09 | 1.68E-06 |
| FTZ-F1beta | Dia000_v_Dia003 | 1.302172 | 4.856313 | 0.597847 | 0.257501 | 0.585518 |
| E74 | Dia000_v_Dia003 | 2.148889 | 3.799503 | 0.618635 | 0.146861 | 0.432845 |
| raf | Dia000_v_Dia003 | 4.145282 | 5.182635 | -0.26638 | 0.045289 | 0.218589 |
| E93 | Dia000_v_Dia003 | 3.097511 | 3.587462 | 0.526555 | 0.082497 | 0.312023 |
| ILP2 | Dia000_v_Dia003 | 0.410315 | -0.45365 | -0.71002 | 0.523765 | 0.84556 |
| ultraspiracle | Dia000_v_Dia003 | 4.450754 | 6.431101 | -0.38256 | 0.038228 | 0.19632 |
| frizzled | Dia000_v_Dia003 | 0.028737 | 4.370317 | -0.09289 | 0.865849 | 1 |
| PTP61F | Dia000_v_Dia003 | 3.098584 | 6.467028 | -0.36287 | 0.082445 | 0.311969 |
| PKC_x6 | Dia000_v_Dia003 | 0.718875 | 3.631205 | 0.234509 | 0.399216 | 0.728096 |
| ILP5 | Dia000_v_Dia003 | 1.154056 | -1.68363 | -1.5062 | 0.289326 | 0.616306 |
| ILP1 | Dia000_v_Dia003 | 1.273849 | -1.99729 | -1.50651 | 0.2705 | 0.597751 |
| ILP3 | Dia000_v_Dia003 | 0.042671 | -0.78383 | -0.22353 | 0.83717 | 1 |
| shadow | Dia000_v_Dia003 | 0.513169 | 3.610402 | -0.25824 | 0.475999 | 0.801567 |
| broad | Dia000_v_Dia003 | 7.723068 | 6.147805 | -0.96644 | 0.006895 | 0.062902 |
| CDK2 | Dia000_v_Dia003 | 1.60777 | 3.53979 | 0.456443 | 0.208735 | 0.525459 |
| shroud | Dia000_v_Dia003 | 0.922875 | 3.135549 | -0.68567 | 0.339814 | 0.667474 |
| wnt5 | Dia000_v_Dia003 | 0.136277 | 1.765024 | 0.214593 | 0.713053 | 0.988205 |
| rolled_4 | Dia000_v_Dia003 | 0.0005 | 0.80492 | -0.02898 | 0.982216 | 1 |
| PEPCK1 | Dia000_v_Dia003 | 0 | -2.17379 | 0 | 1 | 1 |
| PECK1_2 | Dia000_v_Dia003 | 5.857195 | -0.98729 | 6.488563 | 0.019543 | 0.12661 |
| JHEH | Dia000_v_Dia003 | 9.27808 | 5.696894 | -1.51683 | 0.003215 | 0.036701 |
| svp | Dia000_v_Dia003 | 3.461329 | 4.351326 | -0.4478 | 0.066747 | 0.275651 |
| E75 | Dia000_v_Dia003 | 1.027264 | 2.14629 | 0.42865 | 0.314066 | 0.641063 |
| E75B | Dia000_v_Dia003 | 2.328267 | 1.485675 | 0.82712 | 0.131257 | 0.407367 |
| axin | Dia000_v_Dia003 | 0.057017 | 4.40964 | -0.05151 | 0.811926 | 1 |
| wnt10b | Dia000_v_Dia003 | 1.260554 | -0.35239 | 1.028123 | 0.265283 | 0.596941 |
| wnt6 | Dia000_v_Dia003 | 2.541089 | 3.062162 | -0.74957 | 0.115131 | 0.380281 |
| wnt_1 | Dia000_v_Dia003 | 0.431754 | 1.572159 | -0.292 | 0.513144 | 0.834887 |
| wnt4 | Dia000_v_Dia003 | 0 | -1.6831 | 0 | 1 | 1 |
| PKA_2 | Dia000_v_Dia003 | 0 | -1.34571 | 0 | 1 | 1 |
| smad1 | Dia000_v_Dia003 | 0.453438 | 3.387542 | 0.19737 | 0.50278 | 0.826775 |
| serotonin_transporter | Dia000_v_Dia003 | 0.874819 | 1.500261 | -0.58868 | 0.352632 | 0.680932 |
| gsk3b | Dia000_v_Dia003 | 2.34546 | 6.266624 | 0.201649 | 0.129863 | 0.405019 |
| phantom | Dia000_v_Dia003 | 9.369394 | 5.313587 | -1.34897 | 0.003071 | 0.035727 |
| ror | Dia000_v_Dia003 | 0.021459 | 5.057047 | -0.03012 | 0.883928 | 1 |
| PKA | Dia000_v_Dia003 | 6.430168 | 6.149637 | 0.529114 | 0.013305 | 0.097267 |
| start | Dia000_v_Dia003 | 0.600977 | 1.02394 | 0.84247 | 0.440647 | 0.768668 |
| smad3 | Dia000_v_Dia003 | 0.004999 | 4.763381 | 0.016744 | 0.94382 | 1 |
| E78 | Dia000_v_Dia003 | 4.883075 | 4.463062 | -1.0612 | 0.030186 | 0.168484 |
| FTZ-F1_x2 | Dia000_v_Dia003 | 0 | -2.17379 | 0 | 1 | 1 |
| FTZ-F1 | Dia000_v_Dia003 | 0.236836 | 3.102244 | -0.20182 | 0.627923 | 0.926155 |
| melatonin_receptor | Dia000_v_Dia003 | 2.345963 | -0.21445 | -1.60509 | 0.129823 | 0.404979 |
| shade | Dia000_v_Dia003 | 3.27391 | 4.292045 | -1.03291 | 0.074443 | 0.296286 |
| dopamine_receptor_2 | Dia000_v_Dia003 | 0.002733 | -0.06586 | 0.045764 | 0.993717 | 1 |
| PKC_x1 | Dia000_v_Dia003 | 3.217872 | 5.010541 | -0.47545 | 0.076876 | 0.301865 |
| dopamine_receptor_1 | Dia000_v_Dia003 | 0.59595 | -0.14593 | -0.81817 | 0.443114 | 0.770729 |
| sodium_dependent_dopamine_transporter | Dia000_v_Dia003 | 0.737387 | 3.852934 | -0.37315 | 0.39324 | 0.721196 |
| ERK_1 | Dia000_v_Dia003 | 0.446967 | 5.00882 | -0.10442 | 0.505835 | 0.829311 |
| EcR | Dia000_v_Dia003 | 2.426706 | 5.575761 | 0.413484 | 0.123501 | 0.394229 |
| Pi3K | Dia000_v_Dia003 | 0.334466 | 5.571557 | 0.072743 | 0.564777 | 0.876995 |
| ERK_2 | Dia000_v_Dia003 | 1.211733 | 3.958467 | 0.290445 | 0.274516 | 0.601987 |
| disembodied | Dia000_v_Dia003 | 0.533246 | 1.311049 | 0.561618 | 0.467524 | 0.794289 |
| PTTH | Dia000_v_Dia003 | 1.183215 | -0.15669 | -1.23752 | 0.280192 | 0.607232 |
| DHR4 | Dia000_v_Dia003 | 8.080292 | 4.598175 | 2.72745 | 0.005785 | 0.055648 |
| PTSP | Dia000_v_Dia003 | 1.127226 | 1.899684 | -0.5957 | 0.291781 | 0.618811 |
| apc | Dia000_v_Dia003 | 1.751811 | 5.227555 | -0.35401 | 0.189677 | 0.500079 |
| krueppel | Dia000_v_Dia003 | 0.991955 | 2.261292 | -0.93817 | 0.322473 | 0.649726 |
| krueppel_2 | Dia000_v_Dia003 | 1.548838 | -2.0328 | -1.49014 | 0.2705 | 0.597751 |
| Insulin_receptor | Dia000_v_Dia003 | 3.922528 | 5.499677 | -0.51247 | 0.051317 | 0.234607 |
| DHR3 | Dia000_v_Dia003 | 0.557423 | 6.851724 | -0.42979 | 0.457681 | 0.784126 |
| ras | Dia000_v_Dia003 | 53.50616 | 6.141927 | 0.714396 | 2.40E-10 | 1.16E-07 |
| rolled_2 | Dia000_v_Dia003 | 9.538792 | 6.115441 | 0.452967 | 0.002822 | 0.033735 |
| TOR_2 | Dia000_v_Dia003 | 0 | -2.17379 | 0 | 1 | 1 |
| TOR | Dia000_v_Dia003 | 0 | -2.17379 | 0 | 1 | 1 |
| ROS | Dia000_v_Dia003 | 2.469798 | 5.219442 | 0.424917 | 0.12027 | 0.38882 |
| mef2 | Dia000_v_Dia003 | 0.047268 | 6.194369 | 0.050776 | 0.828479 | 1 |
| AKT | Dia000_v_Dia003 | 2.925482 | 6.170131 | -0.61867 | 0.091357 | 0.332169 |
| rolled_3 | Dia000_v_Dia003 | 0.254458 | 4.833135 | 0.115763 | 0.615434 | 0.916675 |
| 4E-BP | Dia000_v_Dia003 | 10.48105 | 6.426802 | -0.87543 | 0.001798 | 0.024029 |
| torso | Dia000_v_Dia003 | 22.8392 | 4.542023 | 3.57877 | 8.78E-06 | 0.000403 |
| mTOR | Dia000_v_Dia003 | 0.00793 | 4.995058 | 0.017987 | 0.929281 | 1 |
| npc1 | Dia000_v_Dia003 | 5.925881 | 8.066217 | 0.510871 | 0.017303 | 0.117033 |
| PTEN | Dia000_v_Dia003 | 0.080925 | 3.966237 | 0.062622 | 0.776833 | 1 |
| JHDK | Dia000_v_Dia003 | 0 | 4.120388 | 0 | 1 | 1 |

**S7.2: Pairwise comparison of gene expression between day 3 and day 6**

| gene | contrast | F | logCPM | logFC | PValue | FDR |
| --- | --- | --- | --- | --- | --- | --- |
| PKA_3 | Dia003_v_Dia006 | 1.525719 | -0.18485 | -2.70456 | 0.229862 | 0.672836 |
| neverland | Dia003_v_Dia006 | 0.069291 | 2.085243 | -0.19603 | 0.793146 | 1 |
| spook | Dia003_v_Dia006 | 3.921169 | 3.264799 | -1.67262 | 0.051707 | 0.357212 |
| FOXO3_2 | Dia003_v_Dia006 | 0 | -2.17379 | 0 | 1 | 1 |
| FOXO | Dia003_v_Dia006 | 3.35371 | -0.36561 | -1.21665 | 0.071032 | 0.414769 |
| FOXO_x2 | Dia003_v_Dia006 | 9.715083 | 2.988 | -0.96552 | 0.002592 | 0.071553 |
| plk1 | Dia003_v_Dia006 | 4.304675 | 4.393491 | 0.959163 | 0.041445 | 0.326977 |
| FTZ-F1beta | Dia003_v_Dia006 | 0.267231 | 4.856313 | -0.27056 | 0.606739 | 0.965227 |
| E74 | Dia003_v_Dia006 | 0.662414 | 3.799503 | -0.34276 | 0.41829 | 0.830972 |
| raf | Dia003_v_Dia006 | 5.364853 | 5.182635 | -0.30213 | 0.023286 | 0.242351 |
| E93 | Dia003_v_Dia006 | 0.105434 | 3.587462 | 0.097323 | 0.746309 | 1 |
| ILP2 | Dia003_v_Dia006 | 5.44394 | -0.45365 | 2.960073 | 0.022324 | 0.238343 |
| ultraspiracle | Dia003_v_Dia006 | 0.646028 | 6.431101 | -0.14539 | 0.424081 | 0.8361 |
| frizzled | Dia003_v_Dia006 | 2.823477 | 4.370317 | 0.93374 | 0.097102 | 0.471645 |
| PTP61F | Dia003_v_Dia006 | 1.781991 | 6.467028 | 0.274908 | 0.18595 | 0.623657 |
| PKC_x6 | Dia003_v_Dia006 | 0.085874 | 3.631205 | -0.081 | 0.770301 | 1 |
| ILP5 | Dia003_v_Dia006 | 0.0003 | -1.68363 | -0.01681 | 0.986263 | 1 |
| ILP1 | Dia003_v_Dia006 | 0.000318 | -1.99729 | -0.01623 | 0.985861 | 1 |
| ILP3 | Dia003_v_Dia006 | 0.118128 | -0.78383 | -0.3626 | 0.732491 | 1 |
| shadow | Dia003_v_Dia006 | 0.124093 | 3.610402 | 0.126524 | 0.725627 | 1 |
| broad | Dia003_v_Dia006 | 3.822509 | 6.147805 | -0.67434 | 0.054315 | 0.366749 |
| CDK2 | Dia003_v_Dia006 | 0.970963 | 3.53979 | -0.35437 | 0.327614 | 0.752208 |
| shroud | Dia003_v_Dia006 | 0.064869 | 3.135549 | 0.180073 | 0.799658 | 1 |
| wnt5 | Dia003_v_Dia006 | 6.741501 | 1.765024 | 1.564413 | 0.011333 | 0.1665 |
| rolled_4 | Dia003_v_Dia006 | 0.17953 | 0.80492 | -0.53151 | 0.672993 | 1 |
| PEPCK1 | Dia003_v_Dia006 | 0 | -2.17379 | 0 | 1 | 1 |
| PECK1_2 | Dia003_v_Dia006 | 3.530763 | -0.98729 | -4.90921 | 0.066618 | 0.403739 |
| JHEH | Dia003_v_Dia006 | 0.528256 | 5.696894 | 0.344556 | 0.469639 | 0.870237 |
| svp | Dia003_v_Dia006 | 0.29684 | 4.351326 | 0.129778 | 0.58749 | 0.952157 |
| E75 | Dia003_v_Dia006 | 5.017435 | 2.14629 | -0.94809 | 0.028061 | 0.268291 |
| E75B | Dia003_v_Dia006 | 0.013046 | 1.485675 | -0.06202 | 0.90937 | 1 |
| axin | Dia003_v_Dia006 | 2.224089 | 4.40964 | 0.322203 | 0.140074 | 0.554133 |
| wnt10b | Dia003_v_Dia006 | 1.623013 | -0.35239 | 1.237686 | 0.206777 | 0.651335 |
| wnt6 | Dia003_v_Dia006 | 0.001212 | 3.062162 | 0.016104 | 0.972326 | 1 |
| wnt_1 | Dia003_v_Dia006 | 0.039016 | 1.572159 | -0.0871 | 0.843951 | 1 |
| wnt4 | Dia003_v_Dia006 | 0 | -1.6831 | 0 | 1 | 1 |
| PKA_2 | Dia003_v_Dia006 | 0 | -1.34571 | 0 | 1 | 1 |
| smad1 | Dia003_v_Dia006 | 1.289412 | 3.387542 | -0.33219 | 0.259778 | 0.694107 |
| serotonin_transporter | Dia003_v_Dia006 | 0.971762 | 1.500261 | 0.619646 | 0.327416 | 0.752189 |
| gsk3b | Dia003_v_Dia006 | 1.001049 | 6.266624 | -0.13167 | 0.32028 | 0.744844 |
| phantom | Dia003_v_Dia006 | 0.073643 | 5.313587 | -0.11643 | 0.786858 | 1 |
| ror | Dia003_v_Dia006 | 1.790551 | 5.057047 | -0.27469 | 0.184909 | 0.623163 |
| PKA | Dia003_v_Dia006 | 5.597393 | 6.149637 | 0.495418 | 0.020576 | 0.229439 |
| start | Dia003_v_Dia006 | 0.2312 | 1.02394 | 0.526723 | 0.632038 | 0.980421 |
| smad3 | Dia003_v_Dia006 | 2.616035 | 4.763381 | 0.384079 | 0.109994 | 0.496645 |
| E78 | Dia003_v_Dia006 | 0.0377 | 4.463062 | -0.09 | 0.846574 | 1 |
| FTZ-F1_x2 | Dia003_v_Dia006 | 0 | -2.17379 | 0 | 1 | 1 |
| FTZ-F1 | Dia003_v_Dia006 | 1.76425 | 3.102244 | -0.54857 | 0.18813 | 0.626339 |
| melatonin_receptor | Dia003_v_Dia006 | 0.041631 | -0.21445 | 0.199824 | 0.838878 | 1 |
| shade | Dia003_v_Dia006 | 0.440443 | 4.292045 | -0.37293 | 0.508967 | 0.89986 |
| dopamine_receptor_2 | Dia003_v_Dia006 | 0.343712 | -0.06586 | -0.5079 | 0.559793 | 0.93413 |
| PKC_x1 | Dia003_v_Dia006 | 0.375343 | 5.010541 | -0.16116 | 0.541962 | 0.921897 |
| dopamine_receptor_1 | Dia003_v_Dia006 | 0.628393 | -0.14593 | 0.830668 | 0.431024 | 0.840838 |
| sodium_dependent_dopamine_transporter | Dia003_v_Dia006 | 14.64686 | 3.852934 | 1.716583 | 0.000267 | 0.01814 |
| ERK_1 | Dia003_v_Dia006 | 0.536428 | 5.00882 | -0.11411 | 0.466203 | 0.867388 |
| EcR | Dia003_v_Dia006 | 0.587276 | 5.575761 | 0.203505 | 0.445884 | 0.851142 |
| Pi3K | Dia003_v_Dia006 | 7.497563 | 5.571557 | -0.34411 | 0.007716 | 0.134731 |
| ERK_2 | Dia003_v_Dia006 | 0.279737 | 3.958467 | -0.13941 | 0.598438 | 0.959289 |
| disembodied | Dia003_v_Dia006 | 0.740147 | 1.311049 | 0.67204 | 0.39236 | 0.809372 |
| PTTH | Dia003_v_Dia006 | 0.290442 | -0.15669 | 0.582255 | 0.591536 | 0.954361 |
| DHR4 | Dia003_v_Dia006 | 0.644743 | 4.598175 | -0.90304 | 0.424579 | 0.8361 |
| PTSP | Dia003_v_Dia006 | 6.815968 | 1.899684 | 1.511049 | 0.010908 | 0.16371 |
| apc | Dia003_v_Dia006 | 0.774753 | 5.227555 | 0.235013 | 0.381567 | 0.799542 |
| krueppel | Dia003_v_Dia006 | 3.862155 | 2.261292 | -1.55595 | 0.053097 | 0.3618 |
| krueppel_2 | Dia003_v_Dia006 | 0.000846 | -2.0328 | -0.03257 | 0.97703 | 1 |
| Insulin_receptor | Dia003_v_Dia006 | 9.135737 | 5.499677 | -0.7802 | 0.00343 | 0.083651 |
| DHR3 | Dia003_v_Dia006 | 1.03476 | 6.851724 | 0.58638 | 0.312378 | 0.738957 |
| ras | Dia003_v_Dia006 | 14.45015 | 6.141927 | 0.371304 | 0.000291 | 0.018786 |
| rolled_2 | Dia003_v_Dia006 | 0.000832 | 6.115441 | 0.004225 | 0.977072 | 1 |
| TOR_2 | Dia003_v_Dia006 | 0 | -2.17379 | 0 | 1 | 1 |
| TOR | Dia003_v_Dia006 | 0 | -2.17379 | 0 | 1 | 1 |
| ROS | Dia003_v_Dia006 | 2.40195 | 5.219442 | 0.420976 | 0.125401 | 0.526652 |
| mef2 | Dia003_v_Dia006 | 0.321927 | 6.194369 | 0.13253 | 0.572149 | 0.942542 |
| AKT | Dia003_v_Dia006 | 0.510121 | 6.170131 | 0.257074 | 0.477321 | 0.87525 |
| rolled_3 | Dia003_v_Dia006 | 7.640693 | 4.833135 | 0.638756 | 0.00718 | 0.128463 |
| 4E-BP | Dia003_v_Dia006 | 1.661138 | 6.426802 | 0.345311 | 0.201418 | 0.64285 |
| torso | Dia003_v_Dia006 | 1.956494 | 4.542023 | -0.96858 | 0.166089 | 0.593542 |
| mTOR | Dia003_v_Dia006 | 4.798692 | 4.995058 | -0.44211 | 0.031594 | 0.286806 |
| npc1 | Dia003_v_Dia006 | 0.138761 | 8.066217 | -0.07799 | 0.710568 | 1 |
| PTEN | Dia003_v_Dia006 | 0.541925 | 3.966237 | 0.162171 | 0.463936 | 0.866011 |
| JHDK | Dia003_v_Dia006 | 0 | 4.120388 | 0 | 1 | 1 |

**S7.3: Pairwise comparison of gene expression between day 6 and day 24**

| gene | contrast | F | logCPM | logFC | PValue | FDR |
| --- | --- | --- | --- | --- | --- | --- |
| PKA_3 | Dia006_v_Dia024 | 1.494364 | -0.18485 | 2.704559 | 0.234552 | 0.54563 |
| neverland | Dia006_v_Dia024 | 2.677441 | 2.085243 | -1.22931 | 0.106276 | 0.344732 |
| spook | Dia006_v_Dia024 | 0.359208 | 3.264799 | -0.47465 | 0.550927 | 0.849475 |
| FOXO3_2 | Dia006_v_Dia024 | 0 | -2.17379 | 0 | 1 | 1 |
| FOXO | Dia006_v_Dia024 | 0.5207 | -0.36561 | -0.46592 | 0.472791 | 0.783649 |
| FOXO_x2 | Dia006_v_Dia024 | 3.909718 | 2.988 | -0.60525 | 0.051689 | 0.218244 |
| plk1 | Dia006_v_Dia024 | 0.000675 | 4.393491 | 0.012306 | 0.979338 | 1 |
| FTZ-F1beta | Dia006_v_Dia024 | 0.096711 | 4.856313 | -0.16239 | 0.756688 | 0.996901 |
| E74 | Dia006_v_Dia024 | 0.734645 | 3.799503 | 0.361217 | 0.394117 | 0.710758 |
| raf | Dia006_v_Dia024 | 32.99319 | 5.182635 | 0.754484 | 1.87E-07 | 1.23E-05 |
| E93 | Dia006_v_Dia024 | 0.622406 | 3.587462 | -0.23653 | 0.432646 | 0.748815 |
| ILP2 | Dia006_v_Dia024 | 4.35232 | -0.45365 | -2.66376 | 0.040365 | 0.184976 |
| ultraspiracle | Dia006_v_Dia024 | 0.290089 | 6.431101 | -0.0974 | 0.591761 | 0.882812 |
| frizzled | Dia006_v_Dia024 | 0.003684 | 4.370317 | -0.03387 | 0.951763 | 1 |
| PTP61F | Dia006_v_Dia024 | 0.062883 | 6.467028 | -0.05163 | 0.802682 | 1 |
| PKC_x6 | Dia006_v_Dia024 | 7.435796 | 3.631205 | 0.765161 | 0.00796 | 0.058872 |
| ILP5 | Dia006_v_Dia024 | 0.747948 | -1.68363 | -1.27953 | 0.392433 | 0.709043 |
| ILP1 | Dia006_v_Dia024 | 0.313603 | -1.99729 | -0.81158 | 0.578611 | 0.872913 |
| ILP3 | Dia006_v_Dia024 | 0.15544 | -0.78383 | 0.417756 | 0.695038 | 0.956021 |
| shadow | Dia006_v_Dia024 | 0.001408 | 3.610402 | 0.013511 | 0.970165 | 1 |
| broad | Dia006_v_Dia024 | 0.626239 | 6.147805 | -0.27167 | 0.431247 | 0.748111 |
| CDK2 | Dia006_v_Dia024 | 0.075375 | 3.53979 | 0.098548 | 0.784422 | 1 |
| shroud | Dia006_v_Dia024 | 1.456459 | 3.135549 | 0.866979 | 0.231294 | 0.545441 |
| wnt5 | Dia006_v_Dia024 | 0.479123 | 1.765024 | -0.41464 | 0.490961 | 0.798783 |
| rolled_4 | Dia006_v_Dia024 | 0.067494 | 0.80492 | -0.32039 | 0.795735 | 1 |
| PEPCK1 | Dia006_v_Dia024 | 0 | -2.17379 | 0 | 1 | 1 |
| PECK1_2 | Dia006_v_Dia024 | 1.964995 | -0.98729 | 3.339774 | 0.167727 | 0.450192 |
| JHEH | Dia006_v_Dia024 | 6.988507 | 5.696894 | 1.321955 | 0.010013 | 0.069674 |
| svp | Dia006_v_Dia024 | 4.602953 | 4.351326 | -0.51001 | 0.035159 | 0.168219 |
| E75 | Dia006_v_Dia024 | 1.038562 | 2.14629 | -0.42513 | 0.311438 | 0.624403 |
| E75B | Dia006_v_Dia024 | 3.205101 | 1.485675 | -0.96854 | 0.077452 | 0.283175 |
| axin | Dia006_v_Dia024 | 7.35374 | 4.40964 | -0.58659 | 0.008297 | 0.060952 |
| wnt10b | Dia006_v_Dia024 | 0.954007 | -0.35239 | -0.9599 | 0.331978 | 0.646696 |
| wnt6 | Dia006_v_Dia024 | 2.131024 | 3.062162 | -0.67512 | 0.148528 | 0.419895 |
| wnt_1 | Dia006_v_Dia024 | 0.147048 | 1.572159 | -0.16904 | 0.750486 | 0.993363 |
| wnt4 | Dia006_v_Dia024 | 0 | -1.6831 | 0 | 1 | 1 |
| PKA_2 | Dia006_v_Dia024 | 7.903688 | -1.34571 | -5.26891 | 0.007129 | 0.054467 |
| smad1 | Dia006_v_Dia024 | 1.344475 | 3.387542 | -0.33787 | 0.249931 | 0.559183 |
| serotonin_transporter | Dia006_v_Dia024 | 2.987574 | 1.500261 | 1.117889 | 0.088028 | 0.30632 |
| gsk3b | Dia006_v_Dia024 | 1.194629 | 6.266624 | -0.14377 | 0.277902 | 0.586128 |
| phantom | Dia006_v_Dia024 | 0.0006 | 5.313587 | -0.01051 | 0.980517 | 1 |
| ror | Dia006_v_Dia024 | 1.219629 | 5.057047 | -0.22625 | 0.27297 | 0.581038 |
| PKA | Dia006_v_Dia024 | 0.94303 | 6.149637 | -0.20332 | 0.334627 | 0.648706 |
| start | Dia006_v_Dia024 | 0.039608 | 1.02394 | 0.219867 | 0.842789 | 1 |
| smad3 | Dia006_v_Dia024 | 4.981546 | 4.763381 | -0.53045 | 0.028611 | 0.145141 |
| E78 | Dia006_v_Dia024 | 0.130451 | 4.463062 | 0.167824 | 0.718985 | 0.972901 |
| FTZ-F1_x2 | Dia006_v_Dia024 | 0 | -2.17379 | 0 | 1 | 1 |
| FTZ-F1 | Dia006_v_Dia024 | 1.524146 | 3.102244 | -0.50741 | 0.220854 | 0.530466 |
| melatonin_receptor | Dia006_v_Dia024 | 0.090905 | -0.21445 | -0.29526 | 0.763866 | 1 |
| shade | Dia006_v_Dia024 | 0.942752 | 4.292045 | 0.546996 | 0.334728 | 0.648773 |
| dopamine_receptor_2 | Dia006_v_Dia024 | 0.886896 | -0.06586 | 0.827359 | 0.349925 | 0.665 |
| PKC_x1 | Dia006_v_Dia024 | 6.409203 | 5.010541 | -0.66649 | 0.01345 | 0.086523 |
| dopamine_receptor_1 | Dia006_v_Dia024 | 1.254139 | -0.14593 | 1.26719 | 0.267163 | 0.575883 |
| sodium_dependent_dopamine_transporter | Dia006_v_Dia024 | 0.452274 | 3.852934 | -0.2969 | 0.503328 | 0.809004 |
| ERK_1 | Dia006_v_Dia024 | 1.859753 | 5.00882 | 0.212791 | 0.17674 | 0.463115 |
| EcR | Dia006_v_Dia024 | 1.028433 | 5.575761 | -0.26942 | 0.313793 | 0.62671 |
| Pi3K | Dia006_v_Dia024 | 6.325323 | 5.571557 | -0.31539 | 0.014048 | 0.089344 |
| ERK_2 | Dia006_v_Dia024 | 7.054706 | 3.958467 | -0.69877 | 0.009656 | 0.067847 |
| disembodied | Dia006_v_Dia024 | 0.026115 | 1.311049 | 0.127374 | 0.872055 | 1 |
| PTTH | Dia006_v_Dia024 | 1.329991 | -0.15669 | -1.23399 | 0.252475 | 0.561515 |
| DHR4 | Dia006_v_Dia024 | 2.254031 | 4.598175 | 1.94978 | 0.137537 | 0.402583 |
| PTSP | Dia006_v_Dia024 | 0.073332 | 1.899684 | 0.159936 | 0.78729 | 1 |
| apc | Dia006_v_Dia024 | 1.636469 | 5.227555 | -0.34175 | 0.204761 | 0.507935 |
| krueppel | Dia006_v_Dia024 | 4.536329 | 2.261292 | 1.693946 | 0.036469 | 0.172133 |
| krueppel_2 | Dia006_v_Dia024 | 1.770179 | -2.0328 | 1.522705 | 0.234405 | 0.545441 |
| Insulin_receptor | Dia006_v_Dia024 | 6.30229 | 5.499677 | -0.64485 | 0.014217 | 0.09015 |
| DHR3 | Dia006_v_Dia024 | 0.023948 | 6.851724 | 0.089975 | 0.877442 | 1 |
| ras | Dia006_v_Dia024 | 4.222105 | 6.141927 | -0.20073 | 0.04339 | 0.19391 |
| rolled_2 | Dia006_v_Dia024 | 17.59594 | 6.115441 | -0.61575 | 7.42E-05 | 0.001543 |
| TOR_2 | Dia006_v_Dia024 | 0 | -2.17379 | 0 | 1 | 1 |
| TOR | Dia006_v_Dia024 | 0 | -2.17379 | 0 | 1 | 1 |
| ROS | Dia006_v_Dia024 | 0.370088 | 5.219442 | 0.165862 | 0.544798 | 0.844806 |
| mef2 | Dia006_v_Dia024 | 11.4125 | 6.194369 | -0.79238 | 0.00116 | 0.014005 |
| AKT | Dia006_v_Dia024 | 0.523398 | 6.170131 | -0.26049 | 0.471664 | 0.782603 |
| rolled_3 | Dia006_v_Dia024 | 0.078981 | 4.833135 | -0.06497 | 0.779458 | 1 |
| 4E-BP | Dia006_v_Dia024 | 1.045741 | 6.426802 | 0.274518 | 0.309783 | 0.622521 |
| torso | Dia006_v_Dia024 | 0.025004 | 4.542023 | -0.10782 | 0.87479 | 1 |
| mTOR | Dia006_v_Dia024 | 3.22974 | 4.995058 | 0.36251 | 0.076345 | 0.280554 |
| npc1 | Dia006_v_Dia024 | 0.898531 | 8.066217 | -0.1985 | 0.346226 | 0.660834 |
| PTEN | Dia006_v_Dia024 | 2.293716 | 3.966237 | -0.33371 | 0.134109 | 0.396613 |
| JHDK | Dia006_v_Dia024 | 0 | 4.120388 | 0 | 1 | 1 |

**S7.4: Pairwise comparison of gene expression between day 24 and day 114**

| gene | contrast | F | logCPM | logFC | PValue | FDR |
| --- | --- | --- | --- | --- | --- | --- |
| PKA_3 | Dia024_v_Dia114 | 1.149497 | -0.18485 | -2.37393 | 0.295344 | 0.684011 |
| neverland | Dia024_v_Dia114 | 4.763234 | 2.085243 | 1.663751 | 0.032438 | 0.23234 |
| spook | Dia024_v_Dia114 | 3.382198 | 3.264799 | 1.531056 | 0.070245 | 0.351954 |
| FOXO3_2 | Dia024_v_Dia114 | 0 | -2.17379 | 0 | 1 | 1 |
| FOXO | Dia024_v_Dia114 | 4.314113 | -0.36561 | 1.380896 | 0.041228 | 0.265837 |
| FOXO_x2 | Dia024_v_Dia114 | 10.65468 | 2.988 | 1.007503 | 0.001656 | 0.038053 |
| plk1 | Dia024_v_Dia114 | 2.665949 | 4.393491 | -0.76125 | 0.106716 | 0.437099 |
| FTZ-F1beta | Dia024_v_Dia114 | 0.502902 | 4.856313 | -0.36957 | 0.480459 | 0.849584 |
| E74 | Dia024_v_Dia114 | 3.564605 | 3.799503 | 0.805395 | 0.062898 | 0.33214 |
| raf | Dia024_v_Dia114 | 1.482991 | 5.182635 | -0.15965 | 0.227131 | 0.623655 |
| E93 | Dia024_v_Dia114 | 1.433213 | 3.587462 | -0.35762 | 0.235018 | 0.633282 |
| ILP2 | Dia024_v_Dia114 | 2.928378 | -0.45365 | -1.86867 | 0.091175 | 0.405774 |
| ultraspiracle | Dia024_v_Dia114 | 1.696578 | 6.431101 | 0.235727 | 0.196729 | 0.584324 |
| frizzled | Dia024_v_Dia114 | 0.088111 | 4.370317 | -0.16539 | 0.767422 | 1 |
| PTP61F | Dia024_v_Dia114 | 2.087763 | 6.467028 | -0.29764 | 0.152656 | 0.516497 |
| PKC_x6 | Dia024_v_Dia114 | 2.26814 | 3.631205 | 0.430746 | 0.136266 | 0.488356 |
| ILP5 | Dia024_v_Dia114 | 0.119328 | -1.68363 | -0.4736 | 0.731627 | 1 |
| ILP1 | Dia024_v_Dia114 | 0.302641 | -1.99729 | 0.812939 | 0.605829 | 0.939709 |
| ILP3 | Dia024_v_Dia114 | 0.131097 | -0.78383 | 0.393245 | 0.718797 | 1 |
| shadow | Dia024_v_Dia114 | 4.757791 | 3.610402 | -0.78547 | 0.032306 | 0.23185 |
| broad | Dia024_v_Dia114 | 3.329803 | 6.147805 | 0.628731 | 0.072039 | 0.356382 |
| CDK2 | Dia024_v_Dia114 | 3.566215 | 3.53979 | 0.687963 | 0.06284 | 0.33214 |
| shroud | Dia024_v_Dia114 | 0.320483 | 3.135549 | 0.410501 | 0.573011 | 0.920873 |
| wnt5 | Dia024_v_Dia114 | 3.143374 | 1.765024 | 1.09126 | 0.080303 | 0.378701 |
| rolled_4 | Dia024_v_Dia114 | 0.133258 | 0.80492 | 0.452597 | 0.716107 | 1 |
| PEPCK1 | Dia024_v_Dia114 | 0 | -2.17379 | 0 | 1 | 1 |
| PECK1_2 | Dia024_v_Dia114 | 0.437894 | -0.98729 | 1.569436 | 0.511459 | 0.875191 |
| JHEH | Dia024_v_Dia114 | 2.354131 | 5.696894 | -0.7729 | 0.129226 | 0.48096 |
| svp | Dia024_v_Dia114 | 3.772359 | 4.351326 | 0.462006 | 0.055868 | 0.311961 |
| E75 | Dia024_v_Dia114 | 3.41521 | 2.14629 | 0.776329 | 0.068548 | 0.347577 |
| E75B | Dia024_v_Dia114 | 1.162554 | 1.485675 | 0.578537 | 0.284398 | 0.674412 |
| axin | Dia024_v_Dia114 | 1.716394 | 4.40964 | -0.28151 | 0.194163 | 0.580478 |
| wnt10b | Dia024_v_Dia114 | 0.003677 | -0.35239 | 0.05804 | 0.996078 | 1 |
| wnt6 | Dia024_v_Dia114 | 0.759768 | 3.062162 | 0.401598 | 0.386188 | 0.771988 |
| wnt_1 | Dia024_v_Dia114 | 2.036709 | 1.572159 | 0.635657 | 0.157699 | 0.524125 |
| wnt4 | Dia024_v_Dia114 | 1.213436 | -1.6831 | -1.558 | 0.276527 | 0.667265 |
| PKA_2 | Dia024_v_Dia114 | 2.969452 | -1.34571 | 2.759381 | 0.091305 | 0.406133 |
| smad1 | Dia024_v_Dia114 | 0.933548 | 3.387542 | -0.28067 | 0.337053 | 0.727012 |
| serotonin_transporter | Dia024_v_Dia114 | 0.991809 | 1.500261 | 0.664941 | 0.322509 | 0.711685 |
| gsk3b | Dia024_v_Dia114 | 0.46113 | 6.266624 | 0.08933 | 0.49919 | 0.865519 |
| phantom | Dia024_v_Dia114 | 1.459266 | 5.313587 | -0.51889 | 0.230883 | 0.628262 |
| ror | Dia024_v_Dia114 | 0.001064 | 5.057047 | 0.006677 | 0.974063 | 1 |
| PKA | Dia024_v_Dia114 | 0.528861 | 6.149637 | -0.15204 | 0.469354 | 0.842461 |
| start | Dia024_v_Dia114 | 0.054295 | 1.02394 | -0.25718 | 0.816387 | 1 |
| smad3 | Dia024_v_Dia114 | 0.164075 | 4.763381 | -0.09577 | 0.686588 | 0.991865 |
| E78 | Dia024_v_Dia114 | 4.693263 | 4.463062 | 1.041603 | 0.033472 | 0.236298 |
| FTZ-F1_x2 | Dia024_v_Dia114 | 0 | -2.17379 | 0 | 1 | 1 |
| FTZ-F1 | Dia024_v_Dia114 | 0.007988 | 3.102244 | 0.03659 | 0.929023 | 1 |
| melatonin_receptor | Dia024_v_Dia114 | 0.039704 | -0.21445 | 0.195533 | 0.8426 | 1 |
| shade | Dia024_v_Dia114 | 0.01502 | 4.292045 | -0.06898 | 0.902791 | 1 |
| dopamine_receptor_2 | Dia024_v_Dia114 | 5.218946 | -0.06586 | 2.278878 | 0.025729 | 0.204662 |
| PKC_x1 | Dia024_v_Dia114 | 0.021808 | 5.010541 | -0.03862 | 0.882997 | 1 |
| dopamine_receptor_1 | Dia024_v_Dia114 | 1.204744 | -0.14593 | -1.25036 | 0.276699 | 0.667265 |
| sodium_dependent_dopamine_transporter | Dia024_v_Dia114 | 0.789156 | 3.852934 | -0.3905 | 0.377201 | 0.762923 |
| ERK_1 | Dia024_v_Dia114 | 2.029472 | 5.00882 | -0.22247 | 0.158429 | 0.525404 |
| EcR | Dia024_v_Dia114 | 0.295848 | 5.575761 | -0.14428 | 0.588114 | 0.929645 |
| Pi3K | Dia024_v_Dia114 | 0.052506 | 5.571557 | 0.028694 | 0.819383 | 1 |
| ERK_2 | Dia024_v_Dia114 | 0.241812 | 3.958467 | 0.128363 | 0.624339 | 0.951867 |
| disembodied | Dia024_v_Dia114 | 1.281386 | 1.311049 | -0.88855 | 0.261254 | 0.653095 |
| PTTH | Dia024_v_Dia114 | 1.347908 | -0.15669 | -1.18885 | 0.249332 | 0.644384 |
| DHR4 | Dia024_v_Dia114 | 0.508055 | 4.598175 | -1.03935 | 0.478232 | 0.84779 |
| PTSP | Dia024_v_Dia114 | 2.753978 | 1.899684 | 1.039939 | 0.101196 | 0.426294 |
| apc | Dia024_v_Dia114 | 0.800145 | 5.227555 | 0.238828 | 0.373917 | 0.760078 |
| krueppel | Dia024_v_Dia114 | 0.308563 | 2.261292 | -0.47531 | 0.580219 | 0.925313 |
| krueppel_2 | Dia024_v_Dia114 | 0 | -2.0328 | 0 | 1 | 1 |
| Insulin_receptor | Dia024_v_Dia114 | 3.181758 | 5.499677 | 0.457206 | 0.078517 | 0.37468 |
| DHR3 | Dia024_v_Dia114 | 0.058375 | 6.851724 | -0.14066 | 0.809756 | 1 |
| ras | Dia024_v_Dia114 | 0.000796 | 6.141927 | 0.002754 | 0.977563 | 1 |
| rolled_2 | Dia024_v_Dia114 | 2.605737 | 6.115441 | -0.23569 | 0.110685 | 0.44417 |
| TOR_2 | Dia024_v_Dia114 | 0 | -2.17379 | 0 | 1 | 1 |
| TOR | Dia024_v_Dia114 | 0 | -2.17379 | 0 | 1 | 1 |
| ROS | Dia024_v_Dia114 | 3.183388 | 5.219442 | -0.48661 | 0.078442 | 0.374594 |
| mef2 | Dia024_v_Dia114 | 11.32356 | 6.194369 | -0.7864 | 0.00121 | 0.031685 |
| AKT | Dia024_v_Dia114 | 2.627477 | 6.170131 | 0.585979 | 0.109258 | 0.440919 |
| rolled_3 | Dia024_v_Dia114 | 1.636573 | 4.833135 | 0.296811 | 0.204747 | 0.5953 |
| 4E-BP | Dia024_v_Dia114 | 0.108353 | 6.426802 | 0.088497 | 0.742946 | 1 |
| torso | Dia024_v_Dia114 | 4.487187 | 4.542023 | 1.491462 | 0.037526 | 0.251948 |
| mTOR | Dia024_v_Dia114 | 2.709172 | 4.995058 | -0.33216 | 0.103965 | 0.43316 |
| npc1 | Dia024_v_Dia114 | 5.501678 | 8.066217 | -0.49202 | 0.021649 | 0.185469 |
| PTEN | Dia024_v_Dia114 | 0.410142 | 3.966237 | -0.14065 | 0.523853 | 0.883871 |
| JHDK | Dia024_v_Dia114 | 0.687587 | 4.120388 | -1.57585 | 0.410902 | 0.793023 |

**S7.5: Pairwise comparison of gene expression between day 114 and day 144**

| gene | contrast | F | logCPM | logFC | PValue | FDR |
| --- | --- | --- | --- | --- | --- | --- |
| PKA_3 | Dia114_v_Dia144 | 1.204997 | -0.18485 | 2.373926 | 0.284265 | 1 |
| neverland | Dia114_v_Dia144 | 0.132452 | 2.085243 | -0.27217 | 0.717002 | 1 |
| spook | Dia114_v_Dia144 | 2.574243 | 3.264799 | 1.466304 | 0.113224 | 1 |
| FOXO3_2 | Dia114_v_Dia144 | 0 | -2.17379 | 0 | 1 | 1 |
| FOXO | Dia114_v_Dia144 | 0.245821 | -0.36561 | 0.3316 | 0.621486 | 1 |
| FOXO_x2 | Dia114_v_Dia144 | 1.650051 | 2.988 | 0.39559 | 0.202912 | 1 |
| plk1 | Dia114_v_Dia144 | 0.085846 | 4.393491 | -0.13193 | 0.770336 | 1 |
| FTZ-F1beta | Dia114_v_Dia144 | 0.008708 | 4.856313 | 0.048439 | 0.925906 | 1 |
| E74 | Dia114_v_Dia144 | 0.330387 | 3.799503 | 0.245385 | 0.567154 | 1 |
| raf | Dia114_v_Dia144 | 0.223452 | 5.182635 | -0.06185 | 0.637797 | 1 |
| E93 | Dia114_v_Dia144 | 0.431399 | 3.587462 | 0.195537 | 0.513317 | 1 |
| ILP2 | Dia114_v_Dia144 | 0.437712 | -0.45365 | 0.68884 | 0.51026 | 1 |
| ultraspiracle | Dia114_v_Dia144 | 0.449563 | 6.431101 | 0.121331 | 0.504606 | 1 |
| frizzled | Dia114_v_Dia144 | 0.423768 | 4.370317 | -0.36002 | 0.517074 | 1 |
| PTP61F | Dia114_v_Dia144 | 0.318327 | 6.467028 | 0.116078 | 0.574301 | 1 |
| PKC_x6 | Dia114_v_Dia144 | 0.060618 | 3.631205 | 0.070839 | 0.806195 | 1 |
| ILP5 | Dia114_v_Dia144 | 0.269592 | -1.68363 | 0.71399 | 0.606549 | 1 |
| ILP1 | Dia114_v_Dia144 | 1.155698 | -1.99729 | -1.5104 | 0.288822 | 1 |
| ILP3 | Dia114_v_Dia144 | 0.384014 | -0.78383 | -0.66441 | 0.538229 | 1 |
| shadow | Dia114_v_Dia144 | 0.115734 | 3.610402 | -0.12089 | 0.73466 | 1 |
| broad | Dia114_v_Dia144 | 0.175952 | 6.147805 | 0.144103 | 0.676081 | 1 |
| CDK2 | Dia114_v_Dia144 | 2.254866 | 3.53979 | -0.54594 | 0.137401 | 1 |
| shroud | Dia114_v_Dia144 | 1.036254 | 3.135549 | -0.73586 | 0.311973 | 1 |
| wnt5 | Dia114_v_Dia144 | 1.277879 | 1.765024 | -0.69454 | 0.261902 | 1 |
| rolled_4 | Dia114_v_Dia144 | 0.011111 | 0.80492 | -0.13027 | 0.916332 | 1 |
| PEPCK1 | Dia114_v_Dia144 | 0 | -2.17379 | 0 | 1 | 1 |
| PECK1_2 | Dia114_v_Dia144 | 2.099984 | -0.98729 | -3.83927 | 0.154119 | 1 |
| JHEH | Dia114_v_Dia144 | 0.010412 | 5.696894 | -0.0497 | 0.919002 | 1 |
| svp | Dia114_v_Dia144 | 3.03782 | 4.351326 | 0.419313 | 0.085451 | 1 |
| E75 | Dia114_v_Dia144 | 1.605322 | 2.14629 | 0.534674 | 0.209078 | 1 |
| E75B | Dia114_v_Dia144 | 0.004037 | 1.485675 | -0.03419 | 0.971424 | 1 |
| axin | Dia114_v_Dia144 | 0.011702 | 4.40964 | 0.023189 | 0.914144 | 1 |
| wnt10b | Dia114_v_Dia144 | 0.313887 | -0.35239 | 0.540131 | 0.577047 | 1 |
| wnt6 | Dia114_v_Dia144 | 0.062462 | 3.062162 | -0.11508 | 0.80333 | 1 |
| wnt_1 | Dia114_v_Dia144 | 0.402566 | 1.572159 | -0.28213 | 0.527701 | 1 |
| wnt4 | Dia114_v_Dia144 | 0.002475 | -1.6831 | 0.056404 | 0.96054 | 1 |
| PKA_2 | Dia114_v_Dia144 | 0.355057 | -1.34571 | 1.075677 | 0.554071 | 1 |
| smad1 | Dia114_v_Dia144 | 1.273181 | 3.387542 | 0.327752 | 0.262774 | 1 |
| serotonin_transporter | Dia114_v_Dia144 | 0.00209 | 1.500261 | -0.03105 | 0.963656 | 1 |
| gsk3b | Dia114_v_Dia144 | 1.502978 | 6.266624 | 0.161343 | 0.224056 | 1 |
| phantom | Dia114_v_Dia144 | 0.108555 | 5.313587 | -0.14088 | 0.742723 | 1 |
| ror | Dia114_v_Dia144 | 0.000336 | 5.057047 | 0.003749 | 0.985432 | 1 |
| PKA | Dia114_v_Dia144 | 0.792175 | 6.149637 | -0.18568 | 0.376294 | 1 |
| start | Dia114_v_Dia144 | 0.203329 | 1.02394 | -0.49358 | 0.65335 | 1 |
| smad3 | Dia114_v_Dia144 | 2.119297 | 4.763381 | -0.34386 | 0.149634 | 1 |
| E78 | Dia114_v_Dia144 | 0.004918 | 4.463062 | -0.03412 | 0.944282 | 1 |
| FTZ-F1_x2 | Dia114_v_Dia144 | 0 | -2.17379 | 0 | 1 | 1 |
| FTZ-F1 | Dia114_v_Dia144 | 0.602532 | 3.102244 | 0.318247 | 0.440059 | 1 |
| melatonin_receptor | Dia114_v_Dia144 | 0.36653 | -0.21445 | -0.58999 | 0.546733 | 1 |
| shade | Dia114_v_Dia144 | 0.004883 | 4.292045 | 0.039303 | 0.944481 | 1 |
| dopamine_receptor_2 | Dia114_v_Dia144 | 0.218836 | -0.06586 | 0.502519 | 0.641546 | 1 |
| PKC_x1 | Dia114_v_Dia144 | 0.10151 | 5.010541 | 0.083287 | 0.750912 | 1 |
| dopamine_receptor_1 | Dia114_v_Dia144 | 1.739927 | -0.14593 | 1.519373 | 0.192089 | 1 |
| sodium_dependent_dopamine_transporter | Dia114_v_Dia144 | 0.007581 | 3.852934 | 0.038087 | 0.93085 | 1 |
| ERK_1 | Dia114_v_Dia144 | 0.065013 | 5.00882 | -0.03971 | 0.799441 | 1 |
| EcR | Dia114_v_Dia144 | 0.841705 | 5.575761 | 0.243418 | 0.361856 | 1 |
| Pi3K | Dia114_v_Dia144 | 0.758399 | 5.571557 | 0.109065 | 0.386614 | 1 |
| ERK_2 | Dia114_v_Dia144 | 0.090185 | 3.958467 | 0.078417 | 0.764774 | 1 |
| disembodied | Dia114_v_Dia144 | 0.404671 | 1.311049 | -0.48869 | 0.526627 | 1 |
| PTTH | Dia114_v_Dia144 | 0.381035 | -0.15669 | 0.621502 | 0.538923 | 1 |
| DHR4 | Dia114_v_Dia144 | 0.029104 | 4.598175 | 0.221059 | 0.865006 | 1 |
| PTSP | Dia114_v_Dia144 | 1.549851 | 1.899684 | -0.77957 | 0.21704 | 1 |
| apc | Dia114_v_Dia144 | 1.016165 | 5.227555 | -0.26902 | 0.316677 | 1 |
| krueppel | Dia114_v_Dia144 | 0.050775 | 2.261292 | -0.18317 | 0.822334 | 1 |
| krueppel_2 | Dia114_v_Dia144 | 0 | -2.0328 | 0 | 1 | 1 |
| Insulin_receptor | Dia114_v_Dia144 | 2.958898 | 5.499677 | 0.441484 | 0.089537 | 1 |
| DHR3 | Dia114_v_Dia144 | 0.188864 | 6.851724 | -0.25117 | 0.665136 | 1 |
| ras | Dia114_v_Dia144 | 0.150942 | 6.141927 | 0.03791 | 0.698739 | 1 |
| rolled_2 | Dia114_v_Dia144 | 0.011489 | 6.115441 | -0.01563 | 0.914926 | 1 |
| TOR_2 | Dia114_v_Dia144 | 0 | -2.17379 | 0 | 1 | 1 |
| TOR | Dia114_v_Dia144 | 0 | -2.17379 | 0 | 1 | 1 |
| ROS | Dia114_v_Dia144 | 0.424815 | 5.219442 | 0.176845 | 0.51654 | 1 |
| mef2 | Dia114_v_Dia144 | 0.134912 | 6.194369 | 0.085212 | 0.714429 | 1 |
| AKT | Dia114_v_Dia144 | 0.538296 | 6.170131 | -0.26467 | 0.465445 | 1 |
| rolled_3 | Dia114_v_Dia144 | 3.274499 | 4.833135 | -0.41941 | 0.074378 | 1 |
| 4E-BP | Dia114_v_Dia144 | 2.790057 | 6.426802 | -0.44905 | 0.099027 | 1 |
| torso | Dia114_v_Dia144 | 0.303177 | 4.542023 | 0.38811 | 0.583565 | 1 |
| mTOR | Dia114_v_Dia144 | 1.740028 | 4.995058 | 0.265866 | 0.191156 | 1 |
| npc1 | Dia114_v_Dia144 | 0.007049 | 8.066217 | 0.017565 | 0.933315 | 1 |
| PTEN | Dia114_v_Dia144 | 1.806567 | 3.966237 | 0.29539 | 0.182979 | 1 |
| JHDK | Dia114_v_Dia144 | 0.721347 | 4.120388 | 1.575848 | 0.399725 | 1 |

**S7.6: Pairwise comparison of gene expression between day 144 and day 155**

| gene | contrast | F | logCPM | logFC | PValue | FDR |
| --- | --- | --- | --- | --- | --- | --- |
| PKA_3 | Dia144_v_Dia155 | 0 | -0.18485 | 0 | 1 | 1 |
| neverland | Dia144_v_Dia155 | 0.094267 | 2.085243 | -0.22823 | 0.759734 | 0.947163 |
| spook | Dia144_v_Dia155 | 33.24039 | 3.264799 | -5.56205 | 2.14E-07 | 2.54E-06 |
| FOXO3_2 | Dia144_v_Dia155 | 0 | -2.17379 | 0 | 1 | 1 |
| FOXO | Dia144_v_Dia155 | 3.793075 | -0.36561 | 1.357303 | 0.055215 | 0.131322 |
| FOXO_x2 | Dia144_v_Dia155 | 40.60309 | 2.988 | 2.078349 | 1.37E-08 | 2.16E-07 |
| plk1 | Dia144_v_Dia155 | 45.91963 | 4.393491 | -3.13348 | 2.45E-09 | 4.68E-08 |
| FTZ-F1beta | Dia144_v_Dia155 | 0.065301 | 4.856313 | 0.132577 | 0.799016 | 0.981115 |
| E74 | Dia144_v_Dia155 | 0.515256 | 3.799503 | -0.30556 | 0.475106 | 0.675926 |
| raf | Dia144_v_Dia155 | 2.012378 | 5.182635 | -0.18518 | 0.16017 | 0.30416 |
| E93 | Dia144_v_Dia155 | 2.259936 | 3.587462 | -0.4465 | 0.136966 | 0.26988 |
| ILP2 | Dia144_v_Dia155 | 3.28893 | -0.45365 | 2.025029 | 0.073756 | 0.166238 |
| ultraspiracle | Dia144_v_Dia155 | 51.6652 | 6.431101 | 1.325699 | 4.15E-10 | 9.69E-09 |
| frizzled | Dia144_v_Dia155 | 0.167694 | 4.370317 | -0.22446 | 0.68335 | 0.877815 |
| PTP61F | Dia144_v_Dia155 | 3.76559 | 6.467028 | 0.399916 | 0.056083 | 0.133064 |
| PKC_x6 | Dia144_v_Dia155 | 30.31077 | 3.631205 | -1.56992 | 4.94E-07 | 5.36E-06 |
| ILP5 | Dia144_v_Dia155 | 0.067121 | -1.68363 | -0.34671 | 0.796943 | 0.97923 |
| ILP1 | Dia144_v_Dia155 | 0.454685 | -1.99729 | 0.882509 | 0.50401 | 0.706052 |
| ILP3 | Dia144_v_Dia155 | 0.712986 | -0.78383 | -0.86284 | 0.402408 | 0.596164 |
| shadow | Dia144_v_Dia155 | 0.053573 | 3.610402 | -0.08195 | 0.81759 | 0.997814 |
| broad | Dia144_v_Dia155 | 205.5319 | 6.147805 | 6.54837 | 3.95E-23 | 4.33E-20 |
| CDK2 | Dia144_v_Dia155 | 2.035288 | 3.53979 | -0.51094 | 0.157842 | 0.300746 |
| shroud | Dia144_v_Dia155 | 4.252495 | 3.135549 | 1.521996 | 0.042663 | 0.106436 |
| wnt5 | Dia144_v_Dia155 | 17.64511 | 1.765024 | -2.59286 | 7.27E-05 | 0.000435 |
| rolled_4 | Dia144_v_Dia155 | 0.346909 | 0.80492 | 0.736674 | 0.557641 | 0.761026 |
| PEPCK1 | Dia144_v_Dia155 | 0 | -2.17379 | 0 | 1 | 1 |
| PECK1_2 | Dia144_v_Dia155 | 0.352589 | -0.98729 | 1.282403 | 0.555575 | 0.759151 |
| JHEH | Dia144_v_Dia155 | 1.704574 | 5.696894 | -0.62484 | 0.195745 | 0.35578 |
| svp | Dia144_v_Dia155 | 18.45955 | 4.351326 | -1.02819 | 5.15E-05 | 0.00032 |
| E75 | Dia144_v_Dia155 | 1.461262 | 2.14629 | -0.50876 | 0.230533 | 0.404657 |
| E75B | Dia144_v_Dia155 | 3.298321 | 1.485675 | -0.97117 | 0.073354 | 0.165578 |
| axin | Dia144_v_Dia155 | 5.558357 | 4.40964 | 0.507121 | 0.021007 | 0.058906 |
| wnt10b | Dia144_v_Dia155 | 3.132534 | -0.35239 | -1.6683 | 0.080986 | 0.179123 |
| wnt6 | Dia144_v_Dia155 | 0.61103 | 3.062162 | 0.35993 | 0.436865 | 0.634752 |
| wnt_1 | Dia144_v_Dia155 | 3.902592 | 1.572159 | -0.8724 | 0.051898 | 0.124841 |
| wnt4 | Dia144_v_Dia155 | 1.227651 | -1.6831 | 1.501595 | 0.273769 | 0.449468 |
| PKA_2 | Dia144_v_Dia155 | 0.934257 | -1.34571 | 1.433856 | 0.338617 | 0.523292 |
| smad1 | Dia144_v_Dia155 | 4.062836 | 3.387542 | 0.588838 | 0.047426 | 0.116018 |
| serotonin_transporter | Dia144_v_Dia155 | 2.915794 | 1.500261 | -1.12428 | 0.09186 | 0.19775 |
| gsk3b | Dia144_v_Dia155 | 14.478 | 6.266624 | -0.50106 | 0.000288 | 0.001475 |
| phantom | Dia144_v_Dia155 | 19.94945 | 5.313587 | 1.988745 | 2.80E-05 | 0.000187 |
| ror | Dia144_v_Dia155 | 0.880313 | 5.057047 | 0.191981 | 0.351133 | 0.537333 |
| PKA | Dia144_v_Dia155 | 1.573639 | 6.149637 | -0.26098 | 0.213582 | 0.380835 |
| start | Dia144_v_Dia155 | 0.314095 | 1.02394 | -0.60634 | 0.576852 | 0.77993 |
| smad3 | Dia144_v_Dia155 | 22.26301 | 4.763381 | 1.128993 | 1.08E-05 | 8.08E-05 |
| E78 | Dia144_v_Dia155 | 36.9489 | 4.463062 | -3.01155 | 4.73E-08 | 6.57E-07 |
| FTZ-F1_x2 | Dia144_v_Dia155 | 0 | -2.17379 | 0 | 1 | 1 |
| FTZ-F1 | Dia144_v_Dia155 | 85.16808 | 3.102244 | 4.593134 | 5.56E-14 | 4.22E-12 |
| melatonin_receptor | Dia144_v_Dia155 | 8.312801 | -0.21445 | 3.177895 | 0.005136 | 0.018107 |
| shade | Dia144_v_Dia155 | 1.885999 | 4.292045 | 0.778763 | 0.173793 | 0.324005 |
| dopamine_receptor_2 | Dia144_v_Dia155 | 15.54739 | -0.06586 | -4.05875 | 0.000205 | 0.001094 |
| PKC_x1 | Dia144_v_Dia155 | 27.13247 | 5.010541 | 1.394 | 1.62E-06 | 1.52E-05 |
| dopamine_receptor_1 | Dia144_v_Dia155 | 4.621467 | -0.14593 | -2.44385 | 0.035557 | 0.091473 |
| sodium_dependent_dopamine_transporter | Dia144_v_Dia155 | 3.06573 | 3.852934 | -0.76614 | 0.084055 | 0.184303 |
| ERK_1 | Dia144_v_Dia155 | 2.981828 | 5.00882 | -0.26823 | 0.088328 | 0.191691 |
| EcR | Dia144_v_Dia155 | 6.939907 | 5.575761 | -0.7002 | 0.010238 | 0.032398 |
| Pi3K | Dia144_v_Dia155 | 16.14409 | 5.571557 | 0.504883 | 0.000138 | 0.00077 |
| ERK_2 | Dia144_v_Dia155 | 0.652729 | 3.958467 | -0.21044 | 0.421698 | 0.617622 |
| disembodied | Dia144_v_Dia155 | 0.683113 | 1.311049 | -0.62609 | 0.411143 | 0.606001 |
| PTTH | Dia144_v_Dia155 | 1.327973 | -0.15669 | 1.193053 | 0.252832 | 0.426034 |
| DHR4 | Dia144_v_Dia155 | 78.60965 | 4.598175 | -8.14236 | 3.06E-13 | 1.90E-11 |
| PTSP | Dia144_v_Dia155 | 1.637343 | 1.899684 | -0.74773 | 0.204642 | 0.368127 |
| apc | Dia144_v_Dia155 | 2.974525 | 5.227555 | 0.460629 | 0.088711 | 0.19228 |
| krueppel | Dia144_v_Dia155 | 4.748569 | 2.261292 | 2.021226 | 0.032469 | 0.084721 |
| krueppel_2 | Dia144_v_Dia155 | 0 | -2.0328 | 0 | 1 | 1 |
| Insulin_receptor | Dia144_v_Dia155 | 29.63195 | 5.499677 | 1.428749 | 6.35E-07 | 6.70E-06 |
| DHR3 | Dia144_v_Dia155 | 52.12612 | 6.851724 | -4.64571 | 3.93E-10 | 9.22E-09 |
| ras | Dia144_v_Dia155 | 158.2457 | 6.141927 | -1.23897 | 3.80E-20 | 2.06E-17 |
| rolled_2 | Dia144_v_Dia155 | 1.586588 | 6.115441 | 0.183628 | 0.211727 | 0.378184 |
| TOR_2 | Dia144_v_Dia155 | 0 | -2.17379 | 0 | 1 | 1 |
| TOR | Dia144_v_Dia155 | 0 | -2.17379 | 0 | 1 | 1 |
| ROS | Dia144_v_Dia155 | 1.270988 | 5.219442 | -0.30509 | 0.263182 | 0.437855 |
| mef2 | Dia144_v_Dia155 | 16.3305 | 6.194369 | 0.946828 | 0.000128 | 0.000719 |
| AKT | Dia144_v_Dia155 | 2.259518 | 6.170131 | 0.543377 | 0.137027 | 0.269904 |
| rolled_3 | Dia144_v_Dia155 | 11.03613 | 4.833135 | -0.76626 | 0.001384 | 0.005857 |
| 4E-BP | Dia144_v_Dia155 | 2.925542 | 6.426802 | 0.459243 | 0.091329 | 0.196839 |
| torso | Dia144_v_Dia155 | 0.042802 | 4.542023 | -0.14544 | 0.836671 | 1 |
| mTOR | Dia144_v_Dia155 | 0.754481 | 4.995058 | 0.175091 | 0.387837 | 0.579431 |
| npc1 | Dia144_v_Dia155 | 19.72035 | 8.066217 | 0.937368 | 3.04E-05 | 0.000202 |
| PTEN | Dia144_v_Dia155 | 0.022256 | 3.966237 | 0.032764 | 0.881808 | 1 |
| JHDK | Dia144_v_Dia155 | 0 | 4.120388 | 0 | 1 | 1 |

**8.1 Pairwise comparison for PTTH protein**

| comparison | difference | lwr | upr | p adj |
| --- | --- | --- | --- | --- |
| 3 - 0 | 26.15813 | -30.5203 | 82.8366 | 0.698455 |
| 6 - 0 | 9.6125 | -47.066 | 66.29097 | 0.99649 |
| 24-0 | 7.326423 | -49.352 | 64.00489 | 0.999222 |
| 114-0 | -5.5053 | -62.1838 | 51.17317 | 0.999849 |
| 136-0 | -7.9692 | -64.6477 | 48.70927 | 0.998752 |
| 155-0 | 125.9644 | 69.28593 | 182.6429 | 4.09E-05 |
| 6 - 3 | -16.5456 | -73.2241 | 40.13284 | 0.946529 |
| 24-3 | -18.8317 | -75.5102 | 37.84676 | 0.90669 |
| 114-3 | -31.6634 | -88.3419 | 25.01504 | 0.506514 |
| 136-3 | -34.1273 | -90.8058 | 22.55114 | 0.42538 |
| 155-3 | 99.80627 | 43.1278 | 156.4847 | 0.000493 |
| 24-6 | -2.28608 | -58.9645 | 54.39239 | 0.999999 |
| 114-6 | -15.1178 | -71.7963 | 41.56067 | 0.964652 |
| 136-6 | -17.5817 | -74.2602 | 39.09677 | 0.930161 |
| 155-6 | 116.3519 | 59.67343 | 173.0304 | 9.86E-05 |
| 114-24 | -12.8317 | -69.5102 | 43.84675 | 0.984071 |
| 136-24 | -15.2956 | -71.9741 | 41.38285 | 0.962664 |
| 155-24 | 118.638 | 61.95951 | 175.3164 | 7.97E-05 |
| 136-114 | -2.4639 | -59.1424 | 54.21457 | 0.999999 |
| 155-114 | 131.4697 | 74.79123 | 188.1482 | 2.52E-05 |
| 155-136 | 133.9336 | 77.25513 | 190.6121 | 2.03E-05 |

**8.2 Pairwise comparison for EcR protein**

| comparison | difference | lwr | upr | p adj |
| --- | --- | --- | --- | --- |
| 3-0 | 0.266816 | -0.83622 | 1.369851 | 0.976274 |
| 6-0 | 1.703608 | 0.600573 | 2.806644 | 0.001963 |
| 54-0 | -0.03133 | -1.26456 | 1.201897 | 1 |
| 84-0 | -0.06355 | -1.16659 | 1.039484 | 0.999992 |
| 136-0 | 1.263732 | 0.160696 | 2.366767 | 0.02086 |
| 155-0 | -0.26598 | -1.36902 | 0.837053 | 0.976632 |
| 6-3 | 1.436793 | 0.333757 | 2.539828 | 0.008117 |
| 54-3 | -0.29815 | -1.53138 | 0.935082 | 0.976335 |
| 84-3 | -0.33037 | -1.4334 | 0.772668 | 0.936367 |
| 136-3 | 0.996916 | -0.10612 | 2.099952 | 0.087891 |
| 155-3 | -0.5328 | -1.63583 | 0.570238 | 0.645869 |
| 54-6 | -1.73494 | -2.96817 | -0.50171 | 0.004371 |
| 84-6 | -1.76716 | -2.8702 | -0.66412 | 0.001414 |
| 136-6 | -0.43988 | -1.54291 | 0.663159 | 0.804168 |
| 155-6 | -1.96959 | -3.07263 | -0.86656 | 0.000512 |
| 84-54 | -0.03222 | -1.26545 | 1.201013 | 1 |
| 136-54 | 1.295065 | 0.061834 | 2.528297 | 0.037069 |
| 155-54 | -0.23465 | -1.46788 | 0.998583 | 0.99297 |
| 136-84 | 1.327284 | 0.224248 | 2.430319 | 0.014737 |
| 155-84 | -0.20243 | -1.30547 | 0.900605 | 0.994191 |
| 155-136 | -1.52971 | -2.63275 | -0.42668 | 0.004919 |

**8.3 Pairwise comparison for USP protein**

| comparison | difference | lwr | upr | p adj |
| --- | --- | --- | --- | --- |
| 3 - 0 | -0.61759 | -3.22182 | 1.986649 | 0.9743 |
| 6 -0 | -1.23763 | -3.84187 | 1.3666 | 0.635249 |
| 54-0 | -1.2444 | -4.0972 | 1.608392 | 0.712648 |
| 84-0 | -1.3185 | -4.1713 | 1.534293 | 0.661401 |
| 136-0 | -1.24768 | -3.85191 | 1.356558 | 0.627507 |
| 155-0 | -0.21426 | -2.8185 | 2.389973 | 0.999921 |
| 6 - 3 | -0.62005 | -2.94935 | 1.709249 | 0.95596 |
| 54-3 | -0.62682 | -3.23105 | 1.977415 | 0.972414 |
| 84-3 | -0.70092 | -3.30515 | 1.903317 | 0.953714 |
| 136-3 | -0.63009 | -2.95939 | 1.699207 | 0.952652 |
| 155-3 | 0.403324 | -1.92597 | 2.732622 | 0.994782 |
| 54-6 | -0.00677 | -2.611 | 2.597465 | 1 |
| 84-6 | -0.08087 | -2.6851 | 2.523366 | 1 |
| 136-6 | -0.01004 | -2.33934 | 2.319256 | 1 |
| 155-6 | 1.023373 | -1.30593 | 3.352671 | 0.706528 |
| 84-54 | -0.0741 | -2.92689 | 2.778698 | 1 |
| 136-54 | -0.00327 | -2.60751 | 2.600962 | 1 |
| 155-54 | 1.030143 | -1.57409 | 3.634377 | 0.788501 |
| 136-84 | 0.070826 | -2.53341 | 2.67506 | 1 |
| 155-84 | 1.104241 | -1.49999 | 3.708475 | 0.736092 |
| 155-136 | 1.033415 | -1.29588 | 3.362713 | 0.698091 |

**8.4 Pairwise comparison for the FoxO protein**

| comparison | difference | lwr | upr | p adj |
| --- | --- | --- | --- | --- |
| 3-0 | -93.11 | -289.897 | 103.6771 | 0.75575 |
| 9-0 | -186.873 | -383.66 | 9.913733 | 0.069951 |
| 24-0 | -275.025 | -495.04 | -55.0104 | 0.00878 |
| 54-0 | -258.31 | -455.097 | -61.5229 | 0.005637 |
| 114-0 | -270.42 | -467.207 | -73.6329 | 0.003648 |
| 148-0 | -247.533 | -444.32 | -50.7463 | 0.008306 |
| 152-0 | -260.517 | -457.304 | -63.7296 | 0.005207 |
| 161-0 | -49.74 | -246.527 | 147.0471 | 0.990486 |
| 9-3 | -93.7633 | -290.55 | 103.0237 | 0.749342 |
| 24-3 | -181.915 | -401.93 | 38.09963 | 0.152197 |
| 54-3 | -165.2 | -361.987 | 31.58707 | 0.14095 |
| 114-3 | -177.31 | -374.097 | 19.47707 | 0.095912 |
| 148-3 | -154.423 | -351.21 | 42.36373 | 0.19516 |
| 152-3 | -167.407 | -364.194 | 29.3804 | 0.131585 |
| 161-3 | 43.37 | -153.417 | 240.1571 | 0.996115 |
| 24-9 | -88.1517 | -308.166 | 131.863 | 0.877649 |
| 54-9 | -71.4367 | -268.224 | 125.3504 | 0.923889 |
| 114-9 | -83.5467 | -280.334 | 113.2404 | 0.841819 |
| 148-9 | -60.66 | -257.447 | 136.1271 | 0.968428 |
| 152-9 | -73.6433 | -270.43 | 123.1437 | 0.911469 |
| 161-9 | 137.1333 | -59.6537 | 333.9204 | 0.315072 |
| 54-24 | 16.715 | -203.3 | 236.7296 | 0.999999 |
| 114-24 | 4.605 | -215.41 | 224.6196 | 1 |
| 148-24 | 27.49167 | -192.523 | 247.5063 | 0.999935 |
| 152-24 | 14.50833 | -205.506 | 234.523 | 1 |
| 161-24 | 225.285 | 5.270371 | 445.2996 | 0.042514 |
| 114-54 | -12.11 | -208.897 | 184.6771 | 1 |
| 148-54 | 10.77667 | -186.01 | 207.5637 | 1 |
| 152-54 | -2.20667 | -198.994 | 194.5804 | 1 |
| 161-54 | 208.57 | 11.78293 | 405.3571 | 0.033252 |
| 148-114 | 22.88667 | -173.9 | 219.6737 | 0.999962 |
| 152-114 | 9.903333 | -186.884 | 206.6904 | 1 |
| 161-114 | 220.68 | 23.89293 | 417.4671 | 0.021706 |
| 152-148 | -12.9833 | -209.77 | 183.8037 | 1 |
| 161-148 | 197.7933 | 1.006267 | 394.5804 | 0.048305 |
| 161-152 | 210.7767 | 13.9896 | 407.5637 | 0.03078 |

**8.5 Pairwise comparison for the AKT protein**

| comparison | difference | lwr | upr | p adj |
| --- | --- | --- | --- | --- |
| 3-0 | 15.01453 | -89.4203 | 119.4494 | 0.998586 |
| 6-0 | -99.1883 | -203.623 | 5.246506 | 0.067699 |
| 24-0 | -51.5211 | -155.956 | 52.91377 | 0.635781 |
| 114-0 | -108.579 | -213.014 | -4.14443 | 0.039238 |
| 136-0 | -118.212 | -222.647 | -13.7775 | 0.022193 |
| 155-0 | 80.90593 | -23.5289 | 185.3408 | 0.184234 |
| 6-3 | -114.203 | -218.638 | -9.76803 | 0.028157 |
| 24-3 | -66.5356 | -170.97 | 37.89924 | 0.365216 |
| 114-3 | -123.594 | -228.029 | -19.159 | 0.016108 |
| 136-3 | -133.227 | -237.662 | -28.792 | 0.009073 |
| 155-3 | 65.8914 | -38.5434 | 170.3262 | 0.375443 |
| 24-6 | 47.66727 | -56.7676 | 152.1021 | 0.708279 |
| 114-6 | -9.39093 | -113.826 | 95.04391 | 0.999904 |
| 136-6 | -19.024 | -123.459 | 85.41087 | 0.994844 |
| 155-6 | 180.0943 | 75.65943 | 284.5291 | 0.000608 |
| 114-24 | -57.0582 | -161.493 | 47.37664 | 0.530432 |
| 136-24 | -66.6912 | -171.126 | 37.74361 | 0.36277 |
| 155-24 | 132.427 | 27.99216 | 236.8618 | 0.009516 |
| 136-114 | -9.63303 | -114.068 | 94.80181 | 0.999888 |
| 155-114 | 189.4852 | 85.05036 | 293.92 | 0.000364 |
| 155-136 | 199.1182 | 94.68339 | 303.5531 | 0.000217 |

*
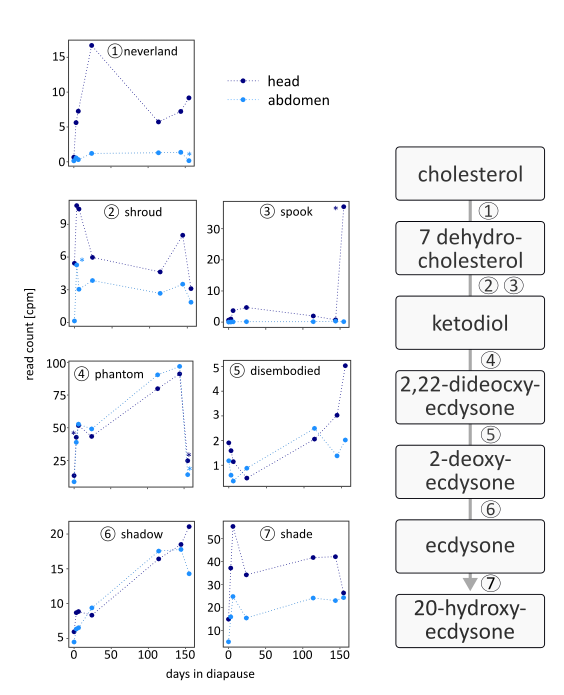
****Figure S9: mRNA patterns in the Halloween genes, the enzymes regulating ecdysone synthesis.*** *The production cascade of ecdysone from cholesterol with the seven P450 enzymes. Expression was quantified in the head (navy blue) and abdomen (light blue). Stars indicate a significant change from the preceding time point.*


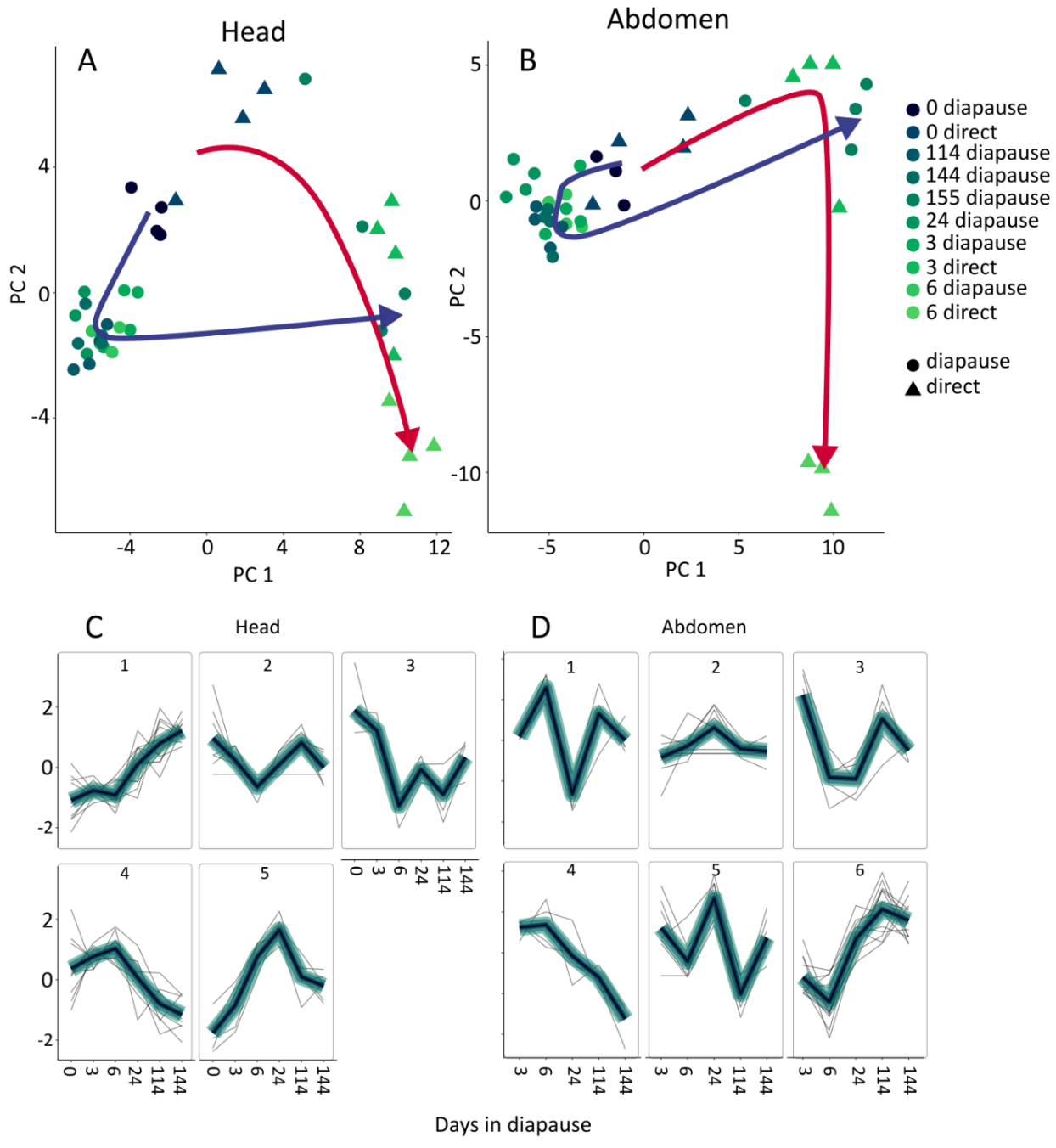
**Figure S10: Principal component analysis (PCA) and fuzzy clustering genes in major hormonal pathways in pupal head and abdomen**. A and B: Principal component analysis (PCA) of the transcriptomic patterns of the ecdysone pathway, insulin, FoxO and some indicators of development throughout diapause in *P. napi*. The blue and red arrows indicate the diapause and direct development trajectories, respectively. PCA in both tissues show the similar patterns as PCA on transcript and whole-gene expression (Pruisscher et al. 2022). C & D: Gene clusters compiled by the minimum centroid distance method with the MFuzz package in R [2].


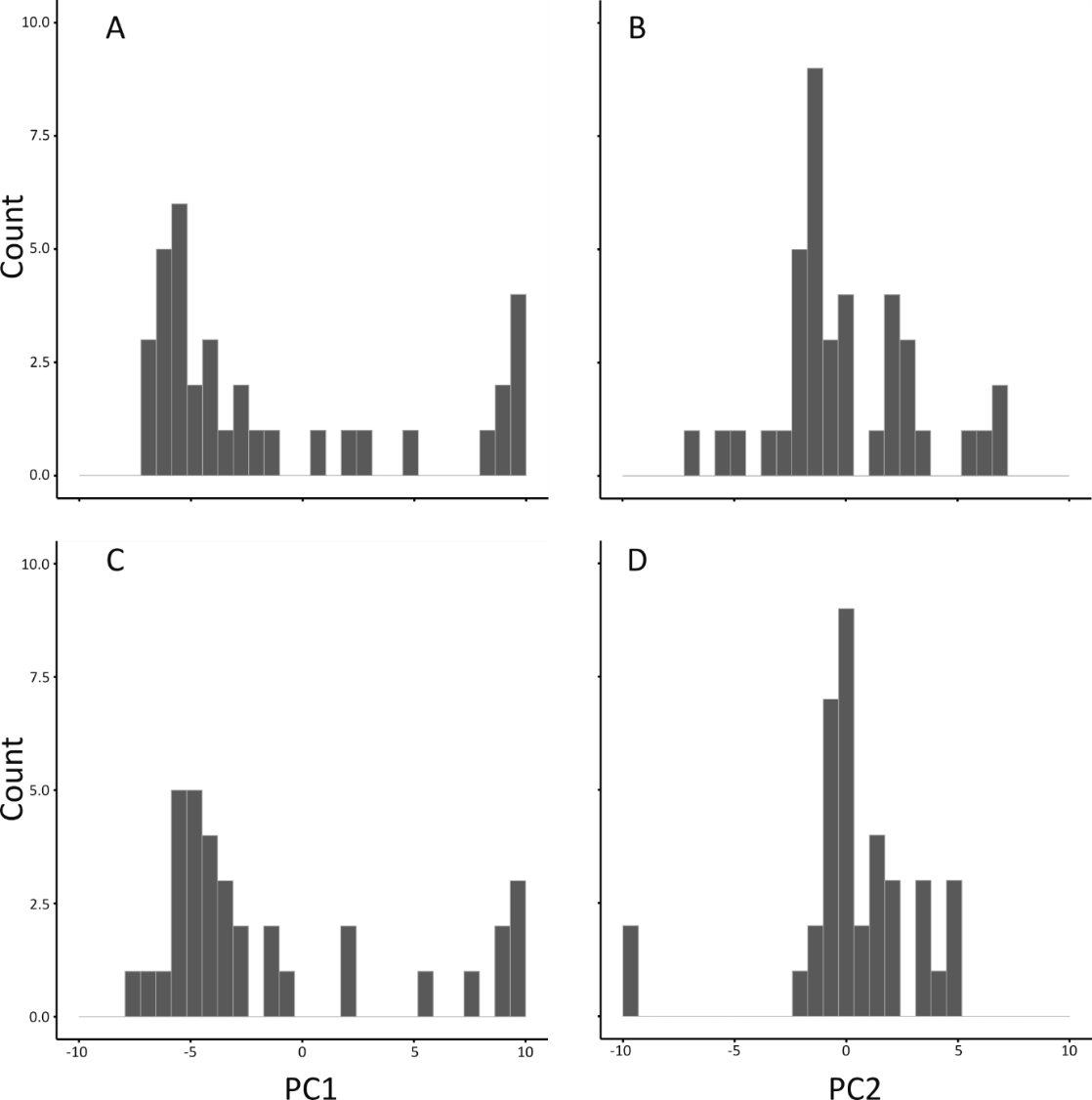
**S11: Histogram of the effect-size of different genes in the head and abdomen in PC1 and PC2.** A: Counts of genes in the hormonal pathways and its effect-size on PC1 in the head, indicating that most genes have a big effect-size on diapause vs developmental differentiation. B: Counts of the genes in the hormonal pathways and its effect-size on PC2 in the head, indication that many genes have a small effect-size and only few are driving development. C: Counts of the genes in the hormonal pathways and its effect-size on PC1 in the abdomen, indicating that most genes have a big effect-size on diapause vs developmental differentiation. D: Counts of genes in the hormonal pathways and its effect size on PC2 in the abdomen, indicating that most genes have a small effect-size driving and only few genes are driving development.

**Table S12.1: logFC, logCPM, F, pvalue, and FDR of the comparisons between diapause initiation (days 0, 3, 6) and endogenous diapause (day 24).** Genes were considered significantly different when the false discovery rate (FDR, Benjamini-Hochberg method) was less than 0.05 and corresponded to genes colored in red (upregulated) and blue (downregulated) in Figures 4 and 7 in the main text.

| **gene** | **logFC** | **logCPM** | **F** | **pvalue** | **FDR** |
| --- | --- | --- | --- | --- | --- |
| neverland | -1.8 | 2.1 | 4.27 | 0.042 | 0.12 |
| spook | -1.25 | 3.29 | 1.33 | 0.252 | 0.426 |
| FOXO | -1.27 | -0.37 | 8.5 | 0.004 | 0.023 |
| FOXO_x2 | -1.45 | 2.99 | 28.2 | 0 | 0 |
| plk1 | 2.26 | 4.41 | 8.6 | 0.004 | 0.022 |
| FTZ-F1beta | -0.28 | 4.87 | 0.51 | 0.478 | 0.647 |
| E74 | 0.42 | 3.8 | 0.93 | 0.338 | 0.519 |
| raf | 0.51 | 5.2 | 13.3 | 0 | 0.004 |
| E93 | -0.1 | 3.6 | 0.1 | 0.754 | 0.856 |
| ILP5 | -0.45 | -0.44 | 0.39 | 0.535 | 0.694 |
| ultraspiracle | -0.4 | 6.44 | 2.6 | 0.11 | 0.242 |
| frizzled | 0.4 | 4.38 | 0.86 | 0.357 | 0.537 |
| PTP61F | 0.09 | 6.48 | 0.1 | 0.751 | 0.855 |
| PKC_x6 | 0.91 | 3.66 | 2.34 | 0.129 | 0.271 |
| ILP3 | 0.67 | -0.77 | 0.38 | 0.537 | 0.695 |
| shadow | -0.37 | 3.62 | 1.73 | 0.192 | 0.356 |
| broad | -0.91 | 6.14 | 3.51 | 0.064 | 0.166 |
| CDK2 | -0.07 | 3.55 | 0.07 | 0.799 | 0.884 |
| shroud | 0.27 | 3.2 | 0.16 | 0.693 | 0.816 |
| wnt5 | 1.31 | 1.78 | 5.53 | 0.021 | 0.073 |
| rolled_4 | -0.57 | -0.31 | 0.55 | 0.461 | 0.633 |
| PECK1_2 | 3.56 | -1.11 | 5.3 | 0.024 | 0.079 |
| JHEH | 0.94 | 5.74 | 2.19 | 0.142 | 0.289 |
| svp | -0.78 | 4.35 | 3.57 | 0.062 | 0.162 |
| E75 | -0.64 | 2.13 | 3.66 | 0.059 | 0.156 |
| E75B | -0.13 | 1.48 | 0.06 | 0.804 | 0.887 |
| axin | -0.39 | 4.42 | 4.21 | 0.043 | 0.123 |
| wnt10b | 0.48 | -0.34 | 0.41 | 0.521 | 0.683 |
| wnt6 | -1.44 | 3.06 | 13.1 | 0 | 0.004 |
| wnt_1 | -0.28 | 1.59 | 0.26 | 0.609 | 0.755 |
| smad1 | -0.67 | 3.39 | 9.26 | 0.003 | 0.017 |
| serotonin trans | 1.39 | 1.52 | 2.98 | 0.088 | 0.206 |
| gsk3b | -0.2 | 6.28 | 0.62 | 0.431 | 0.606 |
| phantom | -0.47 | 5.33 | 2.03 | 0.157 | 0.31 |
| ror | -0.44 | 5.07 | 5.23 | 0.025 | 0.081 |
| PKA | 0.22 | 6.17 | 0.96 | 0.331 | 0.511 |
| start | 1.21 | 1.03 | 1.64 | 0.203 | 0.369 |
| smad3 | -0.1 | 4.77 | 0.28 | 0.599 | 0.747 |
| E78 | 0.61 | 4.49 | 0.51 | 0.476 | 0.646 |
| FTZ-F1 | -0.98 | 3.11 | 4.47 | 0.037 | 0.111 |
| melatonin rec | -0.18 | -0.2 | 0.06 | 0.806 | 0.888 |
| shade | 0.08 | 4.3 | 0.03 | 0.861 | 0.922 |
| dopamine rec 2 | 0.42 | -0.05 | 0.21 | 0.651 | 0.786 |
| PKC_x1 | -0.95 | 5.02 | 21.9 | 0 | 0 |
| dopamine rec 1 | 1.33 | -0.14 | 1.35 | 0.248 | 0.421 |
| sddp | 0.95 | 3.88 | 1.59 | 0.211 | 0.378 |
| ERK_1 | -0.06 | 5.03 | 0.08 | 0.779 | 0.871 |
| EcR | -0.05 | 5.58 | 0.02 | 0.88 | 0.935 |
| Pi3K | -0.4 | 5.58 | 8.46 | 0.005 | 0.023 |
| ERK_2 | -0.78 | 3.97 | 17.8 | 0 | 0.001 |
| disembodied | 0.79 | 1.32 | 1.84 | 0.179 | 0.339 |
| PTTH | -1.08 | -0.16 | 2.73 | 0.102 | 0.23 |
| DHR4 | 5.89 | 4.61 | 13.2 | 0 | 0.004 |
| PTSP | -0.48 | 1.92 | 0.74 | 0.392 | 0.57 |
| apc | -0.37 | 5.24 | 1.83 | 0.179 | 0.339 |
| krueppel | 0.32 | 2.3 | 0.12 | 0.728 | 0.839 |
| Insulin_receptor | -1.26 | 5.5 | 29.7 | 0 | 0 |
| DHR3 | 1.27 | 6.86 | 2.75 | 0.101 | 0.228 |
| ras | 0.44 | 6.15 | 13.3 | 0 | 0.004 |
| rolled_2 | -0.44 | 6.12 | 15.8 | 0 | 0.002 |
| ROS | 0.43 | 5.23 | 3.29 | 0.073 | 0.181 |
| mef2 | -0.93 | 6.2 | 14.4 | 0 | 0.003 |
| AKT | -0.14 | 6.18 | 0.24 | 0.622 | 0.765 |
| rolled_3 | 0.71 | 4.86 | 2.93 | 0.09 | 0.21 |
| 4E-BP | 0.24 | 6.46 | 0.44 | 0.51 | 0.673 |
| torso | 0.74 | 4.42 | 1.32 | 0.254 | 0.428 |
| mTOR | 0.03 | 5.01 | 0.03 | 0.868 | 0.927 |
| npc1 | -0.12 | 8.08 | 0.39 | 0.532 | 0.692 |
| PTEN | -0.35 | 3.97 | 4.79 | 0.031 | 0.097 |
| JHDK | 1.66 | 4.12 | 0.86 | 0.355 | 0.535 |

**Table S13: logFC, logCPM, F, pvalue, and FDR of the comparisons between diapause initiation (days 0, 3, 6) and diapause termination (days 114, 144).** Genes were considered significantly different when the false discovery rate (FDR, Benjamini-Hochberg method) was less than 0.05 and correspond to genes colored in red (upregulated) and blue (downregulated) in Figures 4 and 7 in the main text.

| **gene** | **logFC** | **logCPM** | **F** | **pvalue** | **FDR** |
| --- | --- | --- | --- | --- | --- |
| neverland | -0.42 | 2.1 | 0.35 | 0.558 | 0.664 |
| spook | 0.53 | 3.29 | 0.34 | 0.564 | 0.668 |
| FOXO | 0.01 | -0.37 | 0 | 0.981 | 0.989 |
| FOXO_x2 | -0.58 | 2.99 | 6.89 | 0.01 | 0.027 |
| plk1 | 1.59 | 4.41 | 9.35 | 0.003 | 0.01 |
| FTZ-F1beta | -0.25 | 4.87 | 0.69 | 0.408 | 0.528 |
| E74 | 1.4 | 3.8 | 17.67 | 0 | 0 |
| raf | 0.26 | 5.2 | 6.74 | 0.011 | 0.029 |
| E93 | -0.3 | 3.6 | 1.74 | 0.19 | 0.294 |
| ILP5 | -1.76 | -0.44 | 10.61 | 0.002 | 0.006 |
| ultraspiracle | -0.02 | 6.44 | 0.01 | 0.914 | 0.945 |
| frizzled | 0.62 | 4.38 | 3.78 | 0.055 | 0.108 |
| PTP61F | -0.15 | 6.48 | 0.54 | 0.464 | 0.582 |
| PKC_x6 | 1.15 | 3.66 | 7.07 | 0.009 | 0.025 |
| ILP3 | 0.24 | -0.77 | 0.1 | 0.749 | 0.82 |
| shadow | -0.96 | 3.62 | 19.76 | 0 | 0 |
| broad | -0.17 | 6.14 | 0.2 | 0.66 | 0.751 |
| CDK2 | 0.38 | 3.55 | 3.04 | 0.085 | 0.154 |
| shroud | 0.66 | 3.2 | 1.73 | 0.191 | 0.295 |
| wnt5 | 1.75 | 1.78 | 18.42 | 0 | 0 |
| rolled_4 | 0.09 | -0.31 | 0.02 | 0.887 | 0.924 |
| PECK1_2 | 1.14 | -1.11 | 1.71 | 0.194 | 0.299 |
| JHEH | -0.1 | 5.74 | 0.05 | 0.817 | 0.873 |
| svp | 0.11 | 4.35 | 0.12 | 0.734 | 0.809 |
| E75 | 0.47 | 2.13 | 3.12 | 0.081 | 0.148 |
| E75B | 0.48 | 1.48 | 1.43 | 0.235 | 0.346 |
| axin | -0.58 | 4.42 | 15.61 | 0 | 0.001 |
| wnt10b | 1.08 | -0.34 | 3.74 | 0.056 | 0.11 |
| wnt6 | -0.44 | 3.06 | 1.86 | 0.176 | 0.276 |
| wnt_1 | 0.31 | 1.59 | 0.55 | 0.461 | 0.579 |
| smad1 | -0.64 | 3.39 | 14.12 | 0 | 0.001 |
| serotonin trans | 2 | 1.52 | 11.37 | 0.001 | 0.004 |
| gsk3b | -0.01 | 6.28 | 0 | 0.952 | 0.971 |
| phantom | -1.02 | 5.33 | 16.41 | 0 | 0.001 |
| ror | -0.44 | 5.07 | 8.65 | 0.004 | 0.013 |
| PKA | -0.03 | 6.17 | 0.03 | 0.867 | 0.911 |
| start | 0.38 | 1.03 | 0.34 | 0.558 | 0.664 |
| smad3 | -0.34 | 4.77 | 5.52 | 0.021 | 0.049 |
| E78 | 1.36 | 4.49 | 4.4 | 0.039 | 0.081 |
| FTZ-F1 | -0.78 | 3.11 | 4.69 | 0.033 | 0.071 |
| melatonin_receptor | -0.37 | -0.2 | 0.43 | 0.511 | 0.624 |
| shade | -0.02 | 4.3 | 0 | 0.956 | 0.973 |
| dopamine rec 2 | 2.77 | -0.05 | 11.89 | 0.001 | 0.003 |
| PKC_x1 | -0.78 | 5.02 | 24.26 | 0 | 0 |
| dopa rec 1 | 0.38 | -0.14 | 0.25 | 0.618 | 0.715 |
| sddt | 0.55 | 3.88 | 1.05 | 0.309 | 0.427 |
| ERK_1 | -0.14 | 5.03 | 0.93 | 0.337 | 0.457 |
| EcR | -0.17 | 5.58 | 0.49 | 0.485 | 0.6 |
| Pi3K | -0.42 | 5.58 | 15.66 | 0 | 0.001 |
| ERK_2 | -0.6 | 3.97 | 17.62 | 0 | 0 |
| disembodied | -0.49 | 1.32 | 1.41 | 0.238 | 0.349 |
| PTTH | -1.47 | -0.16 | 8.25 | 0.005 | 0.015 |
| DHR4 | 4.2 | 4.61 | 19.95 | 0 | 0 |
| PTSP | 0.81 | 1.92 | 3.35 | 0.07 | 0.132 |
| apc | -0.15 | 5.24 | 0.53 | 0.467 | 0.584 |
| krueppel | 0.57 | 2.3 | 0.68 | 0.411 | 0.53 |
| Insulin_receptor | -0.71 | 5.5 | 15 | 0 | 0.001 |
| DHR3 | 1.63 | 6.86 | 8.46 | 0.005 | 0.014 |
| ras | 0.41 | 6.15 | 21.72 | 0 | 0 |
| rolled_2 | -0.69 | 6.12 | 65.69 | 0 | 0 |
| ROS | 0.41 | 5.23 | 5.45 | 0.022 | 0.051 |
| mef2 | -1.42 | 6.2 | 54.39 | 0 | 0 |
| AKT | 0.55 | 6.18 | 6.28 | 0.014 | 0.035 |
| rolled_3 | 0.72 | 4.86 | 5.63 | 0.02 | 0.047 |
| 4E-BP | -0.04 | 6.46 | 0.02 | 0.879 | 0.919 |
| torso | 2.7 | 4.42 | 27.53 | 0 | 0 |
| mTOR | -0.24 | 5.01 | 3.75 | 0.056 | 0.109 |
| npc1 | -0.56 | 8.08 | 14.48 | 0 | 0.001 |
| PTEN | -0.32 | 3.97 | 6.68 | 0.011 | 0.03 |
| JHDK | 0.55 | 4.12 | 0.24 | 0.628 | 0.724 |

**Table S14 logFC, logCPM, F, pvalue, and FDR of the comparisons between diapause initiation (days 0, 3, 6) and post-diapause development (days 155).** Genes were considered significantly different when the false discovery rate (FDR, Benjamini-Hochberg method) was less than 0.05 and corresponded to genes colored in red (upregulated) and blue (downregulated) in Figures 4 and 7 in the main text.

| **gene** | **logFC** | **logCPM** | **F** | **pvalue** | **FDR** |
| --- | --- | --- | --- | --- | --- |
| neverland | -0.5 | 2.1 | 0.32 | 0.575 | 0.7 |
| spook | -3.86 | 3.29 | 15.59 | 0 | 0.001 |
| FOXO | 1.36 | -0.37 | 7.73 | 0.007 | 0.022 |
| FOXO_x2 | 1.6 | 2.99 | 26.2 | 0 | 0 |
| plk1 | -1.9 | 4.41 | 11.23 | 0.001 | 0.005 |
| FTZ-F1beta | -0.34 | 4.87 | 0.86 | 0.357 | 0.497 |
| E74 | 1.05 | 3.8 | 6.1 | 0.015 | 0.043 |
| raf | 0.1 | 5.2 | 0.58 | 0.449 | 0.587 |
| E93 | -0.24 | 3.6 | 0.72 | 0.399 | 0.539 |
| ILP5 | -0.67 | -0.44 | 1 | 0.321 | 0.458 |
| ultraspiracle | 1.47 | 6.44 | 31.52 | 0 | 0 |
| frizzled | -0.21 | 4.38 | 0.28 | 0.597 | 0.718 |
| PTP61F | -0.07 | 6.48 | 0.06 | 0.801 | 0.873 |
| PKC_x6 | -0.21 | 3.66 | 0.16 | 0.693 | 0.795 |
| ILP3 | -0.84 | -0.77 | 0.91 | 0.341 | 0.481 |
| shadow | -0.78 | 3.62 | 8.66 | 0.004 | 0.015 |
| broad | 6.44 | 6.14 | 75.63 | 0 | 0 |
| CDK2 | -0.31 | 3.55 | 1.39 | 0.242 | 0.372 |
| shroud | 1.79 | 3.2 | 6.44 | 0.013 | 0.037 |
| wnt5 | -1 | 1.78 | 4.82 | 0.031 | 0.075 |
| rolled_4 | -2.1 | -0.31 | 9.54 | 0.003 | 0.01 |
| PECK1_2 | 0.65 | -1.11 | 0.37 | 0.543 | 0.673 |
| JHEH | -0.2 | 5.74 | 0.13 | 0.716 | 0.812 |
| svp | 0.62 | 4.35 | 2.1 | 0.151 | 0.261 |
| E75 | 0.09 | 2.13 | 0.07 | 0.797 | 0.87 |
| E75B | -1.21 | 1.48 | 6.56 | 0.012 | 0.035 |
| axin | -0.26 | 4.42 | 2.05 | 0.156 | 0.268 |
| wnt10b | -1.44 | -0.34 | 5.46 | 0.022 | 0.057 |
| wnt6 | -0.2 | 3.06 | 0.24 | 0.628 | 0.744 |
| wnt_1 | -1.36 | 1.59 | 7.59 | 0.007 | 0.023 |
| smad1 | 0.11 | 3.39 | 0.28 | 0.6 | 0.721 |
| serotonin trans | 0.59 | 1.52 | 0.68 | 0.41 | 0.55 |
| gsk3b | -0.3 | 6.28 | 1.58 | 0.212 | 0.337 |
| phantom | 1.35 | 5.33 | 14.63 | 0 | 0.001 |
| ror | -0.38 | 5.07 | 4.23 | 0.043 | 0.097 |
| PKA | -0.35 | 6.17 | 2.77 | 0.1 | 0.19 |
| start | -1.62 | 1.03 | 4.63 | 0.034 | 0.081 |
| smad3 | 0.49 | 4.77 | 6.51 | 0.012 | 0.036 |
| E78 | -2.68 | 4.49 | 14.84 | 0 | 0.001 |
| FTZ-F1 | 3.83 | 3.11 | 40.09 | 0 | 0 |
| melatonin rec | 2.07 | -0.2 | 6.25 | 0.014 | 0.04 |
| shade | 0.56 | 4.3 | 1.55 | 0.216 | 0.342 |
| dopa rec 2 | -0.66 | -0.05 | 0.67 | 0.416 | 0.556 |
| PKC_x1 | 0.48 | 5.02 | 5.27 | 0.024 | 0.061 |
| dopa rec 1 | -0.4 | -0.14 | 0.19 | 0.661 | 0.772 |
| sddt | -0.28 | 3.88 | 0.18 | 0.675 | 0.782 |
| ERK_1 | -0.51 | 5.03 | 7.85 | 0.006 | 0.021 |
| EcR | -0.4 | 5.58 | 1.84 | 0.179 | 0.296 |
| Pi3K | 0.03 | 5.58 | 0.05 | 0.83 | 0.893 |
| ERK_2 | -0.74 | 3.97 | 17.6 | 0 | 0 |
| disembodied | -0.94 | 1.32 | 3.66 | 0.059 | 0.126 |
| PTTH | 0.63 | -0.16 | 0.83 | 0.364 | 0.505 |
| DHR4 | -3.35 | 4.61 | 16.61 | 0 | 0.001 |
| PTSP | 0.3 | 1.92 | 0.3 | 0.585 | 0.707 |
| apc | -0.03 | 5.24 | 0.01 | 0.917 | 0.95 |
| krueppel | 2.06 | 2.3 | 4.38 | 0.039 | 0.091 |
| Insulin_receptor | 0.74 | 5.5 | 9.05 | 0.003 | 0.013 |
| DHR3 | -3.01 | 6.86 | 24.74 | 0 | 0 |
| ras | -0.58 | 6.15 | 28.87 | 0 | 0 |
| rolled_2 | -0.48 | 6.12 | 20.3 | 0 | 0 |
| ROS | 0.57 | 5.23 | 6.3 | 0.014 | 0.04 |
| mef2 | -0.15 | 6.2 | 0.38 | 0.537 | 0.668 |
| AKT | 0.94 | 6.18 | 10.7 | 0.002 | 0.007 |
| rolled_3 | -0.41 | 4.86 | 1.25 | 0.267 | 0.402 |
| 4E-BP | 0.72 | 6.46 | 4.09 | 0.046 | 0.104 |
| torso | 2.15 | 4.42 | 10.38 | 0.002 | 0.007 |
| mTOR | 0.06 | 5.01 | 0.13 | 0.72 | 0.815 |
| npc1 | 0.4 | 8.08 | 4.26 | 0.042 | 0.096 |
| PTEN | 0.06 | 3.97 | 0.13 | 0.716 | 0.812 |
| JHDK | 1.2 | 4.12 | 0.66 | 0.418 | 0.557 |

**Figure S15:** A**. PTTH protein levels in the brain and the abdomen of diapausing pupae.** The PTTH monomer is 12kDa and the PTTH dimer is 22kDa. The western blots were filled with 15µg protein per lane. The relative amount of PTTH within the time points was assessed with Ponceau S staining. B: protein levels of FoxO were assessed in the brain of directly developing pupae and pupae in diapause on day 0, 3 and 9 and diapause on day 54, 114, 148, 152 and 161 after pupation. Relative amounts of FoxO protein were assessed with Ponceau S staining [70].


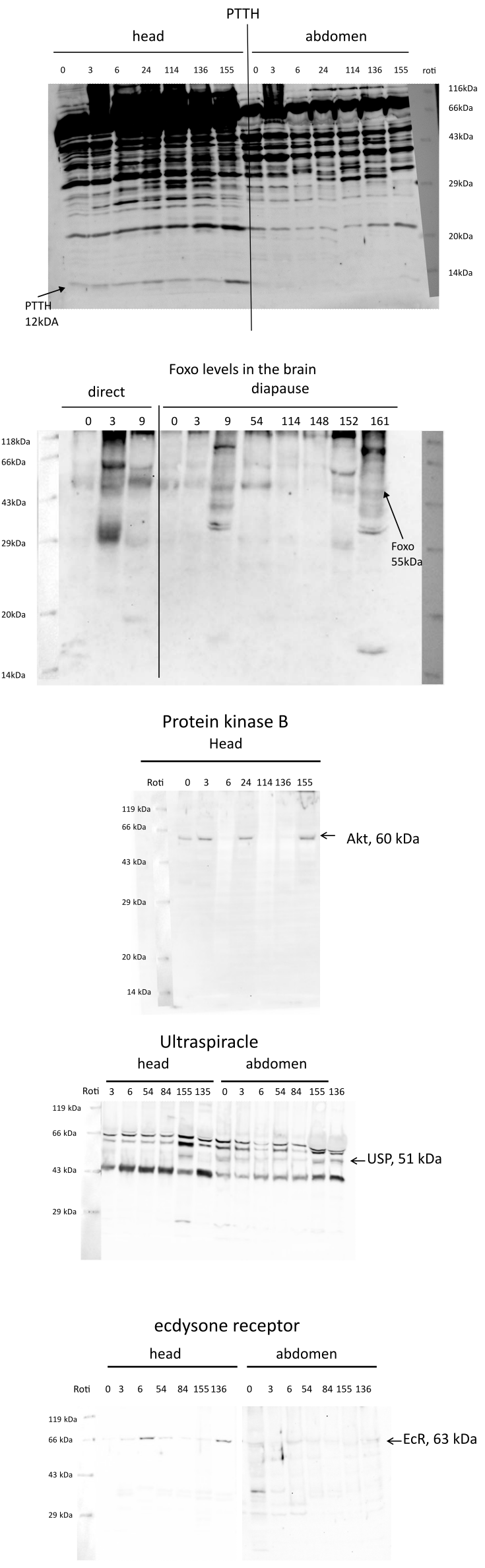


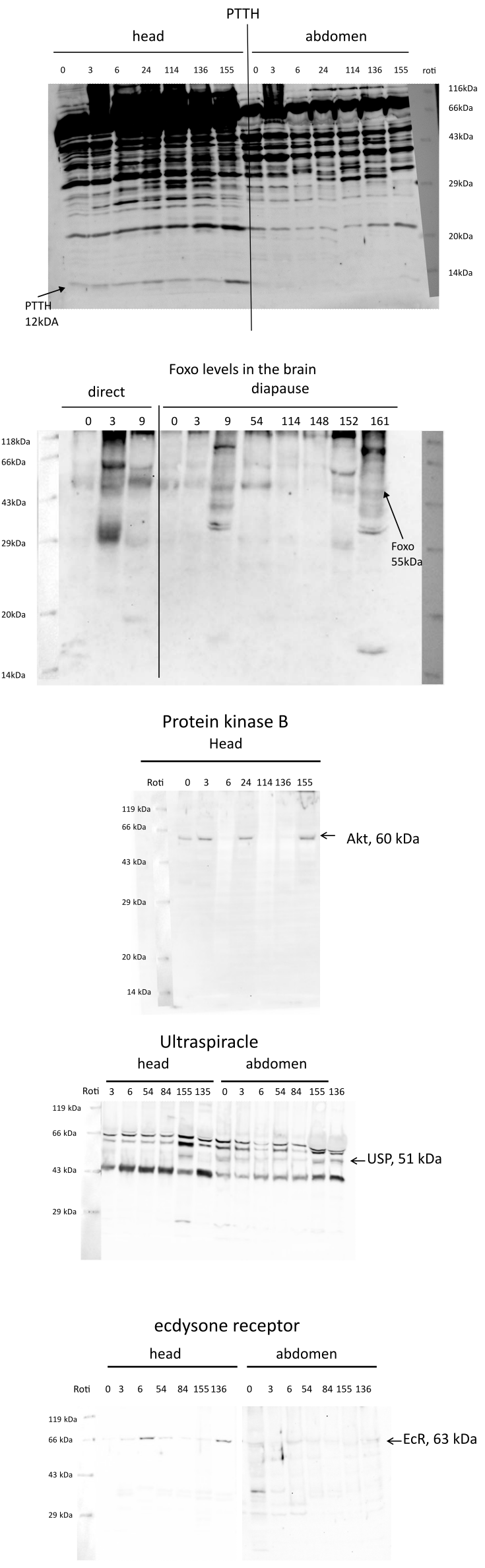

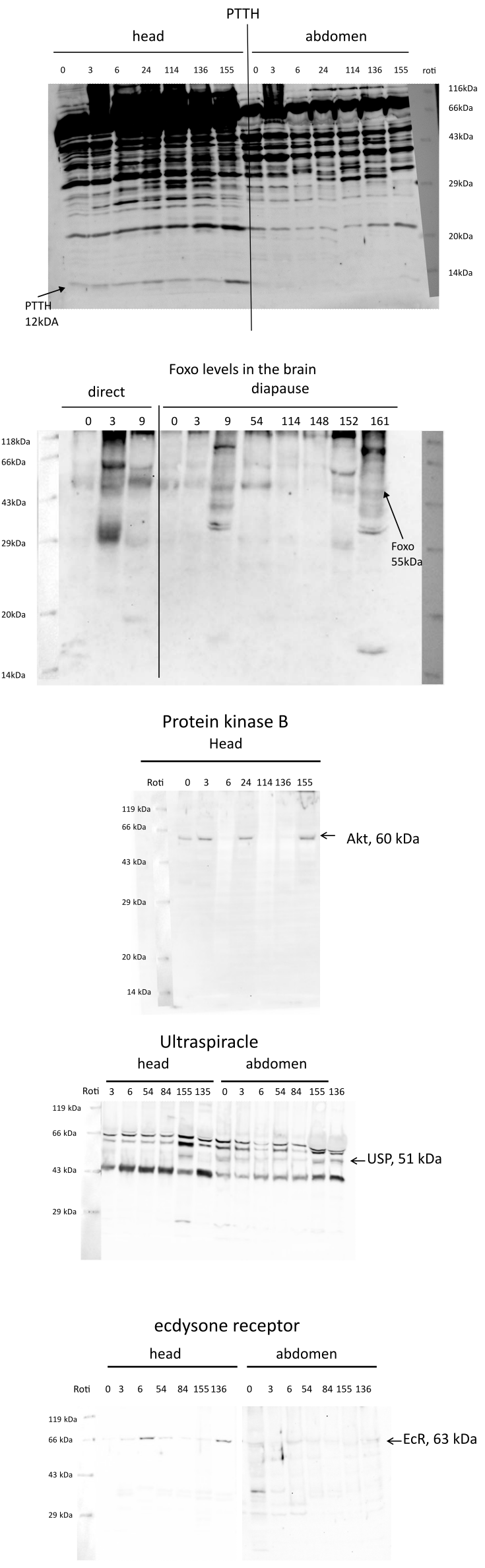


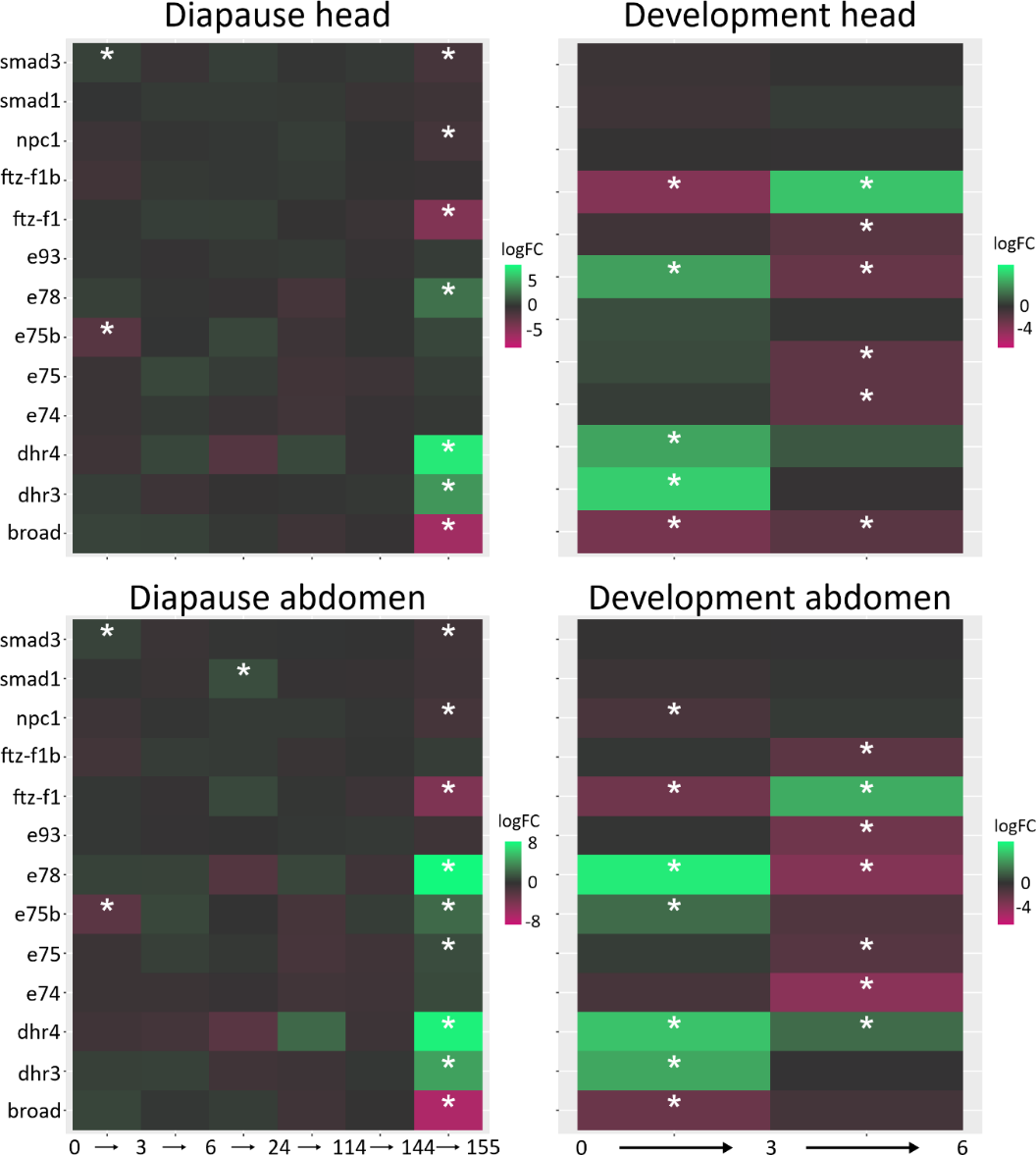


**S16: log fold change blots of pair-wise comparison of transcriptional levels of factors in the ecdysone-signaling pathway.** Comparisons are between day 0 and day 3 of diapause, day 3 and day 6, day 6 and day 24, day 24 and day 114, day 114 and day 144, and day 144 and day 155 of diapause on the left side with head on top and abdomen on the bottom and between day 0 and day 3 and day 3 and day 6 of direct development. Green indicates the upregulation of transcription on the later time point compared to the earlier time point, magenta indicates a downregulation. The white star indicates a significant change (corrected for multiple comparisons) in the transcription. Most of the significant transcriptional changes take place during post-diapause development and in the directly developing pupae, even though those samples are taken over only 6 days while the samples in diapause are over a time span of 144 days. This indicates the absence or low levels of ecdysone signaling in the diapause stage and therefore the absence of development in diapause.

**References**

1. R Core Team. R: A language and environment for statistical computing. 2013.

2. Kumar L, Futschik ME. Mfuzz: A software package for soft clustering of microarray data. Bioinformation. 2007;2:5–7. https://doi.org/10.6026/97320630002005.

3. Gu Z, Eils R, Schlesner M. Complex heatmaps reveal patterns and correlations in multidimensional genomic data. Bioinformatics. 2016;32:2847–9. https://doi.org/10.1093/bioinformatics/btw313.

4. Wickham H. Programming with ggplot2. In: Wickham H, editor. ggplot2: Elegant Graphics for Data Analysis. Cham: Springer International Publishing; 2016. p. 241–53. https://doi.org/10.1007/978-3-319-24277-4_12.

5. Mizoguchi A, Ohsumi S, Kobayashi K, Okamoto N, Yamada N, Tateishi K, et al. Prothoracicotropic Hormone Acts as a Neuroendocrine Switch between Pupal Diapause and Adult Development. PLOS ONE. 2013;8:e60824. https://doi.org/10.1371/journal.pone.0060824.

6. Süess P, Dircksen H, Roberts KT, Gotthard K, Nässel DR, Wheat CW, et al. Time- and temperature-dependent dynamics of prothoracicotropic hormone and ecdysone sensitivity co-regulate pupal diapause in the green-veined white butterfly Pieris napi. Insect Biochemistry and Molecular Biology. 2022;149:103833. https://doi.org/10.1016/j.ibmb.2022.103833.

7. Chen Z, Dong Y, Wang Y, Andongma AA, Rashid MA, Krutmuang P, et al. Pupal diapause termination in Bactrocera minax: an insight on 20-hydroxyecdysone induced phenotypic and genotypic expressions. Sci Rep. 2016;6:27440. https://doi.org/10.1038/srep27440.

8. Roe AD, Wardlaw AA, Butterson S, Marshall KE. Diapause survival requires a temperature-sensitive preparatory period. Current Research in Insect Science. 2024;5:100073. https://doi.org/10.1016/j.cris.2024.100073.

9. Denlinger DL. Confronting the challenges of a seasonal environment. In: Insect diapause. Cambridge University Press; 2022. p. 1–18.

10. Richard DS, Warrent JT, Saunders DS, Gilbert LI. Haemolymph ecdysteroid titres in diapause- and non-diapause-destined larvae and pupae of Sarcophaga argyrostoma. Journal of Insect Physiology. 1987;33:115–22. https://doi.org/10.1016/0022-1910(87)90083-7.

11. Williams CM. Ecdysone and ecdysone-analogues: their assay and action on diapausing pupae of the cynthia silkworm. The Biological Bulletin. 1968;134:344–55. https://doi.org/10.2307/1539610.

12. Koyama T, Rodrigues MA, Athanasiadis A, Shingleton AW, Mirth CK. Nutritional control of body size through FoxO-Ultraspiracle mediated ecdysone biosynthesis. eLife. 2014;3:e03091. https://doi.org/10.7554/eLife.03091.

13. Kelly GM, Lai C-J, Moon RT. Expression of Wnt10a in the Central Nervous System of Developing Zebrafish. Developmental Biology. 1993;158:113–21. https://doi.org/10.1006/dbio.1993.1172.

14. Smith W, Rybczynski R. Prothoracicotropic Hormone. In: Gilbert LI, editor. Insect Endocrinology. San Diego: Academic Press; 2012. p. 1–62. https://doi.org/10.1016/B978-0-12-384749-2.10001-9.

15. Kikuchi K, Niikura Y, Kitagawa K, Kikuchi A. Dishevelled, a Wnt signalling component, is involved in mitotic progression in cooperation with Plk1. The EMBO Journal. 2010;29:3470–83. https://doi.org/10.1038/emboj.2010.221.

16. Yamada N, Kataoka H, Mizoguchi A. Myosuppressin is involved in the regulation of pupal diapause in the cabbage army moth Mamestra brassicae. Sci Rep. 2017;7:41651. https://doi.org/10.1038/srep41651.

17. Henrich VC. 5 - The Ecdysteroid Receptor. In: Gilbert LI, editor. Insect Endocrinology. San Diego: Academic Press; 2012. p. 177–218. https://doi.org/10.1016/B978-0-12-384749-2.10005-6.

18. Petryk A, Warren JT, Marqués G, Jarcho MP, Gilbert LI, Kahler J, et al. Shade is the Drosophila P450 enzyme that mediates the hydroxylation of ecdysone to the steroid insect molting hormone 20-hydroxyecdysone. Proceedings of the National Academy of Sciences. 2003;100:13773–8. https://doi.org/10.1073/pnas.2336088100.

19. Rewitz KF, Rybczynski R, Warren JT, Gilbert LI. The Halloween genes code for cytochrome P450 enzymes mediating synthesis of the insect moulting hormone. Biochemical Society Transactions. 2006;34:1256–60. https://doi.org/10.1042/BST0341256.

20. Lafont R, Dauphin-Villemant C, Warren JT, Rees H. Ecdysteroid Chemistry and Biochemistry. In: Gilbert LI, editor. Insect Endocrinology. San Diego: Academic Press; 2012. p. 106–76. https://doi.org/10.1016/B978-0-12-384749-2.10004-4.

21. Warren JT, Petryk A, Marqués G, Parvy J-P, Shinoda T, Itoyama K, et al. Phantom encodes the 25-hydroxylase of Drosophila melanogaster and Bombyx mori: a P450 enzyme critical in ecdysone biosynthesis. Insect Biochemistry and Molecular Biology. 2004;34:991–1010. https://doi.org/10.1016/j.ibmb.2004.06.009.

22. Chavez VM, Marques G, Delbecque JP, Kobayashi K, Hollingsworth M, Burr J, et al. The Drosophila disembodied gene controls late embryonic morphogenesis and codes for a cytochrome P450 enzyme that regulates embryonic ecdysone levels. Development. 2000;127:4115–26. https://doi.org/10.1242/dev.127.19.4115.

23. Niwa R, Namiki T, Ito K, Shimada-Niwa Y, Kiuchi M, Kawaoka S, et al. Non-molting glossy/shroud encodes a short-chain dehydrogenase/reductase that functions in the ‘Black Box’ of the ecdysteroid biosynthesis pathway. Development. 2010;137:1991–9. https://doi.org/10.1242/dev.045641.

24. Ou Q, King-Jones K. What goes up must come down: transcription factors have their say in making ecdysone pulses. In: Shi Y-B, editor. Current Topics in Developmental Biology. Academic Press; 2013. p. 35–71. https://doi.org/10.1016/B978-0-12-385979-2.00002-2.

25. Uhlirova M, Foy BD, Beaty BJ, Olson KE, Riddiford LM, Jindra M. Use of Sindbis virus-mediated RNA interference to demonstrate a conserved role of Broad-Complex in insect metamorphosis. Proceedings of the National Academy of Sciences. 2003;100:15607–12. https://doi.org/10.1073/pnas.2136837100.

26. Calnan DR, Brunet A. The FoxO code. Oncogene. 2008;27:2276–88. https://doi.org/10.1038/onc.2008.21.

27. Antonova Y, Arik AJ, Moore W, Riehle MA, Brown MR. 2 - Insulin-Like Peptides: Structure, Signaling, and Function. In: Gilbert LI, editor. Insect Endocrinology. San Diego: Academic Press; 2012. p. 63–92. https://doi.org/10.1016/B978-0-12-384749-2.10002-0.

28. Kayukawa T, Nagamine K, Ito Y, Nishita Y, Ishikawa Y, Shinoda T. Krüppel Homolog 1 Inhibits Insect Metamorphosis via Direct Transcriptional Repression of Broad-Complex, a Pupal Specifier Gene *. Journal of Biological Chemistry. 2016;291:1751–62. https://doi.org/10.1074/jbc.M115.686121.

29. Zhang Z, Aslam AFM, Liu X, Li M, Huang Y, Tan A. Functional analysis of Bombyx Wnt1 during embryogenesis using the CRISPR/Cas9 system. Journal of Insect Physiology. 2015;79:73–9. https://doi.org/10.1016/j.jinsphys.2015.06.004.

30. Brazil DP, Yang Z-Z, Hemmings BA. Advances in protein kinase B signalling: AKTion on multiple fronts. Trends in Biochemical Sciences. 2004;29:233–42. https://doi.org/10.1016/j.tibs.2004.03.006.

31. Matsuzaki H, Ichino A, Hayashi T, Yamamoto T, Kikkawa U. Regulation of Intracellular Localization and Transcriptional Activity of FOXO4 by Protein Kinase B through Phosphorylation at the Motif Sites Conserved among the FOXO Family. The Journal of Biochemistry. 2005;138:485–91. https://doi.org/10.1093/jb/mvi146.

32. Grewal SS, York RD, Stork PJ. Extracellular-signal-regulated kinase signalling in neurons. Current Opinion in Neurobiology. 1999;9:544–53. https://doi.org/10.1016/S0959-4388(99)00010-0.

33. Roskoski R. ERK1/2 MAP kinases: Structure, function, and regulation. Pharmacological Research. 2012;66:105–43. https://doi.org/10.1016/j.phrs.2012.04.005.

34. Pfister TD, Storey KB. Insect freeze tolerance: Roles of protein phosphatases and protein kinase A. Insect Biochemistry and Molecular Biology. 2006;36:18–24. https://doi.org/10.1016/j.ibmb.2005.10.002.

35. Katewa SD, Kapahi P. Role of TOR signaling in aging and related biological processes in Drosophila melanogaster. Experimental Gerontology. 2011;46:382–90. https://doi.org/10.1016/j.exger.2010.11.036.

36. Hatting M, Tavares CDJ, Sharabi K, Rines AK, Puigserver P. Insulin regulation of gluconeogenesis. Annals of the New York Academy of Sciences. 2018;1411:21–35. https://doi.org/10.1111/nyas.13435.

37. Bruinsma W, Raaijmakers JA, Medema RH. Switching Polo-like kinase-1 on and off in time and space. Trends in Biochemical Sciences. 2012;37:534–42. https://doi.org/10.1016/j.tibs.2012.09.005.

38. Wicher D. Peptidergic Modulation of an Insect Na+ Current: Role of Protein Kinase A and Protein Kinase C. Journal of Neurophysiology. 2001;85:374–83. https://doi.org/10.1152/jn.2001.85.1.374.

39. Liu X, Tanaka Y, Song Q, Xu B, Hua Y. Bombyx mori prothoracicostatic peptide inhibits ecdysteroidogenesis in vivo. Archives of Insect Biochemistry and Physiology. 2004;56:155–61. https://doi.org/10.1002/arch.20005.

40. Song Z, Yang Y-P, Xu W-H. PTEN expression responds to transcription factor POU and regulates p-AKT levels during diapause initiation in the cotton bollworm, Helicoverpa armigera. Insect Biochemistry and Molecular Biology. 2018;100:48–58. https://doi.org/10.1016/j.ibmb.2018.06.005.

41. Yoshikawa S, McKinnon RD, Kokel M, Thomas JB. Wnt-mediated axon guidance via the Drosophila Derailed receptor. Nature. 2003;422:583–8. https://doi.org/10.1038/nature01522.

42. Doumpas N, Jékely G, Teleman AA. Wnt6 is required for maxillary palp formation in Drosophila. BMC Biology. 2013;11:104. https://doi.org/10.1186/1741-7007-11-104.

43. Wend P, Wend K, Krum SA, Miranda-Carboni GA. The role of WNT10B in physiology and disease. Acta Physiol (Oxf). 2012;204:34–51. https://doi.org/10.1111/j.1748-1716.2011.02296.x.

44. Inaki M, Yoshikawa S, Thomas JB, Aburatani H, Nose A. Wnt4 Is a Local Repulsive Cue that Determines Synaptic Target Specificity. Current Biology. 2007;17:1574–9. https://doi.org/10.1016/j.cub.2007.08.013.

45. Bhanot P, Brink M, Samos CH, Hsieh J-C, Wang Y, Macke JP, et al. A new member of the frizzled family from Drosophila functions as a Wingless receptor. Nature. 1996;382:225–30. https://doi.org/10.1038/382225a0.

46. Sansom OJ, Reed KR, Hayes AJ, Ireland H, Brinkmann H, Newton IP, et al. Loss of Apc in vivo immediately perturbs Wnt signaling, differentiation, and migration. Genes Dev. 2004;18:1385–90. https://doi.org/10.1101/gad.287404.

47. Kikuchi A. Roles of Axin in the Wnt Signalling Pathway. Cellular Signalling. 1999;11:777–88. https://doi.org/10.1016/S0898-6568(99)00054-6.

48. Nye DMR, Albertson RM, Weiner AT, Hertzler JI, Shorey M, Goberdhan DCI, et al. The receptor tyrosine kinase Ror is required for dendrite regeneration in Drosophila neurons. PLOS Biology. 2020;18:e3000657. https://doi.org/10.1371/journal.pbio.3000657.

49. Huang H, Regan KM, Lou Z, Chen J, Tindall DJ. CDK2-Dependent Phosphorylation of FOXO1 as an Apoptotic Response to DNA Damage. Science. 2006;314:294–7. https://doi.org/10.1126/science.1130512.

50. Wang Q, Mohamed AAM, Takeda M. Serotonin Receptor B May Lock the Gate of PTTH Release/Synthesis in the Chinese Silk Moth, Antheraea pernyi; A Diapause Initiation/Maintenance Mechanism? PLOS ONE. 2013;8:e79381. https://doi.org/10.1371/journal.pone.0079381.

51. Noguchi H, Hayakawa Y. Role of dopamine at the onset of pupal diapause in the cabbage armyworm Mamestra brassicae. FEBS Letters. 1997;413:157–61. https://doi.org/10.1016/S0014-5793(97)00848-X.

52. Richter K, Peschke E, Peschke D. Effect of melatonin on the release of prothoracicotropic hormone from the brain of Periplaneta americana (Blattodea: Blattidae). European Journal of Entomology. 1999;96:341–5.

53. Fletcher JC, Thummel CS. The Drosophila E74 gene is required for the proper stage- and tissue-specific transcription of ecdysone-regulated genes at the onset of metamorphosis. Development. 1995;121:1411–21. https://doi.org/10.1242/dev.121.5.1411.

54. D′Avino PP, Thummel CS. crooked legs encodes a family of zinc ﬁnger proteins required for leg morphogenesis and ecdysone-regulated gene expression during Drosophila metamorphosis. Development. 1998;125:1733–45. https://doi.org/10.1242/dev.125.9.1733.

55. Lam G, Hall BL, Bender M, Thummel CS. DHR3 Is Required for the Prepupal–Pupal Transition and Differentiation of Adult Structures during Drosophila Metamorphosis. Developmental Biology. 1999;212:204–16. https://doi.org/10.1006/dbio.1999.9343.

56. Ou Q, Magico A, King-Jones K. Nuclear Receptor DHR4 Controls the Timing of Steroid Hormone Pulses During Drosophila Development. PLOS Biology. 2011;9:e1001160. https://doi.org/10.1371/journal.pbio.1001160.

57. Ables ET, Bois KE, Garcia CA, Drummond-Barbosa D. Ecdysone response gene E78 controls ovarian germline stem cell niche formation and follicle survival in Drosophila. Developmental Biology. 2015;400:33–42. https://doi.org/10.1016/j.ydbio.2015.01.013.

58. Lavorgna G, Ueda H, Clos J, Wu C. FTZ-F1, a Steroid Hormone Receptor-like Protein Implicated in the Activation of fushi tarazu. Science. 1991;252:848–51. https://doi.org/10.1126/science.1709303.

59. Fluegel ML, Parker TJ, Pallanck LJ. Mutations of a Drosophila NPC1 Gene Confer Sterol and Ecdysone Metabolic Defects. Genetics. 2006;172:185–96. https://doi.org/10.1534/genetics.105.046565.

60. Wang C, Ma Z, Scott MP, Huang X. The cholesterol trafficking protein NPC1 is required for Drosophila spermatogenesis. Developmental Biology. 2011;351:146–55. https://doi.org/10.1016/j.ydbio.2010.12.042.

61. Truman JW. The Evolution of Insect Metamorphosis. Current Biology. 2019;29:R1252–68. https://doi.org/10.1016/j.cub.2019.10.009.

62. Teleman AA, Chen Y-W, Cohen SM. 4E-BP functions as a metabolic brake used under stress conditions but not during normal growth. Genes Dev. 2005;19:1844–8. https://doi.org/10.1101/gad.341505.

63. Tchankouo-Nguetcheu S, Udinotti M, Durand M, Meng T-C, Taouis M, Rabinow L. Negative regulation of MAP kinase signaling in Drosophila by Ptp61F/PTP1B. Mol Genet Genomics. 2014;289:795–806. https://doi.org/10.1007/s00438-014-0852-2.

64. Eivers E, Fuentealba LC, De Robertis E. Integrating positional information at the level of Smad1/5/8. Current Opinion in Genetics & Development. 2008;18:304–10. https://doi.org/10.1016/j.gde.2008.06.001.

65. Cuesto G, Jordán-Álvarez S, Enriquez-Barreto L, Ferrús A, Morales M, Acebes Á. GSK3β Inhibition Promotes Synaptogenesis in Drosophila and Mammalian Neurons. PLOS ONE. 2015;10:e0118475. https://doi.org/10.1371/journal.pone.0118475.

66. Zhang Q-R, Xu W-H, Chen F-S, Li S. Molecular and biochemical characterization of juvenile hormone epoxide hydrolase from the silkworm, Bombyx mori. Insect Biochemistry and Molecular Biology. 2005;35:153–64. https://doi.org/10.1016/j.ibmb.2004.10.010.

67. Maxwell RA, Welch WH, Schooley DA. Juvenile Hormone Diol Kinase: I. PURIFICATION, CHARACTERIZATION, AND SUBSTRATE SPECIFICITY OF JUVENILE HORMONE-SELECTIVE DIOL KINASE FROM MANDUCA SEXTA*. Journal of Biological Chemistry. 2002;277:21874–81. https://doi.org/10.1074/jbc.M201510200.

68. Shiomi K, Fujiwara Y, Atsumi T, Kajiura Z, Nakagaki M, Tanaka Y, et al. Myocyte enhancer factor 2 (MEF2) is a key modulator of the expression of the prothoracicotropic hormone gene in the silkworm, Bombyx mori. The FEBS Journal. 2005;272:3853–62. https://doi.org/10.1111/j.1742-4658.2005.04799.x.

69. Roth GE, Gierl MS, Vollborn L, Meise M, Lintermann R, Korge G. The Drosophila gene Start1: A putative cholesterol transporter and key regulator of ecdysteroid synthesis. Proceedings of the National Academy of Sciences. 2004;101:1601–6. https://doi.org/10.1073/pnas.0308212100.

70. Sander H, Wallace S, Plouse R, Tiwari S, Gomes AV. Ponceau S waste: Ponceau S staining for total protein normalization. Analytical Biochemistry. 2019;575:44–53. https://doi.org/10.1016/j.ab.2019.03.010.
